# Cyclic Ruthenium-Peptide Prodrugs Penetrate the Blood–Brain Barrier and Attack Glioblastoma upon Light Activation in Orthotopic Zebrafish Tumor Models

**DOI:** 10.1021/acscentsci.4c01173

**Published:** 2024-12-09

**Authors:** Liyan Zhang, Gangyin Zhao, Trevor Dalrymple, Yurii Husiev, Hildert Bronkhorst, Gabriel Forn-Cuní, Bruno Lopes-Bastos, Ewa Snaar-Jagalska, Sylvestre Bonnet

**Affiliations:** † Leiden Institute of Chemistry, Universiteit Leiden, Einsteinweg 55, 2333 CC Leiden, Netherlands; ‡ Leiden Institute of Biology, Universiteit Leiden, Einsteinweg 55, 2333 CC Leiden, Netherlands

## Abstract

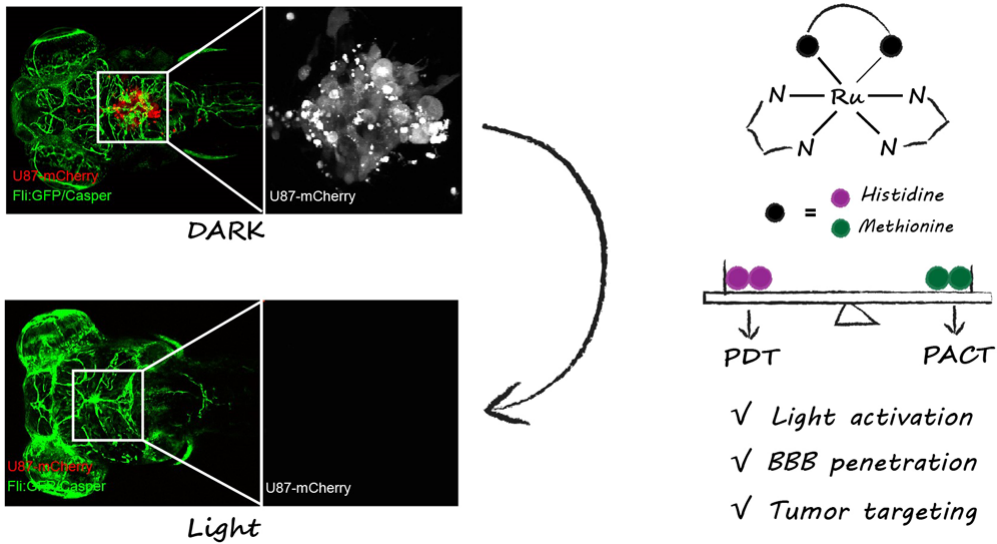

The blood–brain barrier (BBB) presents one of the main obstacles to delivering anticancer drugs in glioblastoma. Herein, we investigated the potential of a series of cyclic ruthenium-peptide conjugates as photoactivated therapy candidates for the treatment of this aggressive tumor. The three compounds studied, **Ru-p(HH)**, **Ru-p(MH)**, and **Ru-p(MM)** ([Ru(Ph_2_phen)_2_**(**Ac-X_1_RGDX_2_-NH_2_)]Cl_2_ with Ph_2_phen = 4,7-diphenyl-1,10-phenanthroline and X_1_, X_2_ = His or Met), include an integrin-targeted pentapeptide coordinated to a ruthenium warhead via two photoactivated ruthenium–X_1,2_ bonds. Their photochemistry, activation mechanism, tumor targeting, and antitumor activity were meticulously addressed. A combined *in vitro* and *in vivo* study revealed that the photoactivated cell-killing mechanism and their O_2_ dependence were strongly influenced by the nature of X_1_ and X_2_. **Ru-p(MM)** was shown to be a photoactivated chemotherapy (PACT) drug, while **Ru-p(HH)** behaved as a photodynamic therapy (PDT) drug. All conjugates, however, showed comparable antitumor targeting and efficacy toward human glioblastoma 3D spheroids and orthotopic glioblastoma tumor models in zebrafish embryos. Most importantly, in this model, all three compounds could effectively cross the BBB, resulting in excellent targeting of the tumors in the brain.

## Abstract 2

We show the excellent blood−brain barrier crossing properties, tumor targeting, and antitumor activity of three cyclic ruthenium-peptide phototherapeutic conjugates for the treatment of glioblastoma.

## 1 Introduction

Glioblastoma (GBM) stands out as one of the most formidable challenges in the realm of brain tumors; adding to the gravity of the situation is the disconcerting trend of increasing GBM diagnoses in recent years.^1^ Despite significant efforts directed toward developing chemotherapy and radiotherapy protocols for GBM, the prognosis for many patients remains dramatically low, characterized by debilitating side effects and constrained therapeutic efficacy.^2^ One of the factors contributing to this conundrum is the presence, in a healthy brain, of a highly selective blood–brain barrier (BBB) within the cerebral environment, which is poorly permeable to exogenous agents.^3,4^ High-grade (WHO grade III or IV) GBM patients often have a compromised BBB, which is why GBM tumors in these patients have enhanced contrast in magnetic resonance imaging. Further, standard-of-care radiation therapy permeabilizes the BBB, and at least with drugs like protoporphyrin IX (PPIX), endothelial cells that make up the BBB are damaged, which allows exogenous treatment to penetrate the tumor. The case of a lower-grade GBM is more complicated. For example, 5-aminolevulinic acid (5-ALA) treatment, which is metabolized into pink-emissive PPIX in GBM grade III or IV, now allows for efficient removal of the tumor by fluorescence-guided surgery. But low-grade GBM (WHO grade I or II) usually gives poor PPIX-based emission in 5-ALA-treated patients because of the intact BBB. Low-grade GBM regions are often not clearly visible in MRI, while the borders of high-grade GBM, which cannot be removed, are usually the origin of fatal postsurgery recurrences. Overall, in such regions of low-grade GBM, the BBB poses a challenge for adjuvant chemotherapy, obstructing the effective delivery of therapeutic drugs, thereby rendering medical treatment arduous.^5^ Considering the specific location of the tumor in GBM, it is imperative to closely monitor the ability of new (pro)drugs to distribute in the tumor and attack it.^5,6^ In the early stages of drug research, the selection of a judicious animal model capable of addressing the question of the BBB is of primary importance in evaluating the pharmacological potential of any new agent aimed at destroying a GBM tumor.^7,8^ Zebrafish embryos possess remarkable advantages, such as a brief growth cycle, cheap maintenance, immunological tolerance to human cancer cell lines, and optical transparency, which altogether allows for investigating drug safety, biodistribution, and antitumor efficacy in so-called “orthotopic” GBM models characterized by tumor localization in the brain.^9,10^ Remarkably, the BBB in zebrafish embryos functionally closes at 72 h postfertilization (dpf), which allows for designing antitumor efficacy studies in the presence or in the absence of the BBB, depending on the time of (pro)drug injection.^8^ Meanwhile, multiple studies have provided evidence that the zebrafish BBB is genetically and structurally similar to that of mice and humans and that BBB penetration studies in zebrafish embryo have predictive power with respect to larger animals.^11−13^

Even if the BBB could be crossed, GBM tumor cells are particularly resistant to chemotherapy, notably due to hypoxia-induced resistance mechanisms.^14^ The utilization of light-activated metal-based prodrugs, such as the ruthenium (Ru)-based compound TLD-1433,^15−17^ represents a recognized focal point in contemporary cancer research,^18^ while organic ones are making their way to clinical trial for the treatment of GBM.^19^ Light-activated anticancer prodrugs offer the advantage of precisely modulating their toxicity in space and time by focusing visible or near-infrared light irradiation on the prodrug-containing tumor.^20^ So far, two main cancer-killing mechanisms have been identified in light-activated ruthenium compounds: photodynamic therapy (PDT) and photoactivated chemotherapy (PACT).^21^ In PDT, the ruthenium prodrug is called a photosensitizer. Upon excitation by the light beam in oxygen-rich tissues, it generates high local doses of reactive oxygen species (ROS), which are cytotoxic and lead to tumor destruction.^22,23^ In contrast, PACT involves an oxygen-independent photochemical bond cleavage mechanism, which generates two photoproducts that subsequently interact with proteins or nucleic acids within cancer cells, inducing cell death and high local tumor toxicity.^24,25^ Interestingly, a ruthenium-based photosensitizer developed for PDT can be modified by chemical design into a PACT agent,^26^ while a few compounds were shown to work better by a combination of both mechanisms.^27^

In the pursuit of better PDT and PACT compounds, recent studies have conjugated ruthenium complexes to amino acid-based moieties such as peptides,^28^ peptoids,^29^ antibodies,^30^ or proteins^31^ to obtain active cancer targeting. These approaches enhance both the biocompatibility and the tumor selectivity of ruthenium complexes. The RGD small peptide^32,33^ and its conjugates^34,35^ have showed promising treatment efficacy for multiple high-grade glioma models. We recently demonstrated that integrin-targeted MRGDH peptides could be conjugated to light-activated ruthenium compounds via coordination of the methionine and histidine residues to the metal center. The resulting cyclic ruthenopeptide was extremely efficient at targeting cancer cells *in vitro*, while in subcutaneous GBM mice models (U87MG), the accumulation efficiency in tumor was observed as high as 15.7 ± 1.3%ID/g at 12 h after intravenous injection of the prodrug (injection dose = 7.7 mg/kg), thus triggering a strong antitumor effect upon green light irradiation.^36^ On the other hand, despite the ability of this complex to cleave both methionine and histidine residues upon light irradiation, it essentially behaved as a PDT molecule *in vitro* as it lost its activity in hypoxic conditions (1% O_2_) and generated high doses of ROS upon light irradiation. Furthermore, in subcutaneous GBM mice models, there is no BBB to cross; the efficacy shown in such a model has hence no predictive power about the ability of the compound to reach and destroy a tumor located inside the brain.

**Scheme 1:**
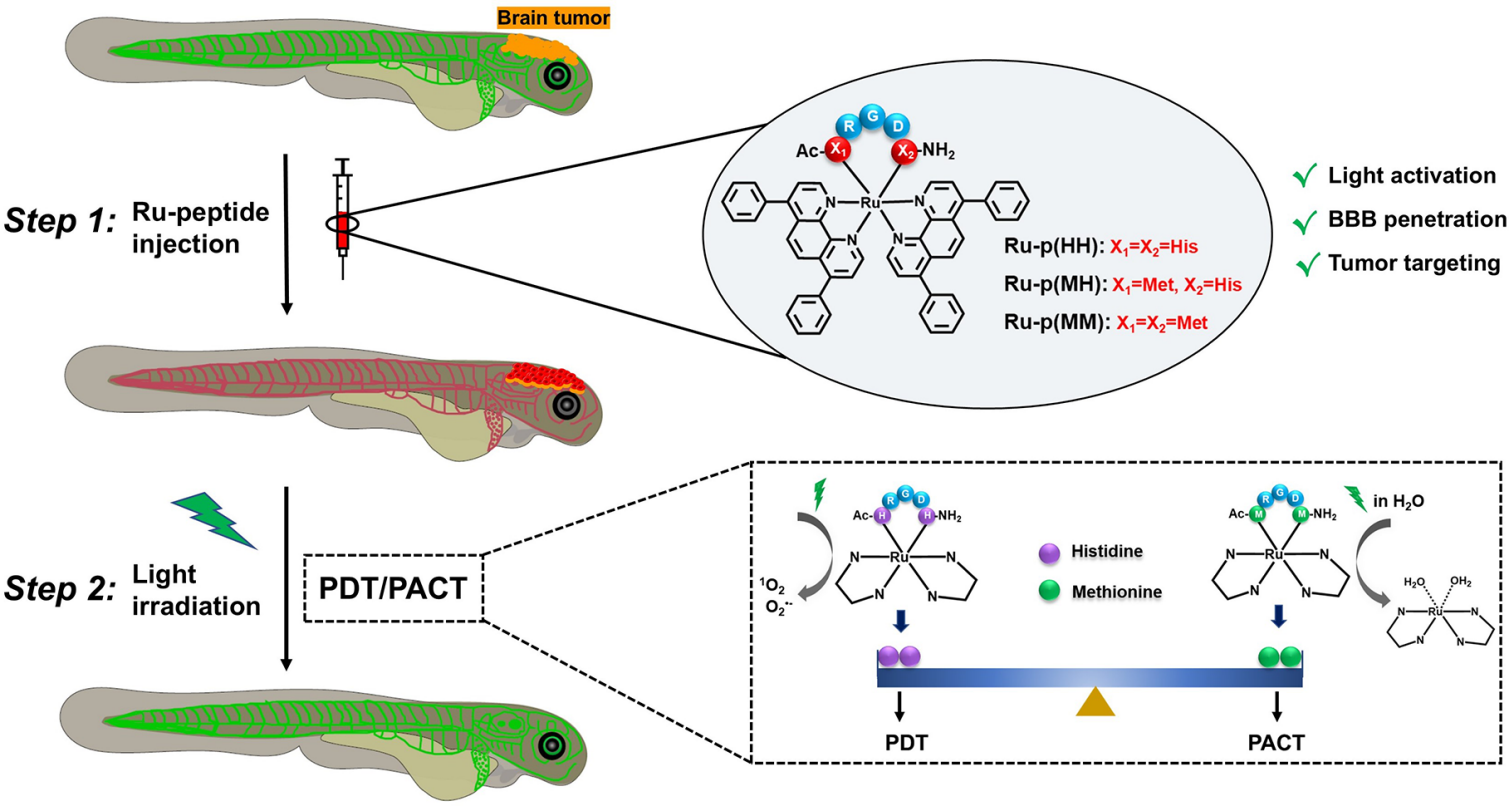
Ruthenium-Peptide Conjugates as Photoactivated Prodrugs for the Treatment of Brain Tumors in an Orthotopic Zebrafish Xenograft Model with a Mature Blood–Brain Barrier

In order to address these issues, we varied the nature of the amino acid bound to the metal and studied the resulting conjugates in an orthotopic GBM tumor model in zebrafish embryo with a functional BBB. Three ruthenium(II)-peptide conjugates were included in this study. The first complex [RuL_2_(Ac-MRGDH-NH_2_)]Cl_2_ (**Ru-p(MH)**, L = 4,7-diphenyl-1,10-phenanthroline) has already been described,^36^ but its analogues [RuL_2_(Ac-HRGDH-NH_2_)]Cl_2_ (**Ru-p(HH)**) and [RuL_2_(Ac-MRGDM-NH_2_)]Cl_2_ (**Ru-p(MM)**) were unknown. These two new compounds were prepared, purified, and characterized. The cancer targeting and cytotoxic properties of all three analogues were thoroughly compared *in vitro* and *in vivo* using a zebrafish U87MG tumor model, both without and with a mature BBB. It is shown that all three ruthenium-peptide conjugates can realize exceptional antitumor effects toward glioblastoma, with the capability of BBB penetration. Notably, altering the coordination amino acid sites of the ruthenium-peptide conjugates modulates the mechanism of photoactivation pathways (Scheme 1).

## 2 Results

### 2.1 Synthesis and Characterization

**Figure 1:**
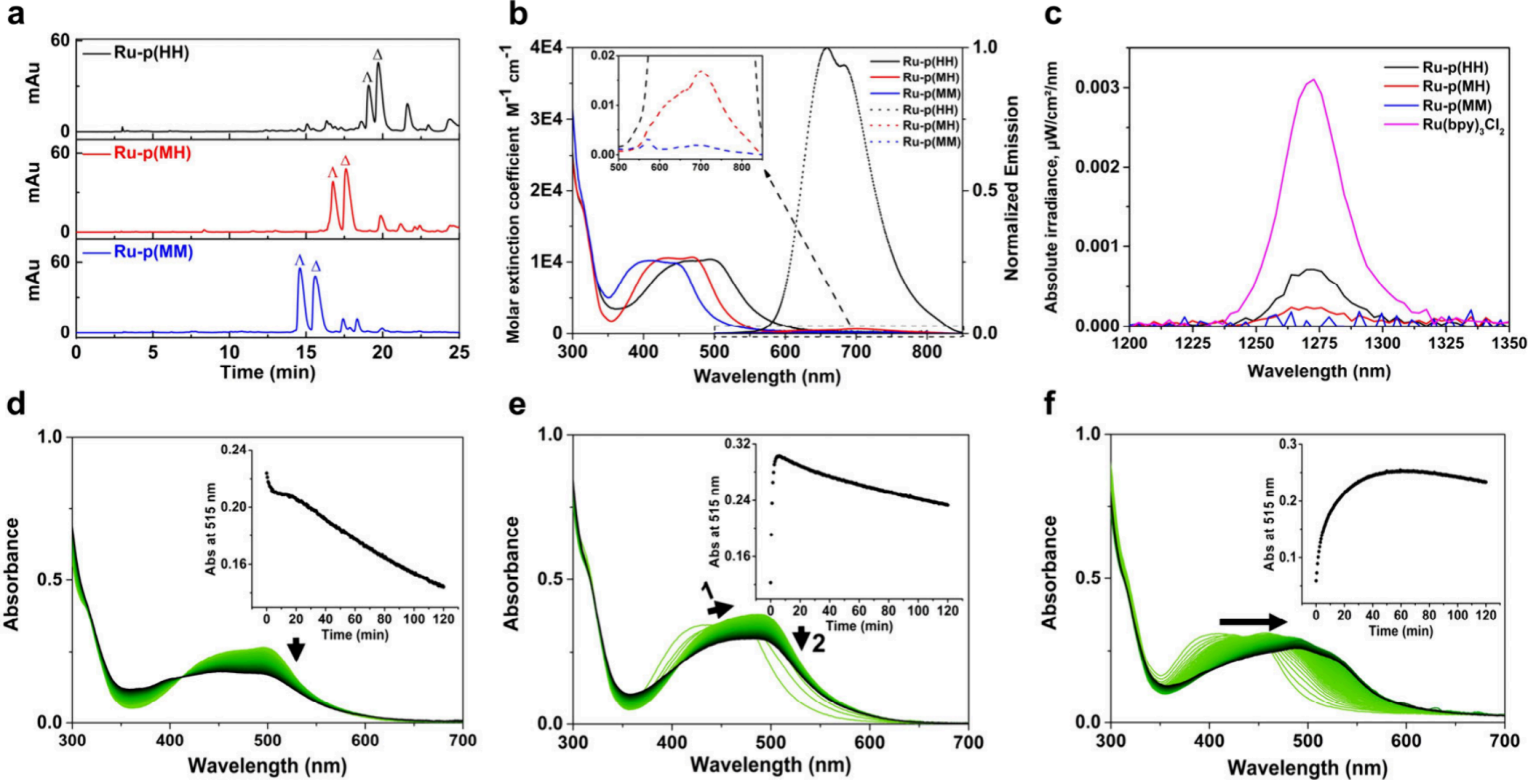
Characterization of Ru-peptide conjugates **Ru-p(HH)**, **Ru-p(MH)**, and **Ru-p(MM)**. (a) HPLC traces of the crude reaction mixtures. Gradient used: 25–35% acetonitrile/water, 20 min, flow rate = 14 mL/min, collection UV channel = 290 nm. (b) Molar extinction coefficients (M^–1^ cm^–1^, solid line), emission spectra (normalized to the maximum of **Ru-p(HH)**, dashed line) and (c) normalized near-infrared spectroscopy (NIR) emission from ^1^O_2_ generated by **Ru-p(HH)**, **Ru-p(MH)**, and **Ru-p(MM)** under blue light irradiation (450 nm) in CD_3_OD. The prototypical [Ru(bpy)_3_]Cl_2_ complex was used as a reference. (d–f) Time evolution of the UV–vis spectra of (d) **Ru-p(HH)**, (e) **Ru-p(MH)**, and (f) **Ru-p(MM)** in H_2_O under green light irradiation (515 nm, 4.0 mW cm^–2^) for 2 h. Insets: plots of absorbance at 515 nm vs irradiation time.

The synthesis of **Ru-p(HH)** and **Ru-p(MM)** was performed according to the method described for **Ru-p(MH)**.^36^ In short, the reaction of the racemic chiral ruthenium precursor [RuL_2_Cl_2_] and an enantiomerically pure peptide Ac-HRGDH-NH_2_ or Ac-MRGDM-NH_2_ composed of l-amino acids only produced two diastereoisomers that were separated by high-performance liquid chromatography (HPLC). According to the integral area of both HPLC peaks and isolated yields, for **Ru-p(HH)** and **Ru-p(MM)** the ratio between both Λ and Δ diastereoisomers was 1:2 and 1:1, respectively, while for **Ru-p(MH)** a 1:1.5 ratio of isomers had been obtained (Figure 1a). We suspect here that the higher rigidity of histidine residues, compared to methionine,^37^ promoted the formation of a higher fraction of the Δ isomer, while for the more flexible **Ru-p(MM)** conjugate, there was little to no energetic preference for one over the other diastereoisomer, thus leading to a statistical mixture. For **Ru-p(MH)**, both diastereoisomers had been isolated and characterized separately in our previous report,^36^ but they had essentially the same chemical and photochemical properties, while isolation of the pure isomers led to a significantly decreased preparative yield. In all photochemical and biological experiments reported below, we hence purified **Ru-p(HH)**, **Ru-p(MH)**, and **Ru-p(MM)** from other impurities but kept them as a 1:2, 1:1.5, or 1:1 mixture of Λ/Δ diastereoisomers, which allowed us to obtain them in acceptable preparative yields (28%, 27%, and 15%, respectively). The final HPLC traces are reported in Figures S5 and S6.

### 2.2 Photochemical Study

The photochemical properties of the three conjugates were compared by using a combination of techniques. Absorbance spectra in Milli-Q water (solid lines in Figure 1b, Figure S11, Table 1) showed that all complexes had a metal-to-ligand charge transfer (^1^MLCT) band between 400 and 600 nm, which is favorable for visible light activation. In this series of compounds, more Met residues bound to ruthenium led to a blue shift of the MLCT band compared to more His residues. There was barely any difference in the molar extinction coefficient (M^–1^ cm^–1^) at the absorption maximum between the three compounds. By contrast, the emission spectra of the three complexes upon excitation at 480 nm were very different (dashed lines in Figure 1b). **Ru-p(HH)** generated a strong emission around 660 nm, while the other two complexes showed very weak emission, especially **Ru-p(MM)**. In the same conditions, the relative intensity of **Ru-p(MH)** and **Ru-p(MM)** was only 2% and 0.2%, respectively, compared to that of **Ru-p(HH)** (Table 1). Correspondingly, the singlet oxygen (^1^O_2_) generation quantum yield of **Ru-p(HH)** was significantly higher (Φ_Δ_ = 0.183) compared to that of **Ru-p(MH)** (Φ_Δ_ = 0.048), while **Ru-p(MM)** did not give rise to any significant ^1^O_2_ production (Φ_Δ_ = 0.013, see Figure 1c and Table 1). Considering these values, **Ru-p(HH)** may be photoactive according to a photodynamic mechanism, while **Ru-p(MM)** should not be.

**Table 1:** Photochemical Data for Ru-Peptide Conjugates

complex	ε (M^–1^ cm^–1^)/λ_max_ (nm)^a^^,^^b^	λ_em_ (nm)/relative intensity^a^^,^^c^	Φ_Δ_^d^	Φ_PS1_^a^^,^^e^	Φ_PS2_^a^^,^^e^	^3^MLCT (eV)	^3^MC (eV)
**Ru-p(HH)**	1.08 × 10^4^/500	658/1.0	0.183 ± 0.007	0.0014	/	2.10	2.11
**Ru-p(MH)**	1.12 × 10^4^/465	700/0.02	0.048 ± 0.021	0.133	0.0005	2.08	1.97 (Met)/2.17 (His)^f^
**Ru-p(MM)**	1.06 × 10^4^/400	700/0.002	0.013 ± 0.005	0.151	0.0052	2.32	2.00

a Measurements were carried out in Milli-Q H_2_O.

b Molar absorption coefficients (M^–1^ cm^–1^) were obtained according to Figure S11 at the wavelength λ_max_.

c Emission intensity was normalized to that of **Ru-p(HH)**; all complexes were dissolved at a concentration of 80 μM using λ_ex_ = 480 nm.

d Quantum yields of singlet oxygen generation were measured by using [Ru(bpy)_3_]Cl_2_ complex as reference (Φ_Δ_^ref^ = 73%)^38^ with two independent measurements.

e Φ_PS_ values of single Λ- and Δ-**Ru-p(MH)** isomer have been reported previously.^36^

f Met represents the ^3^MC state energy of **Ru-p(MH)** when methionine photodissociates first; His represents the ^3^MC state energy of **Ru-p(MH)** when histidine photodissociates first.

All three complexes were first tested in the dark in Milli-Q water solution at 25 °C: in such conditions, they were all found to be thermally stable for at least 24 h (Figure S12). The possible aggregation of the complexes in cell culture medium was also studied by dynamic light scattering (DLS, Figure S21). For **Ru-p(HH)** and **Ru-p(MM)**, as reported for **Ru-p(MH)**,^36^ ∼10–100 nm particles were observed when either complex was dissolved in Opti-MEM cell culture medium containing 2.5% fetal calf serum (FCS). The similar observations made for all three complexes proved that the polar RGD amino acids and the hydrophobic [Ru(L)_2_]^2+^ moiety probably play an essential role in the aggregation of these molecules in cell culture medium. Once characterized in the dark, a photosubstitution study was conducted by monitoring the absorbance spectra of the three complexes in H_2_O under green light activation (515 nm, 4.0 mW cm^–2^, 2 h). As a note, thioether ligands are known to be weaker σ-donors and weaker π-acceptors compared to imine ligands,^37^ which predicts the ^3^MC excited state to be lower for **Ru-p(MM)** than for **Ru-p(HH)**. Indeed, **Ru-p(MM)** showed significant changes of its absorption spectra during light irradiation (Figure 1f), as reported for **Ru-p(MH)** (Figure 1e),^36^ which suggested that ligand disassociation occurred for **Ru-p(MM)** as well. Mass spectra confirmed the production of the bis-aqua photoproduct [Ru(Ph_2_phen)_2_(H_2_O)_2_]^2+^ (found *m*/*z* = 424.2, calc. *m*/*z* = 424.1 for the formic acid adduct) upon irradiation of **Ru-p(MM)** with green light. In the same irradiation conditions, **Ru-p(HH)** showed only one photosubstitution with a H_2_O molecule (Figure 1d), leading to the semi-opened photoproduct [Ru(Ph_2_phen)_2_(η^1^-Ac-HRGDH-NH_2_)(H_2_O)]^3+^ (found *m*/*z* = 492.8, calc. *m*/*z* = 492.8 for the MeOH adduct, see Figures S13 and S14). Accordingly, the rate of photocleavage differed strongly between both compounds, as quantified by photosubstitution quantum yield measurements (Φ_PS_, see Figures S15 and S16, Table 1). **Ru-p(HH)** generated a single product in one step with the lowest quantum yield Φ_PS_ = 0.0014. For **Ru-p(MM)**, fitting was possible using a two-step photoreaction, like for **Ru-p(MH)**. The photosubstitution quantum yield of the first step Φ_PS1_ of **Ru-p(MM)** was similar to that of **Ru-p(MH)**, but for the second step, Φ_PS2_ was 10 times higher for **Ru-p(MM)** than for **Ru-(MH)**. Clearly, methionines were photosubstituted faster than histidines on these types of ruthenium complexes. As a consequence, **Ru-p(MM)** was predicted to be photoactivated in cells as well according to a PACT mechanism. Photosubstitution studies were also followed by HPLC in acetonitrile like for the published **Ru-p(MH)** conjugate.^36^ Compared with H_2_O, acetonitrile represents a stronger coordination ligand that can better simulate the numerous biologically occurring ligands that may bind to Ru after photosubstitution of the RGD peptide. As shown in Figures S17 and S18, when **Ru-p(HH)** and **Ru-p(MM)** were irradiated with green light for 30 min in MeCN, the starting complexes had almost or totally disappeared, and 2–3 new peaks were observed, which represented Ru-containing photoproducts where 1 or 2 residues of the peptide had been substituted by MeCN, confirming the photosubstitution mode of activation of these complexes.

To rationalize the different reactivities of **Ru-p(HH)** and **Ru-p(MM)**, the relative energies of the ^3^MLCT and ^3^MC states of the three conjugates were calculated by DFT at the PBE0/TZP/COSMO level in water. As shown in Table 1, for **Ru-p(MM)** the ^3^MC state was found to be lower than the ^3^MLCT, with a rather high energy stabilization Δ*E* = *E*(^3^MLCT) – *E*(^3^MC) = 0.32 eV. For **Ru-p(HH)**, both triplet states were almost at the same energy level (Δ*E* = −0.01 eV). For **Ru-p(MH)**, two ^3^MC states were found, one with an elongated Ru–S bond at a rather low energy (Δ*E* = +0.11 eV), corresponding to the photosubstitution of Met, and one with an elongated Ru–N(His) bond at a higher energy compared to ^3^MLCT (Δ*E* = −0.09 eV), leading to the photosubstitution of His. Altogether, these modeling results suggested that **Ru-p(MM)** and **Ru-p(MH)** should be more prone to deactivate via the ^3^MC states leading to photosubstitution and hence act as PACT agents, while **Ru-p(HH)** might have a higher energy barrier to the ^3^MC state, thereby favoring phosphorescence emission or ^1^O_2_ generation from the ^3^MLCT state and hence having a PDT character. For **Ru-p(MH)**, the lower energy gap Δ*E* for releasing Met predicted that the Ru–S bond should be broken first.

### 2.3 Integrin α_V_β_3_ and α_V_β_5_ Expression and Targeting *In Vitro*

**Figure 2:**
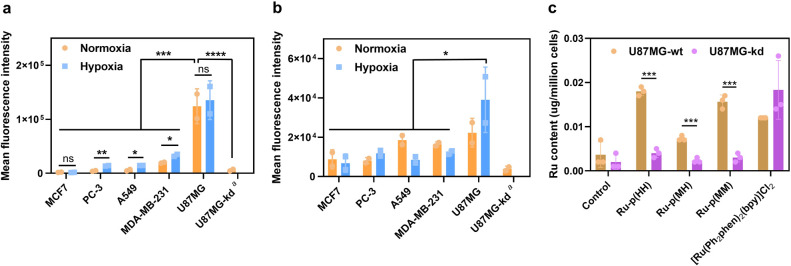
Integrin targeting by **Ru-p(HH)**, **Ru-p(MH)**, and **Ru-p(MM)***in vitro*. (a, b) Expression of integrin (a) α_V_β_3_ and (b) α_V_β_5_ in MCF7, PC-3, A549, MDA-MB-231, U87MG, and U87MG-kd cell lines under normoxic (21% O_2_) or hypoxic (1% O_2_) conditions. Note a: ITGAV was knockdown; see Methods. Bars represent the fluorescence intensity of the cells incubated with either anti-integrin α_V_β_3_ or α_V_β_5_ monoclonal antibodies followed by a secondary antibody conjugated to Alexa-Fluor 488. Error: standard deviation (SD), *n* = 2. Representative histograms of the control and cell group are shown in Figures S19 and S20. (c) Ruthenium accumulation (μg Ru/million cells) of wild type U87MG (U87MG-wt) and ITGAV knockdown U87MG (U87MG-kd) cell lines after treatment with medium (control), **Ru-p(HH)**, **Ru-p(MH)**, **Ru-p(MM)**, or **[Ru(Ph**_**2**_**phen)**_**2**_**(bpy)]Cl**_**2**_ (normoxia, 10 μM, 3 h). Error: standard deviation (SD), *n* = 3. Two-way ANOVA was used to determine the significance of the comparisons of data (*P < 0.05, **P < 0.01, ***P < 0.001, ****P < 0.0001).

As the target of the RGD sequence, two typical integrin heterodimers, α_V_β_3_ and α_V_β_5_, were selected for quantifying integrin expression in five different cell lines: A549 (human adenocarcinoma alveolar basal epithelial cells), MDA-MB-231 (human breast cancer cells), PC-3 (human prostate cancer cells), U87MG (human primary glioblastoma), and MCF7 (human breast carcinoma) using a double-immunofluorescence protocol.^39^ It has been reported that RGD-related integrins are involved in the cell response to hypoxia,^40^ which is not only the most significant barrier for PDT so far but also associated with resistance to a number of anticancer agents.^41,42^ For RGD-related integrin, the upregulation expression may offer a potential perspective for targeting the hypoxic region of tumor cells.^43^ Thus, the integrin expression levels of the five cell lines in normoxic (21% O_2_) or hypoxic conditions (1% O_2_) were included in this study and quantified by flow cytometry. The corresponding histograms can be found in Figures S19 and S20, and the mean fluorescent intensities, or expression level, are shown in Figure 2. Accordingly, significant variation in integrin expression was observed: U87MG possessed by far the highest α_V_β_3_ expression, compared to other cell lines, and this for both heterodimers and in both normoxic and hypoxic conditions. Interestingly, higher α_V_β_3_ integrin expression was observed in hypoxia for MDA-MB-231, A549, and PC-3 cell lines. For α_V_β_5_, the integrin expression in all cell lines was more balanced, with no statistically significant difference between normoxia vs hypoxia. Overall, glioblastoma cells (U87MG) appeared as the cell line offering the highest integrin expression in both normoxia and hypoxia. Glioblastoma is one of the most aggressive forms of cancers; it starts inside the brain, and fewer than 5–10% of patients survive in 5 years after diagnosis.^44^ U87MG cells were hence chosen for a further comparison of the biological properties of **Ru-p(HH)**, **Ru-p(MH)**, and **Ru-p(MM)** compounds.

In order to check whether the integrins α_V_β_3_ and α_V_β_3_ were responsible for the cellular uptake of these conjugates, an ITGAV (integrin α_V_) knockdown U87MG cell line (U87MG-kd, Figure 2a,b) was prepared, and the cellular ruthenium uptake was measured using ICP-MS in wild type and knockdown U87MG 2D cell monolayers (Figure 2c). For all three ruthenium analogues, the intracellular accumulation of Ru in U87MG-kd was significantly reduced compared to wild type U87MG cells. When performing the same experiment with the control ruthenium complex **[Ru(Ph_2_phen)_2_(bpy)]Cl_2_** (bpy = 2,2'-bipyridine) that bore the same charge as **Ru-p(HH)**, **Ru-p(MH)**, and **Ru-p(MM)** and was also thermally stable but was deprived of RGD peptide, the cellular uptake was not lowered in U87MG-kd compared to wild type U87MG. These results clearly demonstrated that the uptake of **Ru-p(HH)**, **Ru-p(MH)**, and **Ru-p(MM)** was integrin-dependent, and hence, the ruthenium complexes were indeed targeting integrins at the cell surface *in vitro*.

### 2.4 Anticancer Study on 2D Monolayer Cells

To test the *in vitro* phototoxicity of the three complexes toward 2D monolayer U87MG cells, a cell viability assay was undertaken as follows: the cells were seeded at *t* = 0, treated at *t* = 24 h, irradiated with green light (520 nm, 13.1 J cm^–2^) at *t* = 48 h without refreshing the medium (drug-to-light interval = 24 h), further incubated in the dark for 48 h, and counted at *t* = 96 h with a sulforhodamine B (SRB) cell quantification end point assay.^45^ Half-maximal effective concentrations (EC_50_ in μM), compared to untreated control, and photoindex (PI) values, defined as EC_50,dark_/ EC_50,light_, were calculated to characterize the toxicity and light activation of each Ru-peptide conjugate. In normoxic conditions (21% O_2_), both complexes **Ru-p(HH)** and **Ru-p(MM)** exhibited toxicity levels in the light group as high as those of **Ru-p(MH)** (Figure S22a, Table 2). The EC_50,light_ values were ∼1–3 μM, while in the dark the EC_50,dark_ values were between 20 and 40 μM, resulting in PI values for **Ru-p(HH)**, **Ru-p(MH)**, and **Ru-p(MM)** of 12.1, 11.9, and 8.5, respectively. The dark toxicity of these complexes was significantly lower than that of the hydrophobic [RuL_2_Cl_2_] control complex, which hydrolyzes spontaneously and showed an EC_50_ of 1.43 μM in normoxic U87MG cells under the same conditions. The low dark toxicity and significantly higher light toxicity of all three Ru-peptide conjugates corroborated the initial design of these photoactive compounds. However, a much stronger discrepancy appeared in the 2D toxicity data in hypoxic conditions (Table 2, Figure S22b). The phototherapeutic properties of **Ru-p(HH)** in the green light group were the poorest, leading to a PI of only 1.3. This effect of hypoxia was less significant with **Ru-p(MM)**, which possessed a PI value of 4.0, demonstrating better light activation compared to **Ru-p(HH)**. **Ru-p(MH)** stood somewhere between these values with a PI of 1.9 under hypoxia. According to these data, the two methionine coordinated ruthenium complex retained a higher PI in hypoxia, compared to peptides attached via two histidines. The loss of activity of **Ru-p(HH)** under hypoxia is consistent with the hypothesis of a PDT pathway, while **Ru-p(MM)** keeping appreciable phototoxicity under hypoxia better fits the hypothesis of a PACT cell-killing mechanism.

**Table 2:** Cytotoxicity of Ru-Peptide Conjugates: Half-Maximal Effective Concentrations (EC_50_ in μM) and 95% Confidence Intervals (CI_95_ in μM, *n* = 3) for **Ru-p(HH)**, **Ru-p(MH)**, and **Ru-p(MM)** in the Dark or upon Green Light Irradiation in 2D Monolayers of U87MG Cell Lines under Normoxic (21% O_2_) and Hypoxic (1% O_2_) Conditions and in 3D U87MG Spheroids under Normoxic Conditions^a^^,^^b^^,^^c^

		**Ru-p(HH)**			**Ru-p(MH)**			**Ru-p(MM)**		
O_2_ condition	green light dose (J cm^–2^)	EC_50_ (μM)	±CI (95%)	PI	EC_50_ (μM)	±CI (95%)	PI	EC_50_ (μM)	±CI (95%)	PI
2D, normoxia (21% O_2_)	0	20.6	–6.0	12.1	35.8	–8.8	11.9	22	–6.3	8.5
			+10.5			+16.8			+/	
	13.1	1.7	–0.7		3.0	–0.6		2.6	–0.6	
			+1			+0.6			+0.6	
2D, hypoxia (1% O_2_)	0	19.4	–3.2	1.3	34.9	–2.9	1.9	24.2	–5.3	4.0
			+4.1			+3.4			+6.1	
	13.1	15.4	–3.7		18.0	–1.7		6.1	–0.8	
			+5.4			+1.8			+1	
3D, spheroids (21% O_2_)	0	13.4	–1.6	3.4	25.2	–3.5	3.0	35.8	–5.0	3.0
			+1.8			+4.0			+6.1	
	13.1	4.0	–0.5		8.5	–0.9		11.9	–1.2	
			+0.5			+1.0			+1.3	

a PI = photoindex, defined as EC_50,dark_/EC_50,light_.

b Irradiation conditions: normoxia 520 nm, 10.9 mW cm^–2^, 13.1 J cm^–2^, 20 min; hypoxia 520 nm, 7.22 mW cm^–2^, 13.1 J cm^–2^, 30 min.

c Cancer cells were treated for 24 h in the dark and were not washed before or after irradiation.

**Figure 3:**
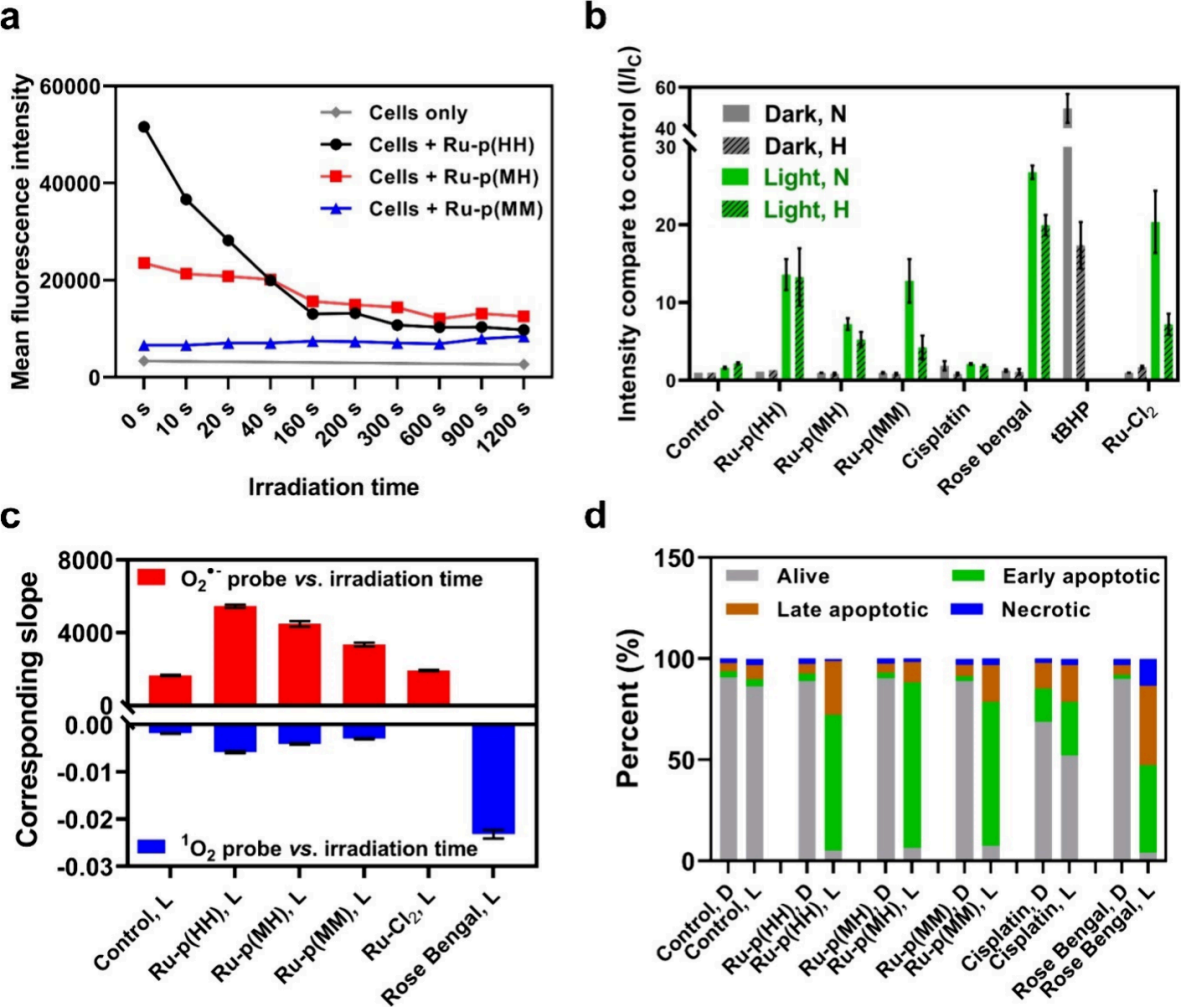
Light activation mechanism in cells treated with Ru-peptide conjugates. (a) Emission intensity of cells treated with **Ru-p(HH)**, **Ru-p(MH)**, or **Ru-p(MM)** (15 μM, 24 h) and irradiated with green light (520 nm, 13.1 J cm^–2^) for different times: 0–1200 s, as determined by flow cytometry using a PC5.5 filter (ex/em = 488/650 ± 50 nm). (b) Intracellular ROS generation assay in normoxic (N) and hypoxic (H) U87MG cells according to FACS analysis using nonselective ROS probe CellROX Deep Red Reagent, after treatment with **Ru-p(HH)**, **Ru-p(MH)**, **Ru-p(MM)**, cisplatin, Rose Bengal, or **[Ru(Ph**_**2**_**phen)**_**2**_**Cl**_**2**_**]** (labeled as **Ru-Cl**_**2**_) (15 μM, 24 h), in the dark and after light irradiation (515 nm, 13.1 J cm^–2^). tBHP (250 μM, 1 h) was used as a positive control for ROS generation. Error bars: SEM, *n* = 2. (c) Slopes of the UV–vis absorption of AMDA (for ^1^O_2_, blue bars) or emission intensity of DHE (for O_2_^•–^, red bars) vs irradiation time (see Figures S26–S28) after being incubated with **Ru-p(HH)**, **Ru-p(MH)**, **Ru-p(MM)**, **Ru-Cl**_**2**_, or Rose Bengal (20 μM) and irradiated with green light in Opti-MEM solution. (d) Percentage of alive (−/−), early apoptotic (−/+), late apoptotic (+/+) and necrotic (+/−) U87MG cells costained with Apopxin Deep Red Indicator and Nuclear Green DCS1 as quantified by flow cytometry, after treatment with **Ru-p(HH)**, **Ru-p(MH)**, **Ru-p(MM)**, cisplatin or Rose Bengal in the dark (D) and after light irradiation (L, DLI = 24 h, 515 nm, 13.1 J cm^–2^); drug concentration: 20 μM, DLI = 24 h).

To better understand the behavior of the complexes in cells, a mechanistic study was conducted *in vitro*. First, to follow the light activation of the compounds, the emission intensity of cells treated with each complex was measured as a function of irradiation time (0–1200 s) using flow cytometry (Figure 3a, Figure S23, ex/em = 488/650 nm). Before light irradiation, the strongest red emission was detected for **Ru-p(HH)**. An intermediate emission was observed for **Ru-p(MH)**, while cells treated with **Ru-p(MM)** were barely emissive. This trend corresponds well with the relative phosphorescence intensities shown in Figure 1b. Upon increasing the green light activation times in cells from 0 to 20 min, the emission from **Ru-p(HH)** and **Ru-p(MH)** decreased gradually, which we interpret as a consequence of ligand photosubstitution. Since the emission intensity of cells treated with **Ru-p(MM)** in the dark was very weak, it was impossible to detect the evolution of the emission, but considering the highest photosubstitution quantum yields of this compound among the three complexes (Table 1), we hypothesize that ligand cleavage took place for this complex within these same 20 min of light irradiation.

Second, CellROX Deep Red, a nonspecific molecular probe for ROS formation, was used to quantify the photodynamic behaviors by each of the three complexes in U87MG cells,.^22^ This study was conducted in the dark or following green light irradiation (515 nm, 13.1 J cm^–2^) under normoxic or hypoxic conditions (Figure 3b, Figures S24 and S25). As expected, the control PDT type II agent Rose Bengal produced a high amount of ROS, while the chemotherapeutic drug cisplatin barely showed any ROS formation. Unexpectedly, however, the three ruthenium-peptide conjugates showed notable ROS production in the light group under normoxia, including **Ru-p(MM)**. Under hypoxia, the ability of **Ru-p(HH)** to generate ROS was retained, while that of **Ru-p(MM)** almost disappeared. As mentioned above, photochemically speaking **Ru-p(MM)** shows the typical characteristics of a PACT compound, i.e., high rates of photosubstitution, poor emission before light activation, and a low ^1^O_2_ generation quantum yield. Thus, it may seem surprising to observe ROS production after green light activation of this compound in cells. Interestingly, however, the precursor of the bis-aqua product of photosubstitution, i.e., **[Ru(Ph**_**2**_**phen)**_**2**_**Cl**_**2**_**]**, followed a similar behavior: upon green light irradiation, it was able to generate ROS in cells. To explain both observations, we suspected that upon light activation of **Ru-p(MM)**, the peptide Ac-MRGDM-NH_2_ was first dissociated following the PACT mechanism, and the resulting activated ruthenium photoproduct [Ru(Ph_2_phen)_2_(OH_2_)_2_]^2+^ was able to bind to either proteins or DNA to form a ruthenium-biomolecule adduct, which is capable of absorbing light and further generating ROS. The good phosphorescence and ^1^O_2_ generation of **Ru-p(HH)** indeed suggests that once bound to histidine ligands, this particular Ru warhead has excellent photodynamic properties. **[Ru(Ph_2_phen)_2_Cl_2_]** may result in the formation of similar adducts by thermal hydrolysis of the chloride ligands and binding to histidine from the cellular environment.^46^ From this ROS generation study, it appeared clearer that the detection or absence of intracellular ROS cannot be used as single diagnostic for a PDT vs PACT mode of action, as activation of a PACT molecule such as **Ru-p(MM)** by photosubstitution inside a cell may lead to the formation of a photoproduct that may show good photodynamic properties.

To further distinguish what kind of ROS these compounds were generating, two more selective ROS probes were used in Opti-MEM medium in the presence of the different ruthenium compounds and light. First, chemoselective ^1^O_2_ detection was achieved by using 9,10-anthracenediyl-bis(methylene)dimalonic acid (AMDA, see Figures S26 and S28a). As shown in Figure 3c, ^1^O_2_ production according to this probe followed the order **Ru-p(HH)** > **Ru-p(MH)** > **Ru-p(MM)**. This trend fits well with the ^1^O_2_ generation quantum yields directly detected by NIR spectroscopy in CD_3_OD. However, compared to Rose Bengal, their capability of generating ^1^O_2_ was much lower. To characterize a possible PDT type I mechanism, the generation of superoxide radicals (O_2_^•–^), which can further evolve into secondary ROS such as HO^•^ or H_2_O_2_,^47^ was also measured using the dihydroethidium (DHE) molecular probe (Figures S27 and S28b).^48^ Interestingly, for all three Ru-peptide conjugates, efficient O_2_^•–^ formation was observed. Here as well, the amount of radical generated decreased following the order **Ru-p(HH)** > **Ru-p(MH)** > **Ru-p(MM)**. These results demonstrated that **Ru-p(HH)** is capable of photochemically generating both ^1^O_2_ and O_2_^•–^ with the highest efficiency, while the product of photosubstitution of **Ru-p(MH)** and **Ru-p(MM)** generates essentially O_2_^•–^ radicals.

In living cells, ROS formation is deleterious and often followed by cell death. In order to study what kind of cell death took place with these compounds, an Apopxin Deep Red Indicator/Nuclear Green DCS1 double staining experiment was performed and analyzed by flow cytometry. Cisplatin and Rose Bengal were also included in the study as a comparison to chemotherapy and PDT type II (Figure 3d and Figure S29). A significantly higher number of dead cells were detected in all three ruthenium-treated, irradiated light groups. In addition, cells mainly died via early and late apoptosis. In conclusion, although **Ru-p(HH)**, **Ru-p(MH)**, and **Ru-p(MM)** seem to show different primary activation pathways as photoactivated agents, efficient light-induced apoptosis was observed for all compounds *in vitro*.

### 2.5 Anticancer Study on 3D Multicellular Spheroids

**Figure 4:**
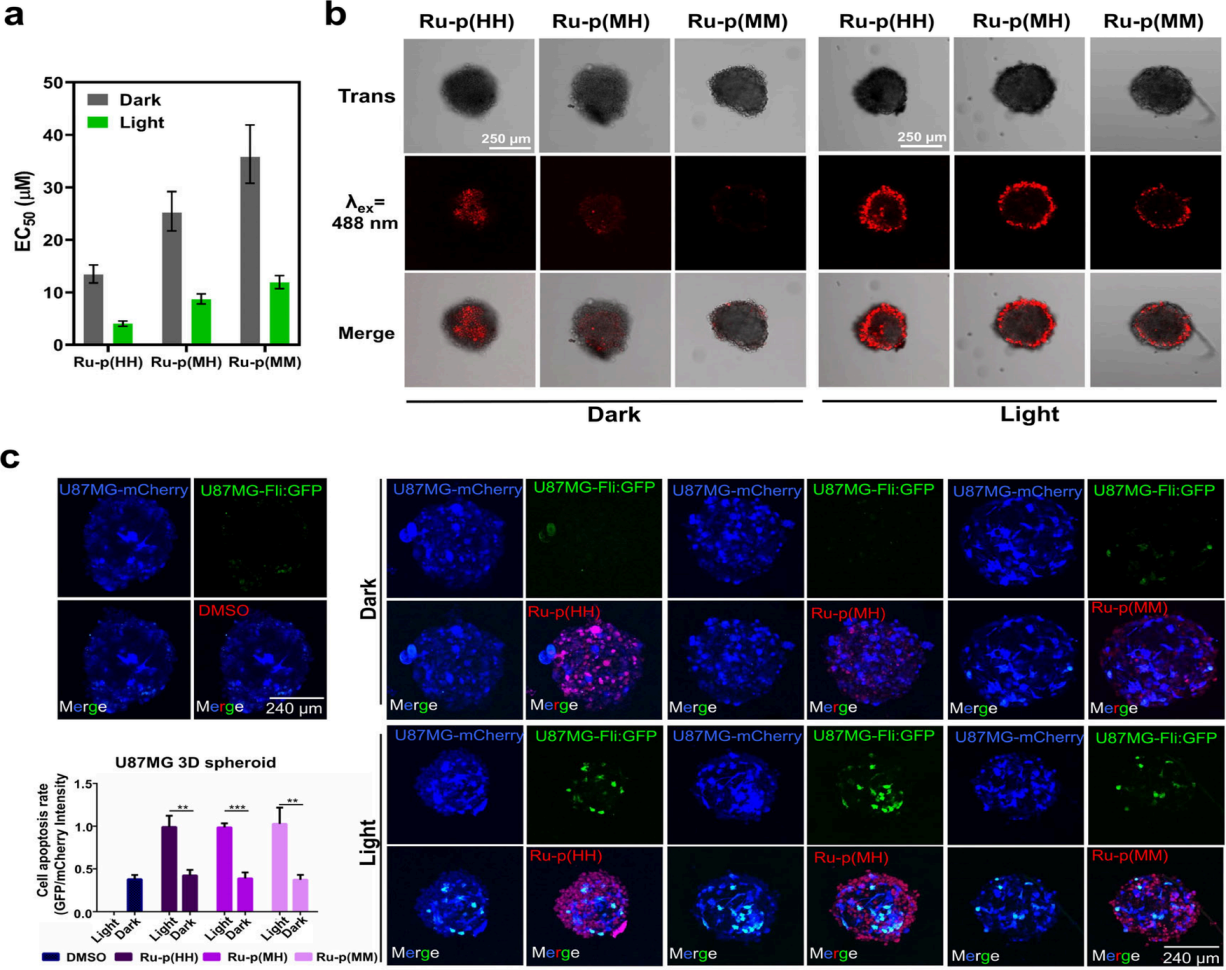
Antitumor effect of Ru-peptide conjugates on U87MG 3D spheroids. (a) EC_50_ of **Ru-p(HH)**, **Ru-p(MH)**, and **Ru-p(MM)** on 3D U87MG spheroids (see values in Table 2). (b) Confocal images of 3D U87MG spheroids treated with **Ru-p(HH)**, **Ru-p(MH)**, and **Ru-p(MM)** in the dark (12 μM) or activated by green light (515 nm, 13.1 J cm^–2^) and then further cultured in the dark for 2 days. In the images, the drug was excited at 488 nm, and its emission was detected at 683–774 nm. Scale bar: 250 μm. (c) Confocal images of 3D U87MG cells stably expressing Flip:GFP-T2A-mCherry spheroids treated with **Ru-p(HH)**, **Ru-p(MH)**, and **Ru-p(MM)** (12 μM) in the dark or after green light activation (515 nm, 13.1 J cm^–2^) for 2 days. U87-mCherry (blue) indicates the basic expression level of protein. U87-Flip:GFP indicates the level of cell apoptosis. Red emission represents **Ru-p(HH)**, **Ru-p(MH)**, or **Ru-p(MM)**. Scale bar: 240 μm. The fluorescence intensity of GFP and mCherry were used to indicate the apoptosis rate of cells. The higher the ratio, the stronger the apoptosis of cells. Statistics were collected from three independent replicates. Two-way ANOVA was used to determine the significance of the comparisons of data (**P < 0.01, ***P < 0.001).

Compared to 2D cancer cell monolayers, 3D multicellular tumor spheroids can provide a more accurate model for the physical penetration of drugs, light, and dioxygen inside a real tumor.^49,50^ The cytotoxicity of **Ru-p(HH)**, **Ru-p(MH)**, and **Ru-p(MM)** was hence further measured using a CellTiter-Glo 3D end point ATP quantification assay (Figure S30).^51^ Phase-contrast bright-field imaging microscopy was used to follow the morphology of the spheroids (Figure S31). Similar to the case for 2D, a good photoactivated toxicity was observed in the 3D environment for all three complexes (Table 2 and Figure 4a). Notably, the EC_50,dark_ of **Ru-p(HH)** and **Ru-p(MH)** on U87MG spheroids was even lower than that in 2D, suggesting a good penetration of these complexes. The EC_50,light_ of the two complexes in 3D seems to stand between those in the normoxic and hypoxic 2D cell models. For **Ru-p(MM)**, higher EC_50_ values under both dark and light conditions were observed in 3D compared to 2D cell cultures, leading to a similar PI value compared with those of the two other conjugates. In addition, a gradually decreasing spheroid size was observed with an increasing concentration of the complexes in the light group (Figure S31).

A follow-up confocal study was conducted after the spheroids were treated with the three conjugates before/after light activation (Figure 4b). For **Ru-p(HH)**, a bright red emission in the spheroids was observed before light activation, suggesting efficient penetration of this prodrug. This signal was weaker for **Ru-p(MH)** and negligible for **Ru-p(MM)**, which follows their respective emission properties (Figure 1b, Table 1). Interestingly, when the spheroids were imaged again 2 days after light activation, a much stronger emission was detected for all three compounds, i.e., also for **Ru-p(MH)** and **Ru-p(MM)**, although they were poorly visible or not visible before light activation. This observation suggested a working hypothesis for the ROS generation of light-activated **Ru-p(MH)** and **Ru-p(MM)** in cells reported in Figure 3b. After irradiation, the peptide (either Ac-MRHDH-NH_2_ or Ac-MRGDM-NH_2_) was released, leaving the primary Ru photoproduct [Ru(Ph_2_phen)_2_(X)(X′)]^2+^ (X, X′ = OH_2_ or other weakly bound biological ligands) free to further react with proteins (or DNA), leading to secondary products with a first coordination sphere that resembles that of **Ru-p(HH)**. Such secondary photoproducts would hence both emit the red light we see by microscopy and generate ROS upon further light irradiation, as described in the ROS generation study.

To understand the effect of light activation in the 3D spheroid context, an apoptosis study was conducted using the U87MG cell line expressing a cellular apoptosis-reporting system: Flip:GFP-T2A-mCherry.^52^ In nonapoptotic cells, only mCherry fluorescence is emitted. When cells undergo apoptosis, the activated cleaved-caspase 3 cleaves and modifies Flip:GFP to emit green fluorescence. In spheroids treated with **Ru-p(HH)**, **Ru-p(MH)**, or **Ru-p(MM)** (12 μM), an apoptotic signature (GFP signal) was observed in the light group 48 h after light activation that was much stronger than in the dark treated or nontreated vehicle control groups (Figure 4c). Thus, light activation of all three ruthenium-peptide conjugates also induced apoptosis in large 3D tumor spheroids (the volume was about 1.4 × 10^–7^ mm^3^), which is in line with the apoptotic results in 2D U87MG cell monolayers.

### 2.6 Targeting of **Ru-p(HH)** to U87MG Tumors in Zebrafish Model

The excellent anticancer properties of the ruthenium-peptide conjugates both in the 2D and 3D *in vitro* assays encouraged us to further evaluate **Ru-p(HH)**, **Ru-p(MH)**, and **Ru-p(MM)***in vivo* using a zebrafish embryonic U87MG xenograft tumor model. First, we examined the biodistribution of the most emissive of the conjugates, **Ru-p(HH)**, in zebrafish embryos without tumor cells. To do so, we injected intravenously (IV) 1 nL of the compound at a concentration of 2 mM (2 pmol in total) in to Tg(*fli1*:eGFP)^y1Tg^/Casper zebrafish embryos at 2 days post fertilization (dpf). Four hours after injection, whole-body confocal microscopy images of the zebrafish embryo showed that the drug flowed through the blood vessels of the embryo and distributed clearly in the brain (Figure S32a,b). The fluorescence distribution mountain map showed that **Ru-p(HH)** was mainly distributed in the lumen of blood vessels and did not bind to vascular endothelial cells. The Pearson correlation coefficient (PCC) and the Manders’ overlap coefficient (MOC) values were 0.06 and 0.15, respectively. Thus, **Ru-p(HH)** was not targeted to vascular cells (Figure S32c,d).

**Figure 5:**
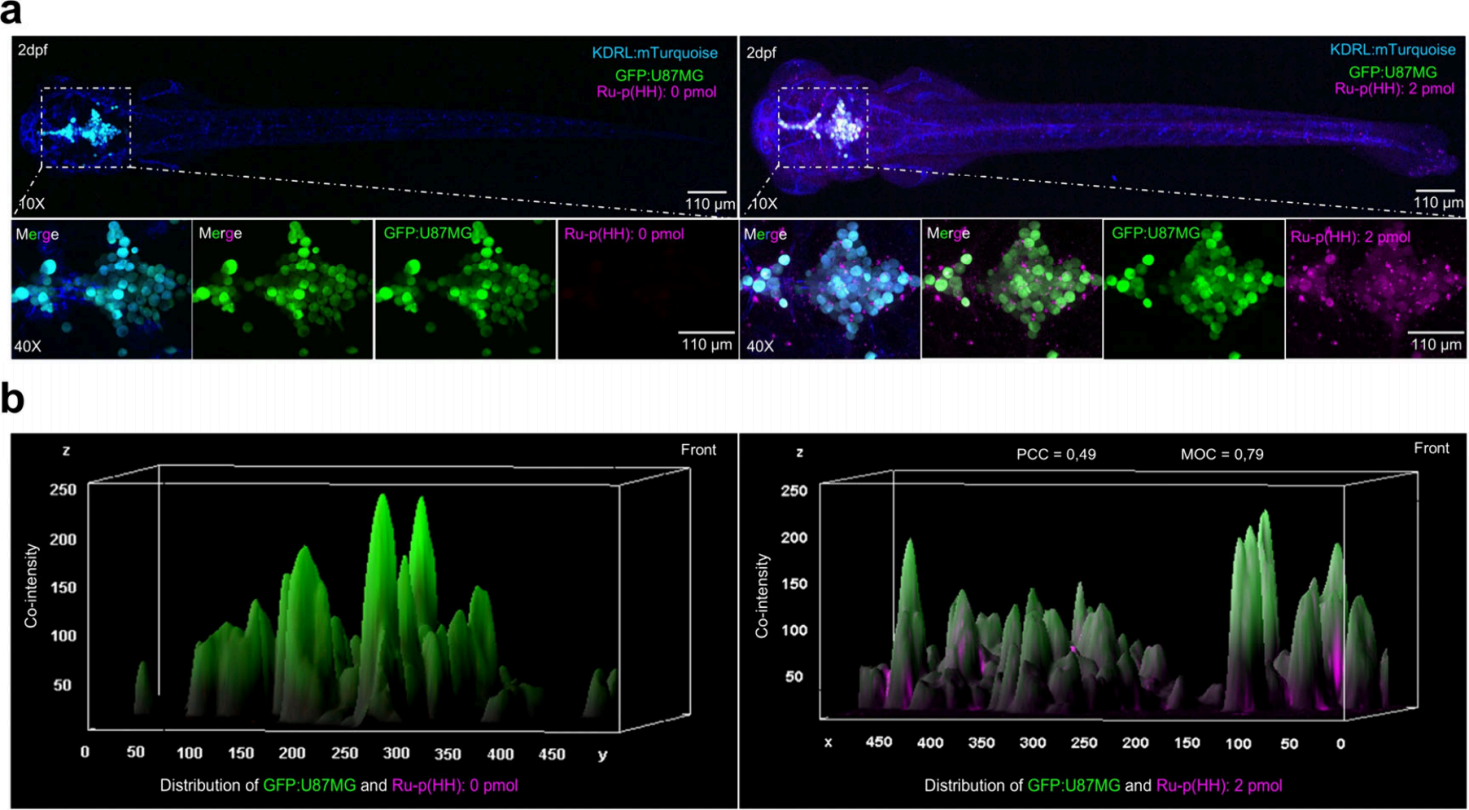
Targeting a U87MG brain tumor with **Ru-p(HH)***in vivo*. (a) Distribution of U87MG cells (green) and **Ru-p(HH)** (purple, dosage: 1 nL × 2 mM = 2 pmol) in the hindbrain of Tg(*kdrl*:mTurquoise) (blue) zebrafish. DMSO was used as a negative control for **Ru-p(HH)**. (b) The mountain map in the lower figure represents the colocalization analysis of **Ru-p(HH)** and U87MG in the top view of the embryo. The *Y*-axis shows the distribution of the drug (purple) on the cells (green); the more overlapping areas of green and purple, the larger the colocalization area. The *Z*-axis is the cell fluorescence intensity (green) and the drug fluorescence intensity (purple). The higher the intensity of purple and green in the same mountain, the stronger the colocalization. Both PCC and MOC are calculated and are shown in the graph. PCC values range from −1 to 1, where −1 represents anticolocalization, 0 represents a random distribution of both colors, and 1 represents complete colocalization. The value range of the MOC is 0 to 1, where 0 represents complete separation and 1 represents complete overlap.

In a second step, the targeting of GBM cells by **Ru-p(HH)** was tested by detecting the colocalization of the red-emissive drug (excitation: 488 nm; emission detector window: 630–774 nm) with GFP-U87MG cells (excitation: 488 nm; emission: 485–575 nm). To clearly see the distribution of the drug and of the cancer cells in zebrafish, we used Tg(*kdrl*:mTurquoise)^hu7185Tg^ embryos. In this transgenic fish line, the blood vessels of the embryos are labeled by the mTurquoise fluorescent protein, which can be excited at 405 nm and emits at 464–480 nm. As shown in Figure 5a and Figure S33a, when only tumor cells were injected into the hindbrain of the zebrafish embryo, GFP was the only signal detected. In the hindbrain of embryos xenografted with tumor cells and treated with **Ru-p(HH)**, the distribution area of the drug (purple) overlapped well with the distribution area of the cancer cells (green). By calculating the distribution and intensity of **Ru-p(HH)** emission in the region of U87MG cells in the brain, the PCC and MOC values for the top-down view of the hindbrain were determined to be 0.49 and 0.79, respectively, while those in the side images were 0.77 and 0.66 (Figure 5b, Figure S33b).

The colocalization study was also conducted for a nontargeted, RGD-free analogue ruthenium complex (Figure S35). The control complex chosen was **[Ru(Ph**_**2**_**phen)**_**2**_**(bpy)]Cl**_**2**_, as it had similar dark cytotoxicity compared with **Ru-p(HH)** toward U87MG in normoxic 2D U87MG cell monolayers (see Figure S34, Table S1), it was as thermally stable as **Ru-p(HH)**, and it showed similar red phosphorescence emission that allowed us to perform bioimaging. High-resolution imaging of the U87MG region within the zebrafish brain revealed that **Ru-p(HH)** exhibited substantial drug accumulation in the brain tumor area. This drug accumulation was notably higher compared with that obtained with the peptide-free prodrug **[Ru(Ph**_**2**_**phen)**_**2**_**(bpy)]Cl**_**2**_ (Figure S35a). Furthermore, the fluorescence area (Figure S35b) and intensity (Figure S35c) corresponding to the drug concentration in the tumor region were much higher for **Ru-p(HH)** than for the control **[Ru(Ph**_**2**_**phen)**_**2**_**(bpy)]Cl**_**2**_. **Ru-p(HH)** thus had a superior ability to target the tumor compared to the RGD-free complex **[Ru(Ph**_**2**_**phen)**_**2**_**(bpy)]Cl**_**2**_. The PCC and MOC were calculated as well to quantify the colocalization between the prodrug and the tumor. With **[Ru(Ph**_**2**_**phen)**_**2**_**(bpy)]Cl**_**2**_, the PCC was −0.2, and the MOC was 0.35, showing moderate correlation. In contrast, for **Ru-p(HH)** the PCC was 0.56, and the MOC was 0.80, showing high correlation. Altogether, these results suggested that **Ru-p(HH)** clearly exhibited colocalization with glioblastoma cells in the zebrafish, while the nontargeted complex **[Ru(Ph**_**2**_**phen)**_**2**_**(bpy)]Cl**_**2**_ did not. Importantly, these results clearly indicated that **Ru-p(HH)** was able to enter the brain cavity and bind to U87MG tumor cells located in the hindbrain region.

### 2.7 Systemic Toxicity

Before testing the antitumor properties of the conjugates in the zebrafish embryo tumor model, the systemic toxicity of **Ru-p(HH)**, **Ru-p(MH)**, and **Ru-p(MM)** to zebrafish embryos was measured. A vehicle control (DMSO) or different amounts of **Ru-p(HH)**, **Ru-p(MH**), or **Ru-p(MM)** were injected into 2 dpf zebrafish embryos, which was repeated 18 h after the first treatment. Stereomicroscopic imaging of the zebrafish embryos at 8 dpf showed that the drug without photoactivation was nontoxic up to a 6 pmol dose, while the drug after green light activation was toxic to zebrafish at 6 pmol, with relatively high mortality and malformation rates (images not shown). At doses of 4 pmol or lower, the zebrafish survived the treatment and developed perfectly well (Figure S36). Overall, 4 pmol seemed to represent the maximal tolerated dose (MTD) of all three compounds in this model.

### 2.8 Antitumor Properties of Photoactivated Ru-Peptide Conjugates in an Orthotopic Zebrafish Brain Cancer Model

Unfortunately, due to the low emission properties of **Ru-p(MH)** and **Ru-p(MM)**, it was impossible to realize with these two derivatives similar biodistribution experiments as with **Ru-p(HH)**. However, considering their similar formulas and *in vitro* targeting properties, we hypothesized that all three compounds probably targeted the tumor in a similar fashion. To justify this hypothesis, we examined the antitumor effect of the three different prodrugs in an orthotopic zebrafish brain tumor model without mature BBB. To realize such an experiment, the growth curves of U87MG tumors in the zebrafish brain were first determined by seeding U87MG cells to 2 dpf zebrafish and recording tumor cell growth by confocal microscopy at 8 dpf. The confocal images (Figure S37a,b) showed that under these conditions, the injected U87MG cells proliferated and formed tumors in the hindbrain, thus providing an orthotopic brain tumor model with a sufficient therapeutic window for antitumor efficacy testing of Ru-peptide conjugates.

**Figure 6:**
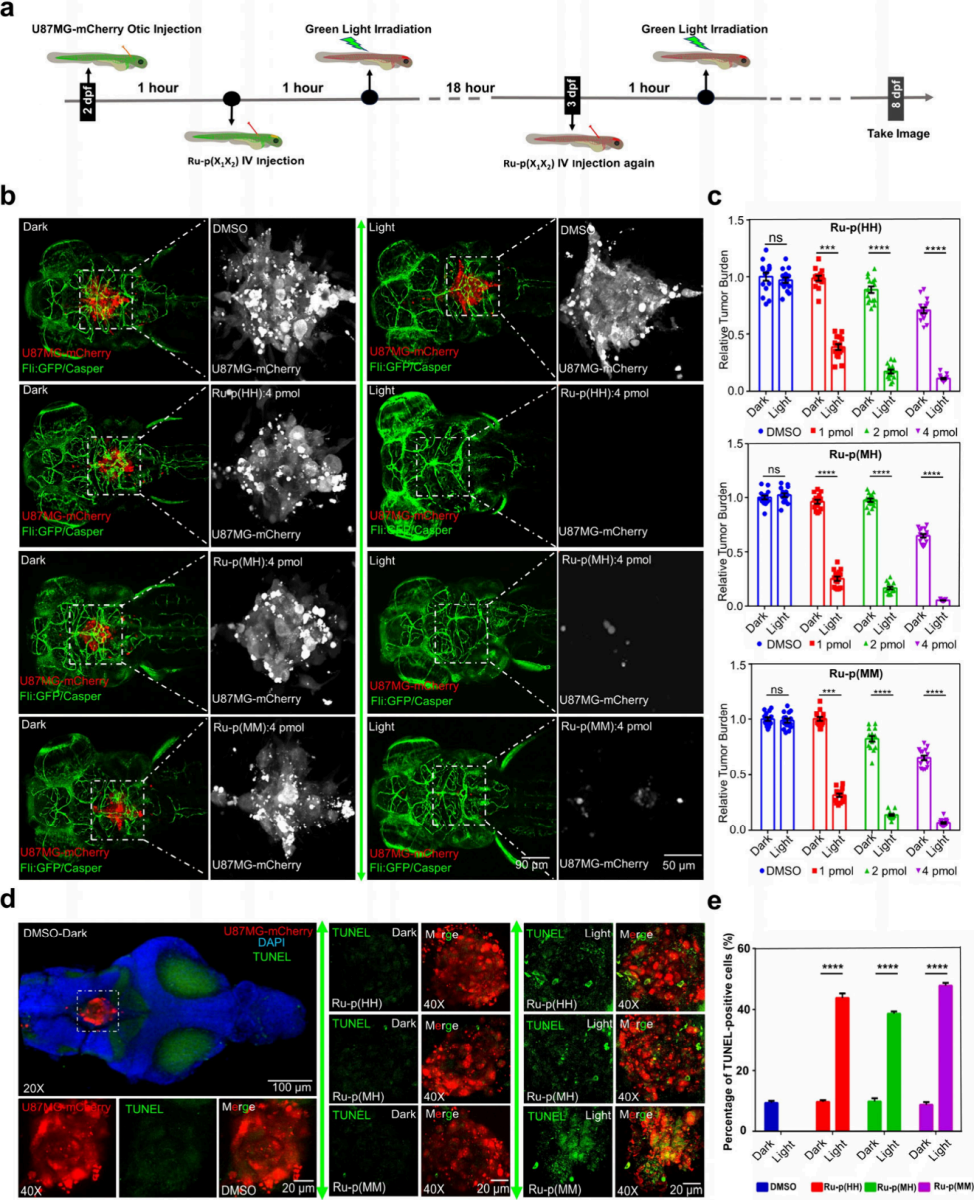
Antitumor effect of Ru-peptide conjugates in an orthotopic zebrafish embryo U87MG xenograft tumor model. (a) Timeline of the antitumor activity experiment. (b) Confocal images of mCherry-labeled U87MG xenografts showing the tumor burden in red upon treatment with a vehicle control (DMSO), **Ru-p(HH)**, **Ru-p(MH)**, or **Ru-p(MM)** (Ru dose: 1 nL, 4 mM, 4 pmol) in the hindbrain of Tg(Fli:GFP/Casper) zebrafish embryos at 8 dpf (dark = drug-treated zebrafish embryos not exposed to light; light = drug-treated embryos after green light activation (520 nm, 78.5 J cm^–2^). Green represents the vasculature of the zebrafish embryos; Casper is a melanin-deficient fish lineage. (c) The relative intensity of red fluorescence (the ratio of the mean values between all groups and the vehicle dark group, respectively) was used to measure tumor burden at the hindbrain. *N* = 15 fishes were used in each group for statistics. Data were generated by ImageJ and analyzed by GraphPad Prism. (d) The image of isolated 4 dpf Tg(Casper) zebrafish brain engrafted with U87MG-mCherry, which was treated by 2 pmol of **Ru-p(HH)**, **Ru-p(MH)**, or **Ru-p(MM)** (in red) at 2 dpf; 3 brains per group were stained by *in situ* TUNEL assay (cell death = green emission). DAPI was used to stain the nuclei. (e) The percentage of TUNEL-positive cells was calculated using the ratio of green fluorescence intensity (apoptosis) to red fluorescence intensity (tumor burden). Error represents standard deviation (SD) from duplicate independent experiments. Unpaired *t* test was used to determine the significance of the comparisons of data indicated in (d) and (e) (***P < 0.001, ****P < 0.0001).

Using such an orthotopic U87MG xenograft tumor model, we examined in a second step the antitumor effects of **Ru-p(HH)**, **Ru-p(MH)**, and **Ru-p(MM)** in the dark and upon green light activation. Zebrafish embryos were implanted with U87MG cells and subsequently treated with DMSO, as well as 1 pmol of one of the three different drugs. At 8 dpf, the tumor size was determined by a confocal microscope (Figure 6a). The U87MG-mCherry tumor fluorescence signal was essentially unchanged in zebrafish treated with the vehicle control (DMSO) with or without light activation. In the groups that received 1 pmol of **Ru-p(HH)**, **Ru-p(MH)**, or **Ru-p(MM)**, green light activation (520 nm, 78.5 J cm^–2^) mostly cleared the tumor burden, compared to the dark group, but a small amount of residual cancer cells did remain. When the prodrug dose was increased to 2 or 4 pmol, the tumor cells in the zebrafish brain were almost completely cleared (Figures S39 and S40). All prodrugs were essentially unable to induce tumor cell death without light activation (Figure 6b). A statistical analysis of the tumor growth in each group (*N* = 15 zebrafish) showed that the prodrugs after illumination had a significantly stronger inhibition of tumor growth than those not activated by illumination (Figure 6c), demonstrating that the antitumor efficacy of **Ru-p(HH)**, **Ru-p(MH)**, and **Ru-p(MM)** was activated by green light, proving that **Ru-p(HH)**, **Ru-p(MH)**, and **Ru-p(MM)** possess both targeting and photoactivation properties to attenuate U87MG tumor cells in this model. As noted, the relatively similar antitumor efficacy of all three compounds *in vivo* is noticeable considering the different *in vitro* properties of these compounds. We hypothesize that such similarity is a consequence of three different facts. First, all three ruthenium-peptide conjugates have similar targeting properties. Second, tumor sizes in zebrafish embryo models are small, and under such conditions, tumors are not hypoxic, leading to similar anticancer properties for the three compounds (see the similar normoxic photoindex values in Table 2). Finally, we are in a special case here of a PACT compound (**Ru-p(MM)**) that, following the photochemical release of the MRGDM peptide and binding to proteins via two histidine residues, becomes a secondary photoproduct with good photodynamic properties. These PDT properties of the secondary photoproduct may diminish the differences in biological efficacy between the *a priori* mostly PACT compound **Ru-p(MM)** and the PDT compounds **Ru-p(HH)** and **Ru-p(MH)**.

To further explore the cell-killing mechanism of the three light-activated drugs on U87MG tumor cells located in the hindbrain, zebrafish brains were isolated^53^ from 4 dpf embryos, and dying tumor cells in the brain tissue were detected by an *in situ* TUNEL cell death assay. High-resolution confocal microscopy images of the brains demonstrated that zebrafish treated with the vehicle control hardly showed any cell death and that the ones treated with one of the three Ru-peptide conjugates **Ru-p(HH)**, **Ru-p(MH)**, or **Ru-p(MM)** (2 pmol) and left in the dark showed a comparably lower number of dying cells in the tumor. However, after the prodrug was photoactivated, the brain tumors showed a much stronger level of dying cells, confirming that light activation of the prodrugs endowed killing of the engrafted U87MG cells. Quantification of the percentage of TUNEL-positive cells in the different groups confirmed that the difference between the dark and light-activated groups was statistically significant (Figure 6d,e). Overall, the photoactivation of **Ru-p(HH)**, **Ru-p(MH)**, and **Ru-p(MM)** by whole-body green light irradiation generated a strong antitumor effect induced by tumor cell death.

### 2.9 Do the Ruthenium-Peptide Conjugates Cross the Blood–Brain Barrier in the Zebrafish Embryo Tumor Model?

**Figure 7:**
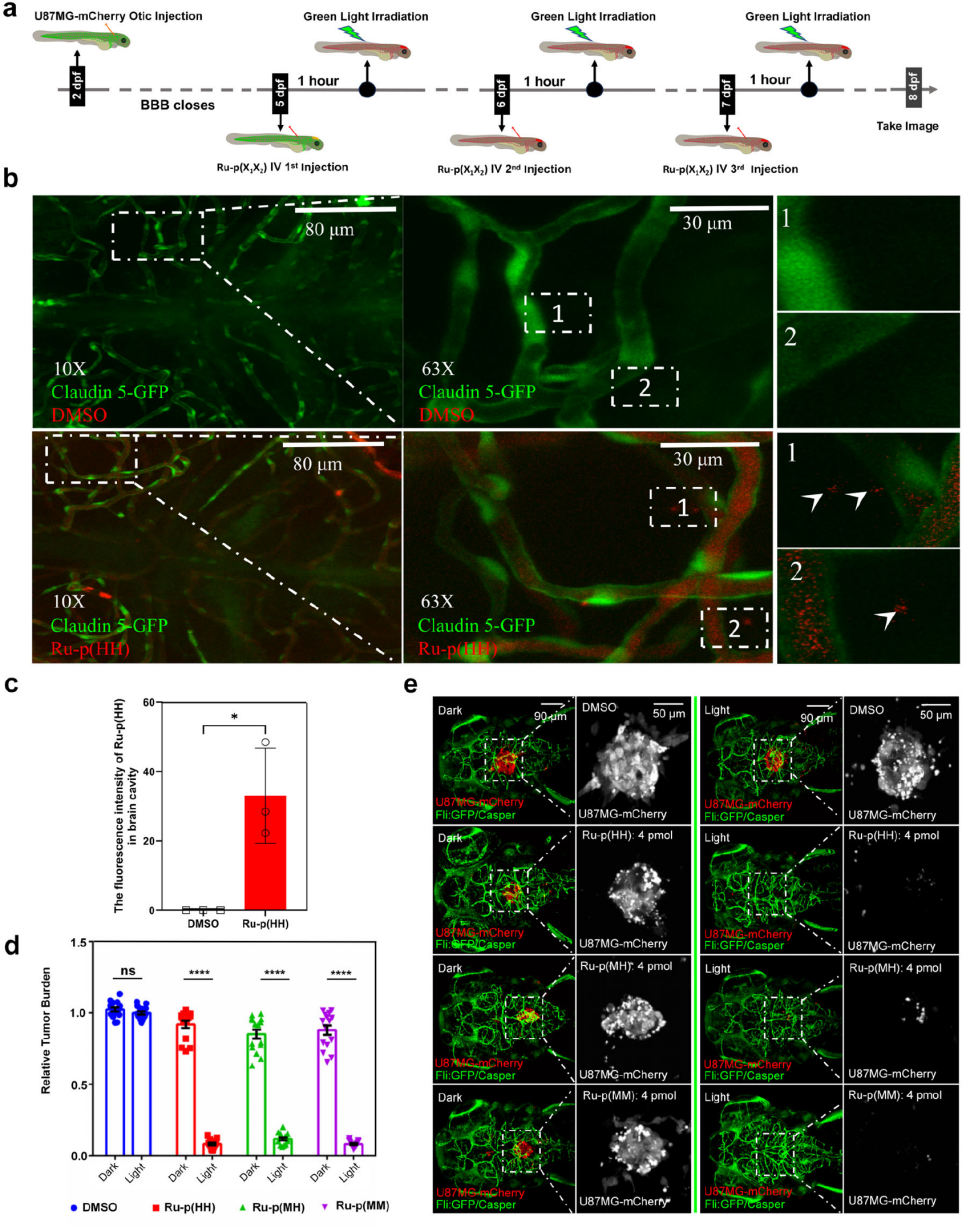
Drug distribution and antitumor effect of Ru-peptide conjugates in an orthotopic zebrafish embryo U87MG xenograft tumor model with a mature blood–brain barrier. (a) Timeline of the antitumor experiment. (b) The brain distribution of intravenously administered **Ru-p(HH)** (dosage: 1 nL × 4 mM = 4 pmol, λ_ex_/λ_em_ = 488/620–680 nm) in 5 dpf zebrafish (the BBB marker Claudin 5 was labeled by GFP) analyzed by high-resolution confocal microscopy. The arrow points to **Ru-p(HH)**, which passes through the BBB into the brain cavity. (c) NIR emission intensity of **Ru-p(HH)** in the brain cavity at 5 dpf. *N* = 3 fish in each group. (d) The relative intensity of mCherry at 8 dpf for U87MG-mCherry tumor burden following three treatments at 5, 6, and 7 dpf with **Ru-p(HH)**, **Ru-p(MH)**, and **Ru-p(MM)** (dosage: 1 nL × 4 mM = 4 pmol) in the hindbrain (dark = drug-treated zebrafish embryos not exposed to light; light = drug-treated zebrafish embryos irradiated with green light (520 nm, 78.5 J cm^–2^)). *N* = 15 fish were used in each group for statistical relevance. Error bars represent standard deviations (SD) from duplicate independent experiments. (e) Representative confocal images at 8 dpf for U87MG-mCherry tumor burden following treatments with **Ru-p(HH)**, **Ru-p(MH)**, and **Ru-p(MM)** reported in (d). Green shows the blood vessels. Unpaired *t* test was used to determine the significance of the comparisons of data indicated in (c) and (d) (****P < 0.0001).

One of the limitations in the treatment of brain tumors in patients is the existence of the BBB,^5^ which strongly hampers the penetration of most antitumor drugs into the brain.^5,6^ In the 2 dpf zebrafish embryos used above, the BBB was not completely mature upon injection of the prodrug, which may be the reason why the prodrugs could penetrate into the brain. To validate if the ruthenopeptide conjugates were able to cross a working BBB and achieve the same antitumor effect, we repeated drug treatments in older zebrafish embryos with a functional BBB. It has been reported that the BBB of zebrafish embryos is largely developed at 3 dpf;^8^ therefore, we engrafted U87MG-mCherry cells into the hindbrain of 2 dpf Fli:GFP/Casper zebrafish embryos and cultured the embryos until 5 dpf. Next, **Ru-p(HH)**, **Ru-p(MH)**, or **Ru-p(MM)** was intravenously injected (1 nL, 4 pmol), and 1 h later, the embryos in the light group were irradiated by green light for 2 h (520 nm, 78.5 J cm^–2^). The treatment was repeated twice at 6 and 7 dpf. Finally, at 8 dpf the tumor burden in each group was recorded as described above using high-resolution confocal microscopy (Figure 7a).

For **Ru-p(HH)**, high-resolution microscopy images were also recorded 4 h after drug injection to look at the prodrug distribution in the 5 dpf embryos. According to these images, the prodrug is distributed not only in the blood vessels, which are superimposed in yellow color, but also in the brain cavities without blood vessels, which are shown as the red color of the drug (Figure S41a). In order to confirm the ability of the prodrug to penetrate the BBB, we used a zebrafish with GFP-labeled Claudin 5, which is the marker of BBB. In other terms, the blood vessels expressing this protein have a functional BBB structure. The prodrug was injected into the blood vessels of the zebrafish at 5 dpf, hence after the BBB had closed.^54−56^ A local zoomed-in view of the blood vessel showed that the prodrug had penetrated the GFP-labeled Claudin 5 vessel into the brain cavity region (Figure 7b). This means that the prodrug penetrated the functional BBB. Quantitative analysis of the far-red fluorescence intensity in the brain cavity of the vehicle control (DMSO) and drug-treated (**Ru-p(HH)**) groups showed that the NIR fluorescence intensity in the brain of the drug-treated zebrafish embryo was significantly higher than that in the brain of the vehicle control group, which was essentially nonemissive (Figure S41b, Figure 7c). According to these data, 4 h postintravenous injection in a 5 dpf zebrafish embryo, **Ru-p(HH)** was able to cross the mature BBB and diffuse into the brain cavity. The antitumor effect of the three drugs was further studied in zebrafish embryos carrying mature BBB. The confocal microscopy images recorded at 8 dpf confirmed that groups treated with **Ru-p(HH)**, **Ru-p(MH)**, and **Ru-p(MM)** and light had a significantly lower tumor burden than the groups treated with prodrug but left in the dark or the vehicle control. Importantly, the tumor burden in the treated groups was basically eliminated after light activation (Figure 7d,e). These data form strong evidence that even after the BBB is formed, the three prodrugs can still reach the brain cavity and that light activation of the ruthenium-peptide conjugated prodrugs effectively kill the tumor cells located in the brain. These exquisite results provide encouraging evidence for the testing of these prodrugs in orthotopic mice models of brain cancer and potentially for the treatment of human brain tumors.

## 3 Discussion and Conclusions

*In vitro*, in Ru-peptide conjugates the replacement of histidine by methionine coordinating residues in the X_1_RGDX_2_ peptides did not significantly change the integrin targeting properties already observed for **Ru-p(MH)**, but they did transform a compound that essentially behaved as a PDT sensitizer (**Ru-p(HH)**) into a compound (**Ru-p(MM)**) that, chemically speaking, behaves essentially as a PACT prodrug with low emission, ^1^O_2_ generation quantum yields, and higher photosubstitution quantum yields. **Ru-p(MH)** lies somewhere in the middle by combining both types of photochemistry (Figure S42). In biological conditions, however, the boundaries between PDT and PACT were less clear-cut. **Ru-p(MM)** kept a significant photoindex value under hypoxia, which makes it a PACT agent, but its secondary photoproducts were found to be emissive and capable of generating ROS, which are characteristics of PDT compounds. Though photosubstitution followed by binding to biomolecules and an absence of ROS are, in principle, typical characteristics for PACT compounds, while ROS generation by a photostable and emissive compound with low-lying ^3^MLCT excited states are typical characters for a PDT compound, in normoxic conditions we found that both mechanisms contributed to the phototoxicity observed for all three ruthenium-peptide conjugates. Under hypoxic conditions, only the first one remained operative, which we tentatively interpret as a consequence of the scarcity of O_2_ molecules to be activated in irradiated hypoxic cells. Consequently, **Ru-p(HH)** lost all of its phototoxicity (PI = 1.3 vs 12.1), and **Ru-p(MH)** lost most of it (PI = 1.9 vs 11.9). Conversely, **Ru-p(MM)** kept a significant phototoxicity (PI = 4.0 vs 8.5).

*In vivo*, excellent tumor targeting and green light-activated antitumor efficacy were observed for all three compounds in zebrafish embryo U87MG xenografts, whether or not the embryo had a functional BBB. We attribute the similar antitumor efficacy of **Ru-p(HH)**, **Ru-p(MH)**, and **Ru-p(MM)** as a consequence of the limited size of the tumors (∼0.0055 mm^3^), which are probably not prone to show important hypoxic areas. Some of us recently published the convincing antitumor efficacy of **Ru-p(MH)** in a subcutaneous mice tumor model, where there is no BBB to cross but where the tumors are much larger (up to 100–150 mm^3^).^36,57^ Though it is uncertain at this stage whether such sizes entail hypoxic areas, they do suggest that the encouraging results obtained in orthotopic zebrafish brain tumor models shown here are not a consequence of the small size of the tumor and that larger tumors can be addressed, too. In terms of tumor targeting, the red-emissive compound **Ru-p(HH)**, when injected intravenously in BBB mature zebrafish embryos, was clearly observed inside the brain tumor area and did not target the endothelium much. The ability of cyclic ruthenium-peptide conjugates to cross the BBB and destroy U87MG tumors efficiently is a rare property. If confirmed in larger tumors of orthotopic brain tumor mice models, such compounds may open a promising route toward efficient brain tumor phototherapy also in the presence of larger hypoxic areas while keeping low systemic toxicity, which may ultimately lead to low side effects for patients.

## 4 Methods

### 4.1 Photosubstitution

The photosubstitution process was monitored by a UV–vis spectrophotometer (Cary 60, Varian) equipped with a temperature control set to 25 °C and a magnetic stirrer. The complex was dissolved in Milli-Q water (25 μM) in a 1 cm optical pathway quartz cuvette containing 3 mL of solution. A cooled 515 nm LED (photon flux = 1.77 × 10^8^ photons cm^–2^ s^–1^) was used as light source, and light was turned on right after one scan. The standard measurement method was a follows: a spectrum measurement (from 800 to 200 nm) was performed every 30 s for 120 min. Photosubstitution quantum yields were determined by Glotaran, as explained in detail by Bahreman and Bonnet.^58^

### 4.2 Determination of ^1^O_2_ Generation Quantum Yields

Singlet oxygen quantum yield measurements were performed by the direct spectroscopic detection of the 1275 nm emission, as described by Meijer et al.^59^

### 4.3 ITGAV Knockdown Cell Line Construction

The pLenti-shITGAV-Puro plasmid (from Sigma’s MISSION library, kindly provided by Department of Molecular Cell Biology, LUMC) and package plasmid (pMD2.G and psPAX) were transfected into HEK-293T cells using lipo-293 transfection reagent to produce shITGAV virus particles. The obtained lentivirus was used to infect U87MG-wt cells and screened with 2 μg/mL puromycin for 1 week.

### 4.4 Integrin Expression Analysis by Flow Cytometry

The double immune-fluorescence method was applied to study the expression of integrins α_V_β_3_ and α_V_β_5_ on the surface of U87MG-kd, U87MG, MDA-MB-231, A549, PC-3, and MCF7 cultured in normoxic (21% O_2_) and hypoxic (1% O_2_) conditions. After thawing, cells were cultured in a 25 cm^2^ flask in either condition for at least 2 weeks; their integrin expression levels were studied according to a reported protocol.^39^ Monoclonal antibodies against human α_V_β_3_ (clone LM609, Merck) or human α_V_β_5_ (ab177004, Abcam) and Alexa-Fluor 488-conjugated goat anti-mouse IgG antibody (Invitrogen, A-11001) were used in this work.

### 4.5 2D Cytotoxicity Assay

U87MG cells (6000) were seeded in 96-well plates (Sarstedt, 83.3924), and each well contained 100 μL of Opti-MEM (Gibco complete medium 11058-021, supplemented with 2.5% v/v fetal calf serum (FCS), 0.2% v/v penicillin/streptomycin (P/S), and 1% v/v glutamine). They were then placed either in a normoxic (21% O_2_) or hypoxic (1.0% O_2_) incubator; 24 h later, different concentrations of **Ru-p(HH)**, **Ru-p(MH)**, or **Ru-p(MM)** dissolved in Opti-MEM (100 μL) were added to the wells in triplicate. For each complex, one dark and one light plate were involved. Each plate was further incubated in the dark for 24 h (DLI). After that, one plate was irradiated with green light (520 nm) for 20 min at 37 °C for normoxia (dose = 13.1 J cm^–2^) or 30 min for hypoxia (dose = 13.0 J cm^–2^), while the other plate was kept in the dark. The cells were further incubated for another 2 days in normoxic or hypoxic conditions, respectively. Finally, 100 μL of cold trichloroacetic acid (10% w/v) was added to each well to fix the cells, and all plates were then transferred to a 4 °C refrigerator for 48 h before an SRB cell quantification end point assay was performed.^45^ All experiments were conducted in biologically independent triplicate.

Hypoxic cell models *in vitro*: all cells were incubated and passaged in a dark hypoxic incubator (1% O_2_) for at least 2 weeks before all hypoxia studies. Adding chemicals to the cells had to be performed in air; however, the cell-growing medium was kept in the hypoxia incubator for at least 2 days before the addition to hypoxic cells, and all hypoxic light irradiations were performed inside the hypoxic incubator set at 1.0% O_2_.

### 4.6 Intracellular Florescence Intensity of **Ru-p(HH)**, **Ru-p(MH)**, or **Ru-p(MM)** during light activation

U87MG cells (5 × 10^4^, 1 mL) were seeded into 24-well plates and incubated for 24 h in the dark under normoxia. The cells were then treated with **Ru-p(HH)**, **Ru-p(MH)**, or **Ru-p(MM)** (15 μM). After 24 h of incubation under normoxia, the plate was washed with cold PBS once, and cells were trypsinized, harvested, and washed again with cold PBS before being resuspended in 150 μL of PBS and then transferred to 96-well round-bottom plates (Thermo Scientific, 268200). The cells were divided into 11 groups, and they were irradiated for 0, 10, 20, 40, 160, 200, 300, 600, 900, or 1200 s with green light (520 nm, 13.1 J cm^–2^). Untreated cells were maintained as a control. The levels of intracellular emission intensity were then determined by using a CytoFLEX flow cytometer. Fluorescence measurements were acquired with the PC5.5 (488 nm excitation, 650 ± 50 nm emission) channel, which is in accordance with the excitation/emission wavelengths of the complexes measured in emission spectroscopy (480 nm excitation, 600–800 nm emission). All flow cytometry data were processed by using FlowJo 10 software.

### 4.7 Measurement of Intracellular ROS

The generation of ROS (reactive oxygen species) in U87MG cells was measured using a ROS deep red fluorescence indicator (Abcam, ab186029). U87MG cells (1 × 10^5^, 1 mL) were seeded into 12-well plates and incubated for 24 h in the dark under normoxia. The cells were then treated with **Ru-p(HH)**, **Ru-p(MH)**, **Ru-p(MM)**, cisplatin, Rose Bengal, or **[Ru(Ph**_**2**_**phen)**_**2**_**Cl**_**2**_**]** (15 μM). There were two groups for each drug (dark + light). After 24 h of incubation under normoxia, the plate was washed with cold PBS once, and cells were trypsinized, harvested, and then resuspended in 150 μL of PBS. The cell suspension from the centrifuge tubes was transferred to 96-well round-bottom plates (Thermo Scientific, 268200), and the plates were kept in the dark or irradiated with 520 nm light (dose = 13.1 J cm^–2^). Afterward, the Cellular ROS Deep Red dye was added with 1000× dilution, and cells were further stained for 1 h. The levels of intracellular ROS were then determined using a CytoFLEX flow cytometer using the APC-A (638 nm excitation, 660/10 nm emission) channel. All flow cytometry data were processed using FlowLogic 8.5 software.

### 4.8 Apoptosis Study in 2D

The apoptosis study was measured by the Apopxin/Nuclear Green DCS1 double staining assay (Abcam, ab176750). First, 2 mL aliquots of U87MG cell suspension (3 × 10^5^ cells/well) were seeded in two 6-well plates (Sarstedt, 83.3920) using Opti-MEM complete medium and allowed to incubate for 24 h in the dark in normoxic conditions, after which cells were treated with **Ru-p(HH)**, **Ru-p(MH)**, **Ru-p(MM)**, cisplatin, or Rose Bengal (20 μM, all drug working solutions were prepared from 4 mM of stock in DMSO). After 24 h of incubation, one plate was irradiated with 520 nm light (13.1 J cm^–2^). Then, both plates were allowed to incubate for another 24 h under normoxia. The cells were then trypsinized, collected, and washed with cold PBS twice. The pellets were stained following the manual provided by the supplier. After staining, the cells were detected by flow cytometry (CytoFLEX flow cytometer). Parameters APC (638 nm excitation, 660/10 nm emission) and FITC (488 nm excitation, 525/40 nm emission) were used. All flow cytometry data were processed using FlowJo 10.

### 4.9 Viability Test and Confocal Laser Scanning Microscopy (CLSM) Images of 3D U87MG Tumor Spheroids

U87MG cells (500 cells) were added to a 96-well round-bottom Corning spheroid microplate (catalog CLS4520) and incubated under normoxia for 3 days to generate 3D tumor spheroids (∼500 nm). One dark and one light plate was included in one group. After that, different concentrations of **Ru-p(HH)**, **Ru-p(MH)**, or **Ru-p(MM)** dissolved in Opti-MEM were added to the wells in triplicate. The spheroids were incubated further under normoxia. After 24 h, the light plate was irradiated with green light for 30 min (dose of 13.0 J cm^–2^), and the other plate was left in the dark. The cells were further incubated under normoxia in the dark for 2 days, and finally at *t* = 96 h, a CellTiter-Glo 3D solution (100 μL/well) was added to each well to stain the 3D tumor spheroids. After shaking for 30 min, the luminescence (560 nm) in each well was measured with a Tecan microplate reader. All experiments were conducted in biologically independent triplicate.

The CLSM images of U87MG tumor spheroids were captured with a Leica SP8 microscope, with a 8-Well with Glass Bottom μ-Slide (Ibidi, 80827), The fluorescence intensity profile plots were generated using Fiji ImageJ software. The complexes were excited at 488 nm, and emission was recorded in the 683–774 nm window.

### 4.10 Apoptosis Study in 3D Flip:GFP-T2A-mCherry U87MG Tumor Spheroid Models

The Flip:GFP-T2A-mCherry U87MG cell line was constructed by lentivirus and with the method described in Section 4.3. The 3D Flip:GFP-T2A-mCherry U87MG tumor spheroid experiment was the same as that described above in Section 4.9. CLSM images were captured using a Leica SP8 microscope. Flip:GFP was excited by the 488 nm laser line and was detected at 510 nm; mCherry was excited by the 568 nm laser line and was detected at 610 nm. **Ru-p(HH)**, **Ru-p(MH)**, and **Ru-p(MM)** were excited by the 488 nm laser line and detected at 683–774 nm.

### 4.11 Distribution of **Ru-p(HH)** in Zebrafish Embryos

Zebrafish were handled in compliance with current legislations (license number AVD1060020172410 and AVD10600202216495) and by following standard zebrafish rearing protocols (https://zfin.org), which adhere to the international guidelines from the EU Animal Protection Direction 2010/63/EU. **Ru-p(HH)** (2 mM) in DMSO was injected into 2 dpf anesthetized zebrafish embryos with 0.02% buffered 3-aminobenzoic acid ethyl ester (tricaine; Sigma-Aldrich, A-5040) through the duct of Cuvier by microinjection. Drug-injected zebrafish embryos were incubated in drug-free egg water for 4 h at 33 °C. Zebrafish embryos were again anesthetized and placed in a glass-bottom Petri dish and covered with 1% low-melting agarose containing tricaine. Embryos were imaged using a Leica TCS SP8 confocal microscope with a 40× or 63× oil immersion objective (NA = 1.4), equipped with 488, 532, and 638 nm laser lines. GFP was excited by the 488 nm laser line and detected at 510 nm; **Ru-p(HH)** was excited by the 488 nm laser line and detected at 683–774 nm.

To test targeting, 300–500 U87MG cells stably expressing GFP were injected through the otic vesicle into the hindbrain of 2dpf Tg*(kdrl:*mTurquoise) zebrafish embryos. One hour post tumor injection, a vehicle control (DMSO) or **Ru-p(HH)** (deep red) was IV injected into the embryos. After 4 h, images of whole zebrafish embryos and high-resolution images of the hindbrain were taken from both the top or the side of the animal.

### 4.12 Zebrafish Xenograft Model of U87MG Tumor Cells

About 300 to 500 cells suspended in 2% polyvinylpyrrolidone-40 (Calbiochem, San Diego, California, USA) were injected under a microscope through the otic vesicle of the 2 dpf embryo into the hindbrain using a capillary glass tube. Zebrafish embryos grown with tumor cells were further incubated in fresh egg water in a 33 °C incubator. An SP8 confocal microscope was used to obtain tumor images of the hindbrain and record tumor growth in zebrafish embryos at 3 dpf (days post fertilization), 5 dpf, and 8 dpf, respectively. The fluorescence intensity and size of the grafted tumors were calculated using ImageJ, which were used to calculate the mean fluorescence values (mean = tumor fluorescence intensity/tumor area). The calculated mean values of 3, 5, and 8 dpf tumors were divided by the mean values of 3 dpf tumors for normalization.

### 4.13 Evaluating the Antitumor Effect of Light-Activated **Ru-p(HH)**, **Ru-p(MH)**, and **Ru-p(MM)** in Zebrafish Model

Tumor cells and zebrafish embryos were prepared as described previously. Tumor cells were implanted into the hindbrain of the 2 dpf embryo, and 1 h later, 1 pmol (1 nL, 1 mM), 2 pmol (1 nL, 2 mM), or 4 pmol (1 nL, 4 mM) of **Ru-p(HH)**, **Ru-p(MH)**, and **Ru-p(MM)** (dissolved in DMSO) were injected into each zebrafish embryo by IV administration. Another 1 h later, each group of embryo was divided into two subgroups; one was kept in standard culture conditions (15 fishes in a 34 °C incubator in the dark), and the other (15 fishes) was irradiated for 2 h using 520 nm green light (78.5 J cm^–2^) before being put back in standard culture conditions. Eighteen hours after tumor cell injection, zebrafish embryos were given a second dose of **Ru-p(X**_**1**_**X**_**2**_**)**, and the embryo in the light group was given the same light dose again. Zebrafish were cultured continuously up to 8 dpf in a 34 °C incubator, anesthetized with tricaine, and fixed with 1% low-melting agarose. An SP8 confocal microscope was used to record the fluorescence (excitation: 552 nm; emission: 600–700 nm) of the tumor cells in zebrafish of each group at 8 dpf. ImageJ was used to analyze the fluorescence intensity, and GraphPad Prism was used for data statistics.

### 4.14 Cell Death Detection in Brain of Zebrafish Using TUNEL

U87MG-mCherry cells were implanted into Casper zebrafish embryos (without melanin) according to the method described in Section 4.12, and 4 pmol (4 mM in DMSO, 1 nL) of **Ru-p(X_1_X_2_)** was injected into embryos in accordance with the method described in Section 4.13. After grouping, they were subjected to light irradiation or no irradiation. Then, 48 h after, i.e., at 4 dpf, zebrafish embryos were fixated with 4% PFA for 16 h at 4 °C, and the embryos were washed once with PBS containing 0.1% tween-20 (PBT). Zebrafish embryo brains were microseparated out of larvae, removing all the skin around the brains.^53^ Brains were stained with DAPI diluted 1:500 (storage concentration of 6 mM) for 5 min and for the TUNEL (*In Situ* Cell Death Detection Kit, Fluorescein 11684795910 Roche) assay using the manufacture’s procedure. PBT was used to clean 3 times, 5 min each time. The zebrafish embryo brains were placed on glass-bottom Petri dishes and fixed using 0.7% low-melting agarose. Apoptosis was detected by SP8 confocal microscopy (excitation: 488 nm, emission: 515–565 nm).

### 4.15 Bioimaging of the Drug Penetration through the BBB Test and Antitumor Activity in the Presence of Mature BBB

After the zebrafish embryos had been prepared and cultured for 5 dpf, 4 pmol of **Ru-p(HH)** (4 mM in DMSO, 1 nL) was IV injected into each embryo. Four hours later (DLI), the zebrafish embryos were fixed with 1% low-melting agarose, and the distribution of the drug in the posterior cerebrovascular and posterior brain cavity was imaged by an SP8 confocal microscope (10× magnification, 40× magnification) (**Ru-p(HH)**: λ_ex_ = 488 nm and λ_em_ = 620–680 nm, Fli:GFP: λ_ex_ = 488 nm and λ_em_ = 495–575 nm).

The tumor cells were implanted into zebrafish embryos at 2 dpf, and the culture was continued to 5 dpf (3 dpi), where a mature BBB had formed. Then, 4 pmol (1 nL, 4 mM in DMSO) of **Ru-p(HH)**, **Ru-p(MH)**, or **Ru-p(MM)** was injected into each zebrafish by IV administration, and an hour after the injection, the drug was activated by 520 nm green light (10.9 mW cm^–2^, 78.5 J cm^–2^) for 2 h. The treatment was repeated twice (at 6 and 7 dpf). The zebrafish were fixed at 8 dpf (6 dpi), and the tumor burden in the hindbrain of the zebrafish was quantified base on the tumor burden size and fluorescence intensity by confocal microscopy and analyzed using ImageJ.

### 4.16 Theoretical Modeling

DFT models of the Λ isomer of [Ru(L)_2_(OH_2_)]^2+^ and of the MRGDM, MRGDH, and HRGDH free peptides were built and minimized in the vacuum using ADF 2019 from SCM^60^ at the GGA:BLYP level using scalar relativistic effects for ruthenium, a DZ basis set, and no frozen core. The minimized structures were imported in YASARA Structure.^61^ The aqua ligands were removed, and a bond was introduced between the histidine N or methionine S atoms of the peptide and the Ru center. Ru^2+^ was replaced by Fe^2+^, and the conjugates were minimized using molecular mechanics in YASARA. The models were then reintroduced into ADF. Fe^2+^ was changed back to Ru^2+^, and the complexes were minimized again at the same DFT level. Finally, the geometries were minimized at the PBE0/TZP/COSMO level in water.

## Supporting Information Available

General information and synthesis details; HR-MS, HPLC, ^1^H NMR characterization, and supplementary photochemical data; flow cytometry histograms of integrin expression; dose–response curves of 2D and 3D U87MG cells; bright-field images of U87MG 3D tumor spheroids; intracellular florescence, ROS generation, and ROS species analyses; Apopxin/Nuclear Green double staining FACS data; biodistribution of **Ru-p(HH)** in zebrafish embryos; cytotoxicity test toward zebrafish; confocal images of U87MG tumor burden in zebrafish hindbrain; and the photoactive behaviors of **Ru-p(HH)**, **Ru-p(MH)**, and **Ru-p(MM)** in biological conditions (PDF)
