# Supplementary material for: Cyclic Ruthenium-Peptide Prodrugs Penetrate the Blood–Brain Barrier and Attack Glioblastoma upon Light Activation in Orthotopic Zebrafish Tumor Models
Source: ACS Cent Sci. 2024 Dec 9;10(12):2294–311. doi: 10.1021/acscentsci.4c01173 (PMC11672551; doi:10.1021/acscentsci.4c01173)
Supplement: Supplementary file 1 — oc4c01173_si_001.pdf [file oc4c01173_si_001.pdf]

Supplementary Information

Cyclic ruthenium-peptide prodrugs penetrate the blood brain barrier and attack glioblastoma upon light activation in orthotopic zebrafish tumor models

Liyan Zhang [a§], Gangyin Zhao [b§], Trevor Dalrymple [a], Yurii Husiev [a], Hildert Bronkhorst [a], Gabriel Forn-Cuní [b], Bruno Lopes-Bastos [b], B. Ewa Snaar-Jagalska [b*] & Sylvestre Bonnet [a*]

[a] Leiden Institute of Chemistry, Universiteit Leiden, Einsteinweg 55, 2333 CC, Leiden, Netherlands
Email: bonnet@chem.leidenuniv.nl

[b] Leiden Institute of Biology, Universiteit Leiden, Einsteinweg 55, 2333 CC, Leiden, Netherlands
Email: b.e.snaar-jagalska@biology.leidenuniv.nl

[§] These authors contributed equally: L.Zhang, G. Zhao

Contents

1 General information 1

2 Synthesis 1

3 Photochemistry study 9

4 Integrin αvβ3 and αvβ5 expression by FACS analysis 13

5 Aggregation behaviors in cell culture medium 14

6 Cytotoxicity study on 2D monolayer U87MG cells 14

7 Intracellular florescence intensity of the conjugates during light activation 15

8 Intracellular ROS generation 15

9 Distinguish of ROS 17

10 Cell death mode study 19

11 Cytotoxicity study on 3D multicellular U87MG spheroids 20

12 Drug distribution in zebrafish model 21

13 Cytotoxicity study on zebrafish 25

14 Overview figure 31

1 General information

All reagents were purchased from commercial suppliers. The reactants and solvents were used without further purification. Electrospray ionization mass spectra were recorded by using a MSQ Plus Spectrometer in the positive ionization mode. ^{1}H NMR was obtained on a Bruker DMX-600 spectrometers. HPLC was accomplished by Thermo Scientific Dionex Ultimate 300 system equipped with a 250 x 21.2 mm Jupiter® 4 µm Proteo 90 Å C_{12} column. UV-vis spectra were recorded on a Cary 60 spectrometer from Varian. The emission spectra were measured via an F900 Spectrometer from Edinburgh Instruments Ltd. Absorbance measurements for analysis of 96-well plates were done by a M1000 Tecan Plate Reader. Flow cytometry was conducted by CytoFLEX flow cytometer. For the theoretical modeling, YASARA was used for generating the peptides and to conjugate them to $[Ru(Ph_2phen)_2]^{2+}$ moiety. Human cancer cell lines U87MG (primary glioblastoma cells), MDA-MB-231(human breast cancer cells), A549 (adenocarcinoma alveolar basal epithelial cells), PC-3 (prostate cancer cells) and MCF7 (breast cancer cell) were distributed by the European Collection of Cell Cultures (ECACC) and purchased from Sigma Aldrich. Dulbecco's Modified Eagle Medium (DMEM, D6546), Glutamine-S (GM; 200 mm), penicillin/streptomycin (P/S), tris(hydroxylmethyl)aminomethane (Tris base), trichloroacetic acid (TCA), glacial acetic acid, and sulforhodamine B (SRB) were purchased from Sigma-Aldrich. Opti-MEM Reduced Serum Media without phenol red was obtained from Gibco (11058-021). Rose Bengal and $Ru(bpy)_3Cl_2$ were ordered from Sigma-Aldrich. Cellular ROS Assay Kit (Deep Red, tBHP (tert-Butyl hydroperoxide) included) was purchased from Abcam (ab186029). 9,10-anthracenediyl-bis(methylene)-dimalonic acid (AMDA) and dihydroethidium (DHE) were ordered from Sigma-Aldrich. Anti-integrin $\alpha_V\beta_3$ antibody for integrin expression study was purchased from Merck (MAB1976), Anti-Integrin $\alpha_V\beta_5$ antibody [P1F6] was from Abcam (ab177004). The Alexa Fluor™ 488 conjugated goat anti-mouse IgG (H+L) cross-adsorbed secondary antibody, was ordered from Invitrogen by Thermo Fisher Scientific (A-11001). Apoptosis/Necrosis Detection Kit (blue, red, green) was ordered from Abcam (ab176750).

2 Synthesis

The ligand 4,7-Diphenyl-1,10-phenanthroline as well as $RuCl_3 \cdot nH_2O$ were purchased from Sigma-Aldrich. The peptides Ac-HRGDH-NH_2, Ac-MRGDH-NH_2 Ac-MRGDM-NH_2 were ordered from Biomatik (200 mg, >90 % purity). All reactants and solvents were used without further purification. $[Ru(Ph_2phen)_2(Ac\text{-}MRGDH\text{-}NH_2)]Cl_2$ (**Ru-p(MH)**) was synthesized as

previous described.[1] *cis*-[Ru(Ph_2phen)$_2$$Cl_2$] and [Ru($Ph_2$phen)$_2$(bpy)]$Cl_2$ were synthesized according to literature procedures.[2, 3]

[Ru(Ph_2phen)$_2$(Ac-HRGDH-NH_2)]Cl_2 (Ru-p(HH)). *rac*-[Ru(Ph_2phen)$_2$$Cl_2$] (0.025 mmol, 20.9 mg) was added to a 2-neck 25 mL round-bottom flask, the peptide powder Ac-HRGDH-NH_2 (0.025 mmol, 16.5 mg) was dissolved in demi-water (2.5 mL) and the pH was adjusted to 7.5 using 0.5 mM NaOH and 0.1 mM and HCl solutions. After adding ethanol (2.5 mL), the peptide solution was degassed by N_2 bubbling for 10 min to remove the O_2. The reaction flask was vacuum flushed with N_2 thrice, after which the deoxygenized peptide solution was injected by syringe to the reaction flask under N_2. The mixture was then heated at 80 °C for 3 days. The reaction solution was then cooled down to room temperature, and ethanol was rotary evaporated, leading to precipitation. After filtration of the water phase and washing of the solid by cold water, the filtrate was freeze-dried, to afford a reddish powder. Further purification was accomplished by HPLC. The purification (5-10 mg per injection) was realized by a 250 x 21.2 mm Jupiter® 4 µm Proteo 90 Å C12 column using Thermo Scientific UHPLC system. The gradient was controlled by four pumps. The mobile phase consisted in H_2O containing 0.1% v/v formic acid (phase A) and acetonitrile containing 0.1% v/v formic acid (phase B). The gradient for the preparative separation of **Ru-p(HH)** was 30-40% phase B/phase A for 20 min. The fractions were monitored by four UV detector (set at 214 nm, 290 nm, 350 nm, and 450 nm) and the flow rate was 14 mL/min. The compound was collected at UV-detector 290 nm. After lyophilization, **Ru-p(HH)** was obtained as a red powder (28 % yield, ~10 mg). HPLC (10-90% phase B/phase A, 20 min): t_R = 11.3 min. HR-MS *found* (*calc*): *m/z* = 713.7362 (713.7159 for $[M-2Cl]^{2+}$, $[C_{74}H_{71}N_{17}O_8Ru]^{2+}$) and 476.1600 (476.1599 for $[M-2Cl+H]^{3+}$, $[C_{74}H_{72}N_{17}O_8Ru]^{3+}$).

[Ru(Ph_2phen)$_2$(Ac-MRGDM-NH_2)]Cl_2 (Ru-p(MM)). The exact same procedure was used as for the synthesis of **Ru-p(HH)**, but using the peptide Ac-MRGDM-NH_2 (0.025 mmol, 16.2 mg) instead. The purification (5-10 mg per injection) was realized on a 250 x 21.2 mm Jupiter® 4 µm Proteo 90 Å C12 column using a Thermo Scientific UHPLC system. The gradient was controlled by four pumps. The mobile phase consisted of mixture of H_2O containing 0.1% v/v formic acid (A phase) and acetonitrile containing 0.1% v/v formic acid (B phase). The gradient for **Ru-p(MM)** preparative separation was 25-35% phase B/phase A, for 20 min. The fractions were monitored by four UV detector (214 nm, 290 nm, 350 nm, 450 nm) and the flow rate was 14 mL/min. Compound were collected at UV-detector 290 nm. After lyophilization, **Ru-p(MM)** was obtained as yellow-orange powder (15 % yield, ~5.6 mg). **Ru-p(MM)** HPLC (10-90%

phase B/phase A, 20 min): t_R = 10.5 min. HR-MS *found* (*calc*): *m/z* = 707.7175 (707.7174 for $[M-2Cl]^{2+}$, $[C_{72}H_{75}N_{13}O_8RuS_2]^{2+}$) and 472.1476 (472.1475 for $[M-2Cl+H]^{3+}$, $[C_{72}H_{76}N_{13}O_8RuS_2]^{3+}$).

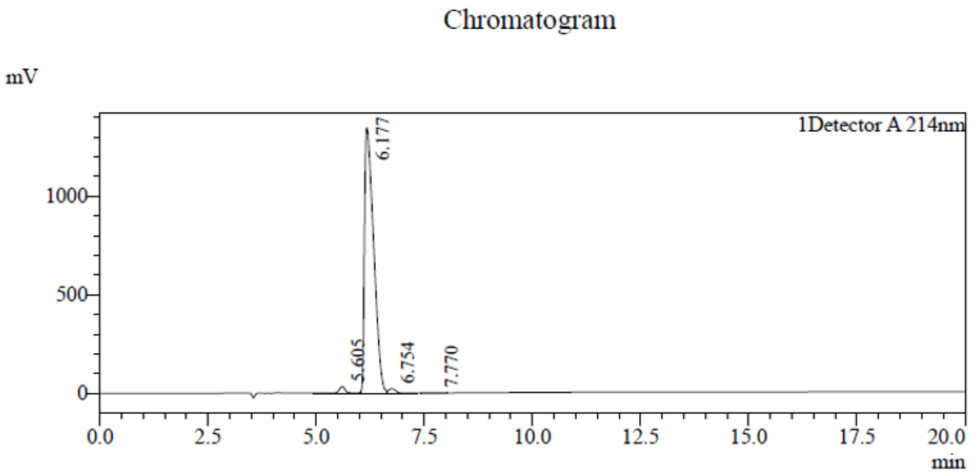

Peak Table

Detector A 214nm

Peak#	Ret. Time	Area	Height	Area%
1	5.605	332579	34251	1.63
2	6.177	19771113	1347876	97.01
3	6.754	266515	23373	1.31
4	7.770	10485	828	0.05
Total		20380692	1406328	100.00

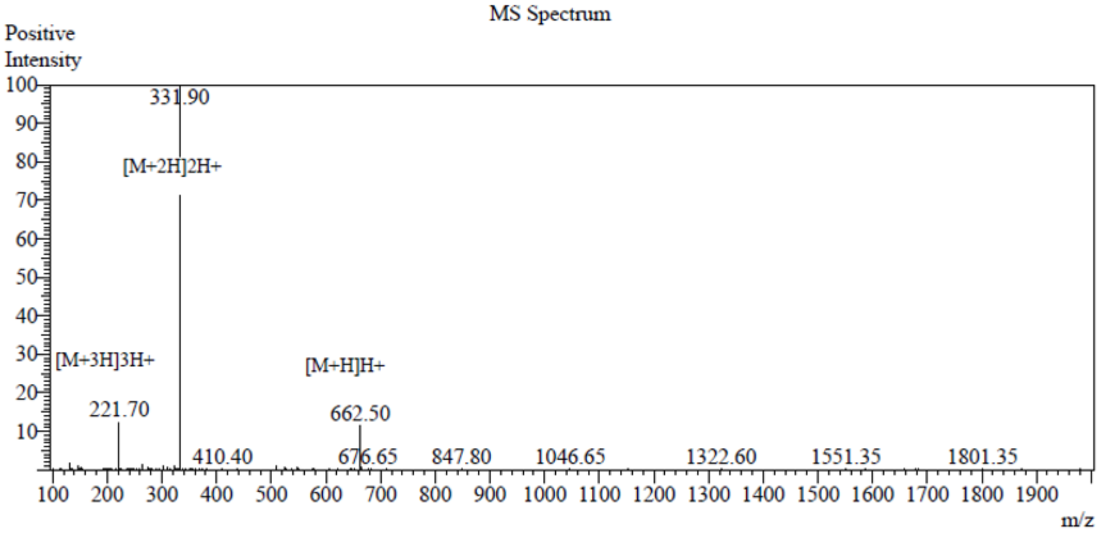

Figure S1. LC-MS chromatogram of peptide Ac-HRGDH-NH_2. Targeted peptide was found at peak t_R=6.177 min, MS: calculated 661.3, found 662.5.

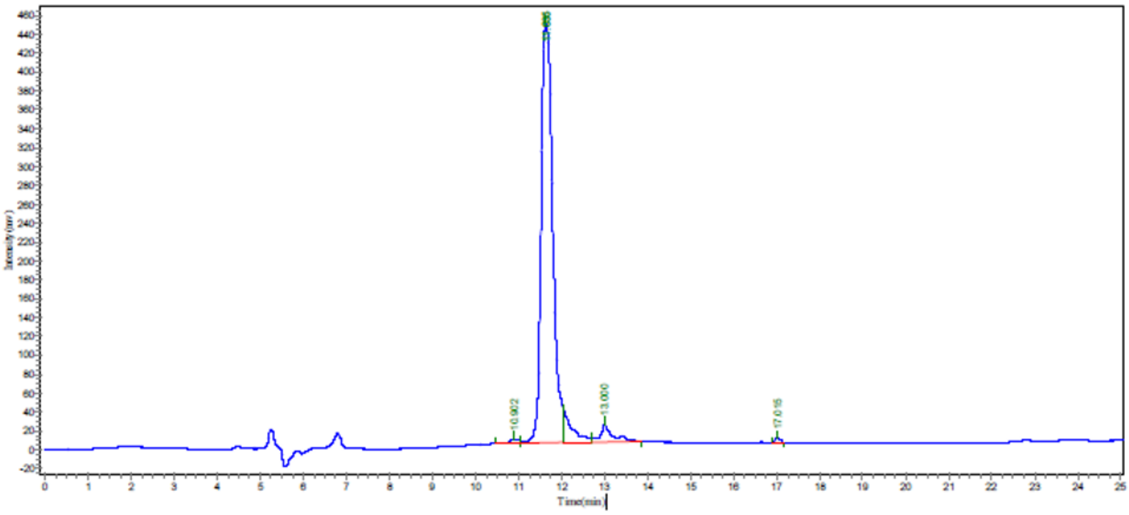

Peak No.	Ret Time	Height	Area	Conc..
1	10.902	4013.682	55146.316	0.5960
2	11.635	444109.000	8338015.500	90.1191
3	11.635	34635.734	468611.313	5.0649
4	13.000	18573.850	359479.156	3.8853
5	17.015	3988.364	30964.795	0.3347
Total				100.0000

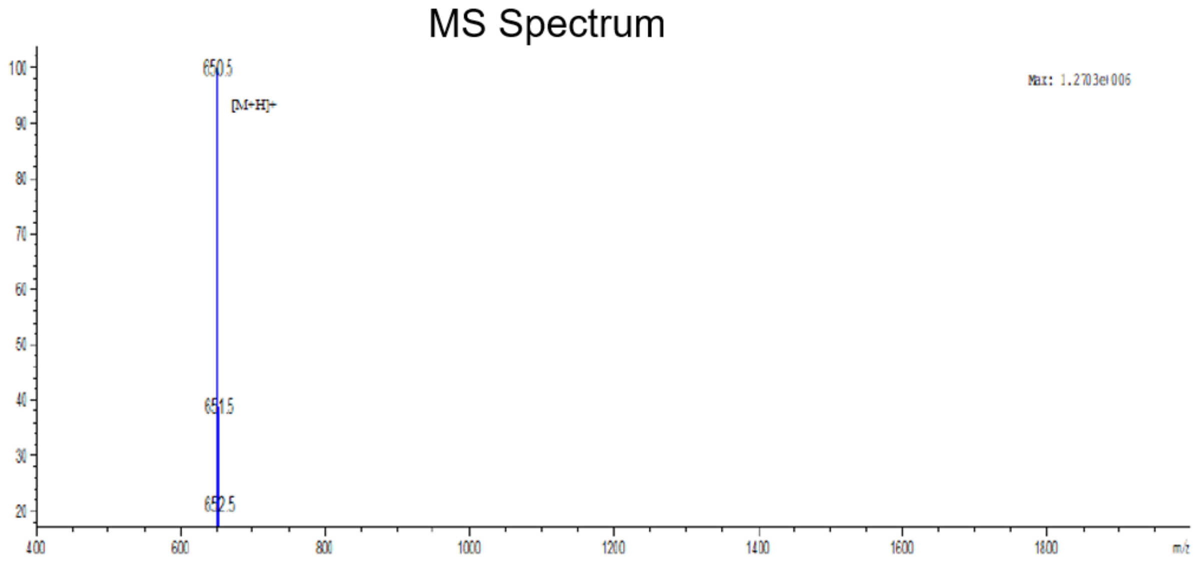

Figure S2. LC-MS chromatogram of peptide Ac-MRGDM-NH_2. Targeted peptide was found at peak t_R=11.635 min, MS: calculated 649.3, found 650.5.

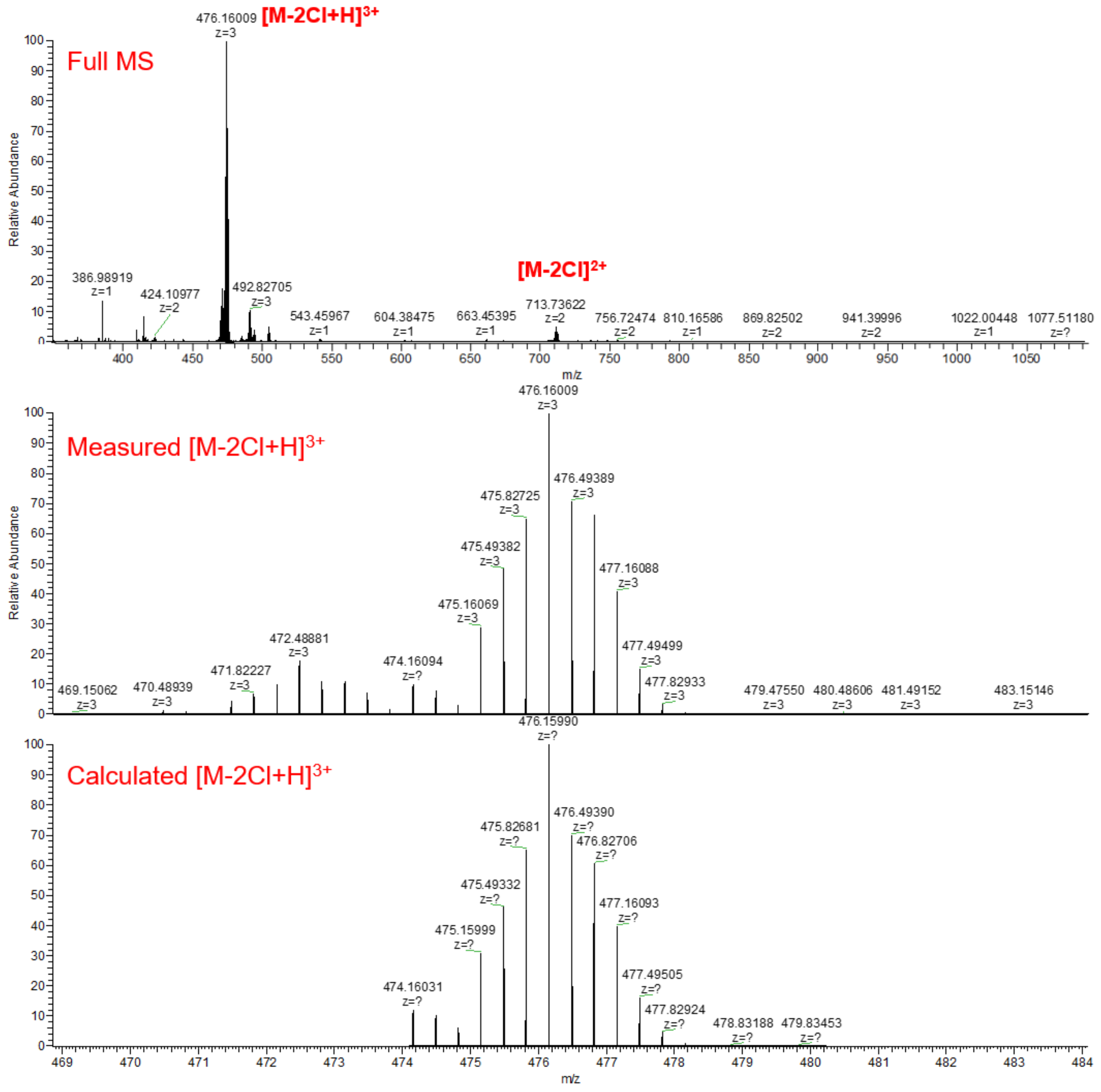

Figure S3. High resolution mass spectra of **Ru-p(HH)** in acetonitrile after HPLC purification. (calc. m/z for [**M-2Cl**]$^{2+}$ = 713.7159 and [**M-2Cl+H**]$^{3+}$ = 476.1599).

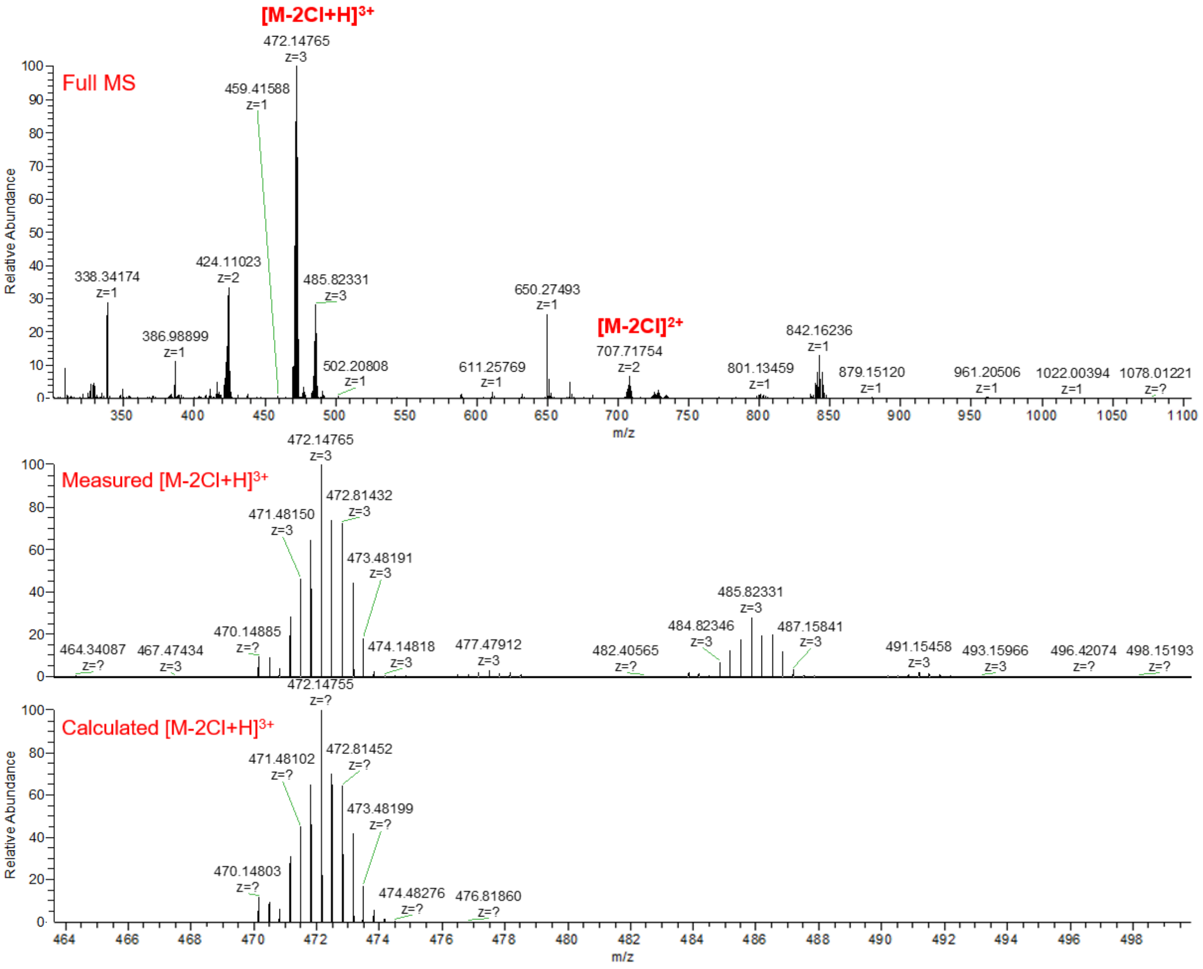

Figure S4. High resolution mass spectra of **Ru-p(MM)** in acetonitrile after HPLC purification. (calc. m/z for [**M-2Cl**]$^{2+}$ = 707.7174 and [**M-2Cl+H**]$^{3+}$ = 472.1475).

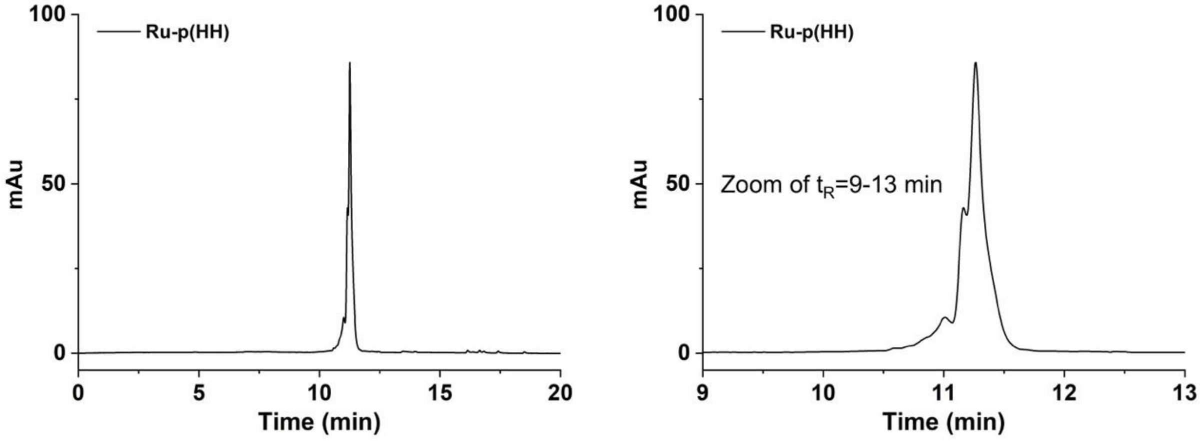

Figure S5. HPLC trace of **Ru-p(HH)** (t_R=11.3 min) after purification (Λ and Δ isomers were not separated). Gradient: 10-90% phase B (acetonitrile with 0.1% v/v formic acid)/phase A (H_2O with 0.1% v/v formic acid), 20 min, detector UV channel=290 nm. The side minor peak at t_R=11 min is possibly attributed to [Ru(Ph_2phen)$_2$(Ac-HRGDH-NH_2) (H_2O)]Cl_2.

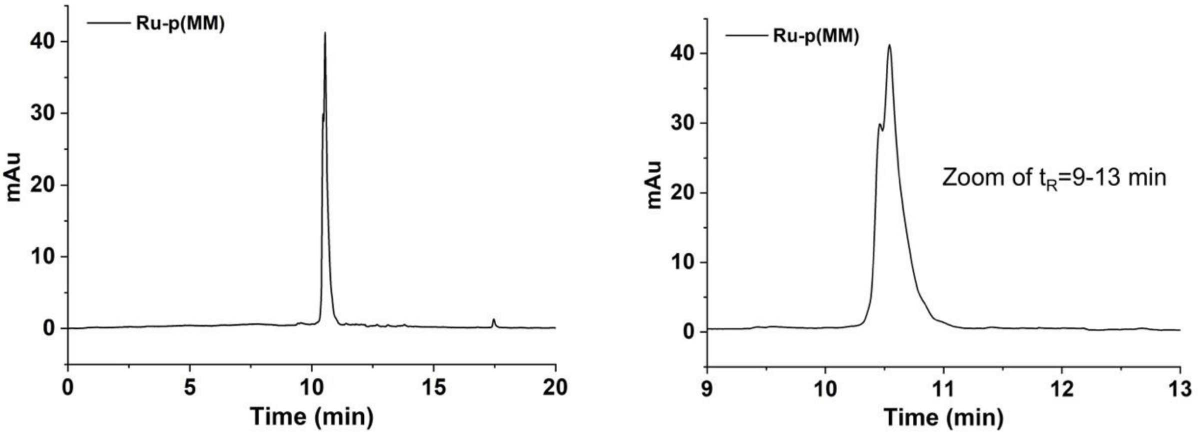

Figure S6. HPLC trace of **Ru-p(MM)** (t_R=10.5 min) after purification (Λ and Δ isomers were not separated). Gradient: 10-90% phase B (acetonitrile with 0.1% v/v formic acid)/phase A (H_2O with 0.1% v/v formic acid), 20 min, detector UV channel=290 nm.

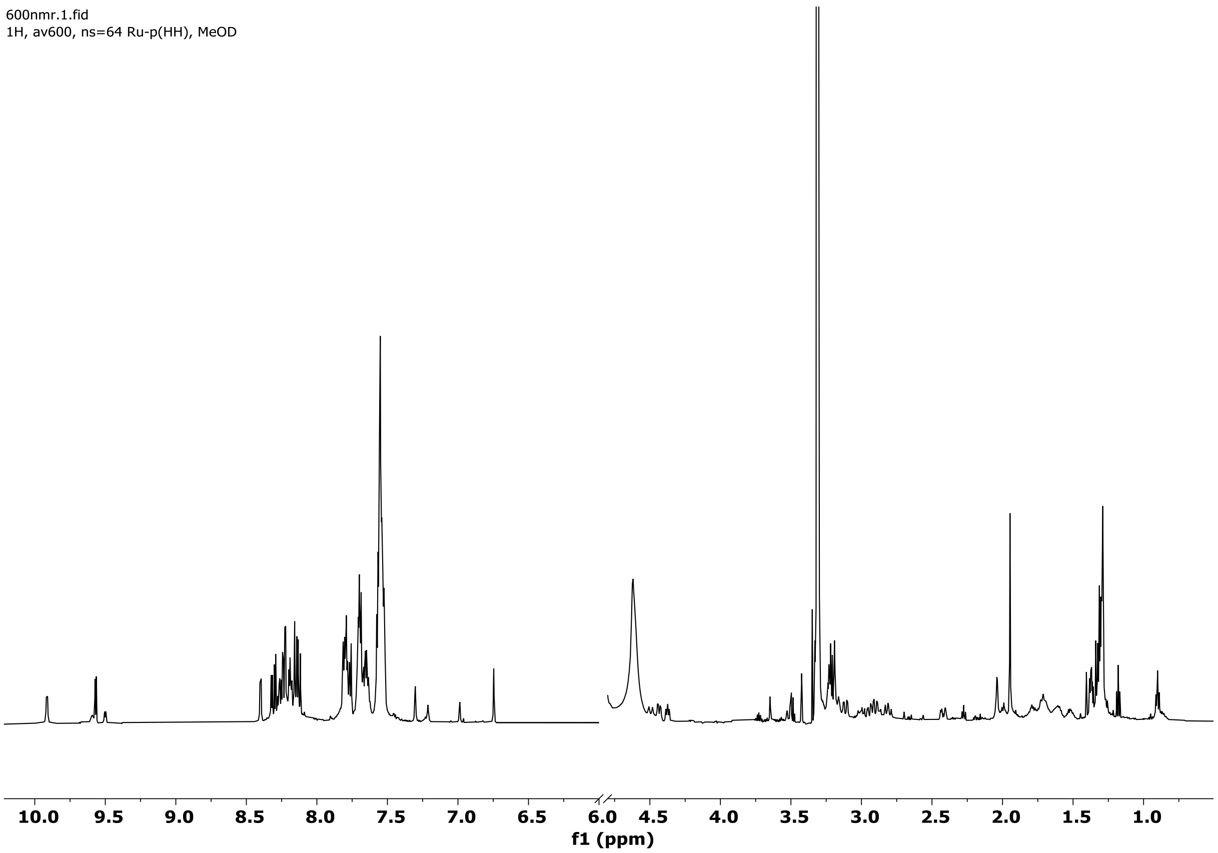

Figure S7. ^{1}H NMR (600 MHz, MeOD) spectra of **Ru-p(HH)**.

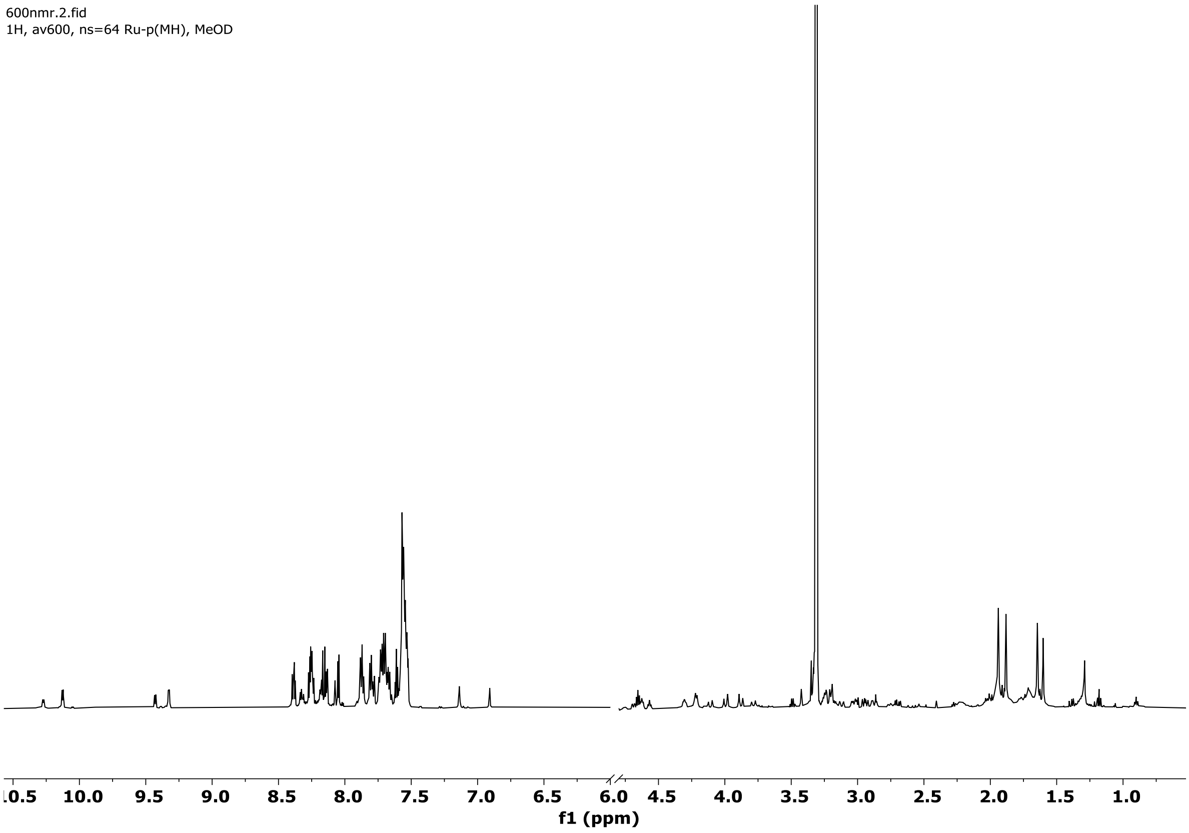

Figure S8. ^{1}H NMR (600 MHz, MeOD) spectra of **Ru-p(MH)**.

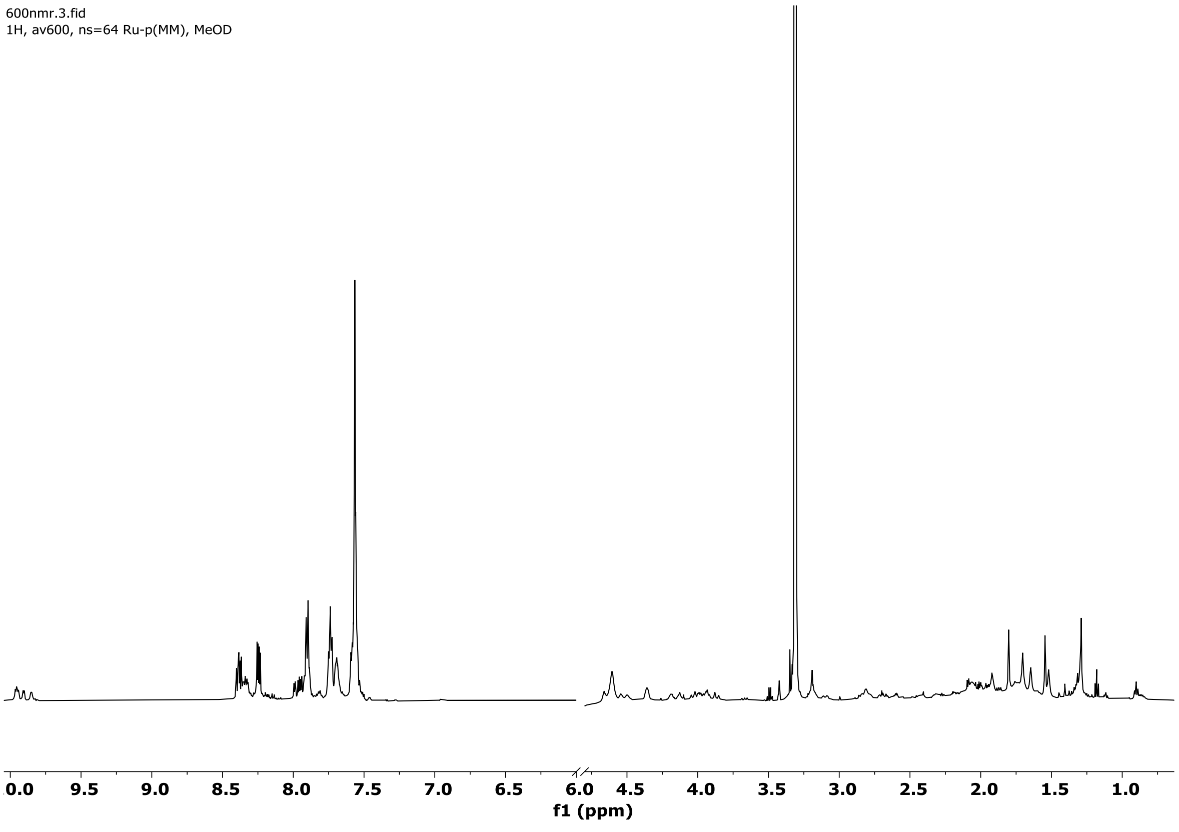

Figure S9. ^{1}H NMR (600 MHz, MeOD) spectra of **Ru-p(MM)**.

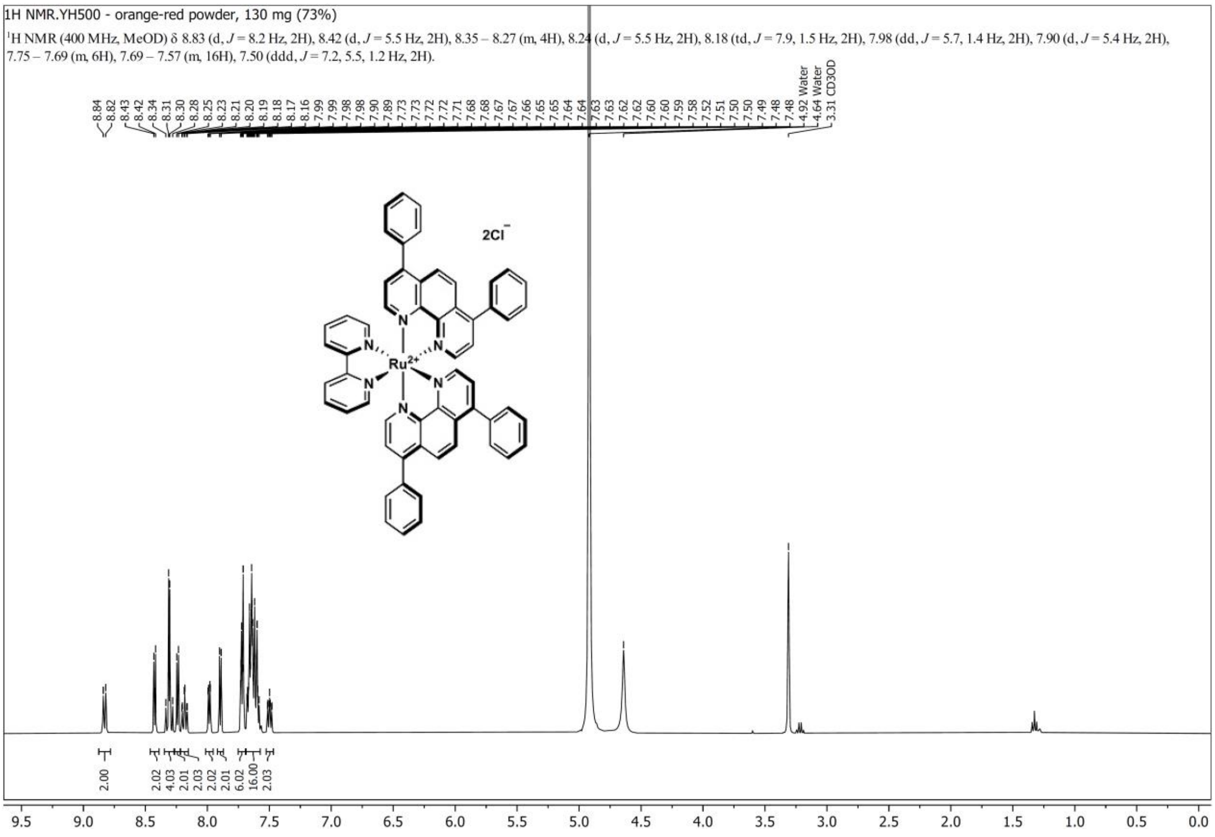

Figure S10. ^{1}H NMR (400 MHz, MeOD) spectra of **[Ru(Ph$_2$phen)$_2$(bpy)]Cl$_2$**.

3 Photochemistry study

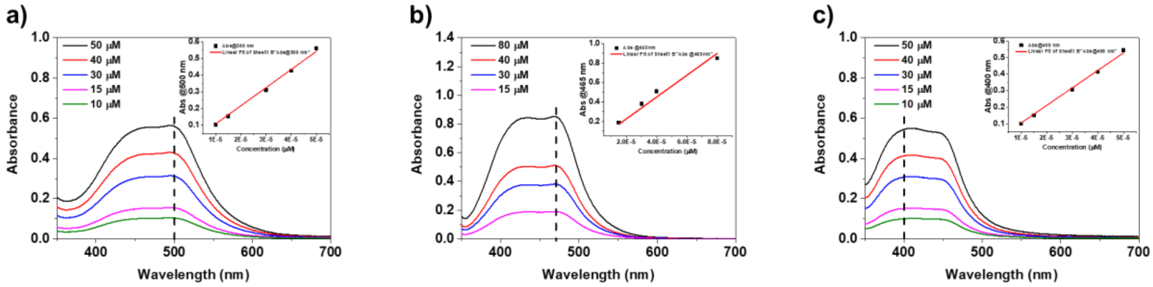

Figure S11. Absorbance spectra of **Ru-p(HH)**, **Ru-p(MH)** and **Ru-p(MM)** dissolved in MilliQ H_2O at different concentrations, showing the absence of aggregation in such conditions.

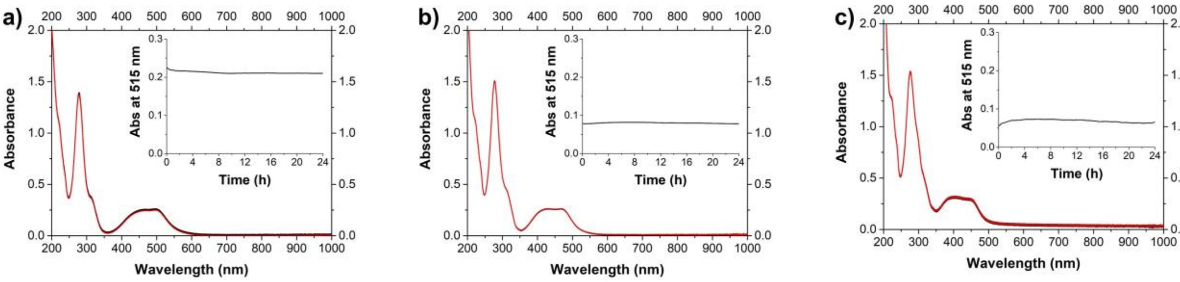

Figure S12. Time evolution of the UV-vis spectra of **Ru-p(HH)** (a), **Ru-p(MH)** (b) and **Ru-**

p(MM) (c) (30 μM, H_2O) in the dark for 24 h. Insert: time evolution of the absorbance at 515 nm *vs.* time. Temperature: 298 K.

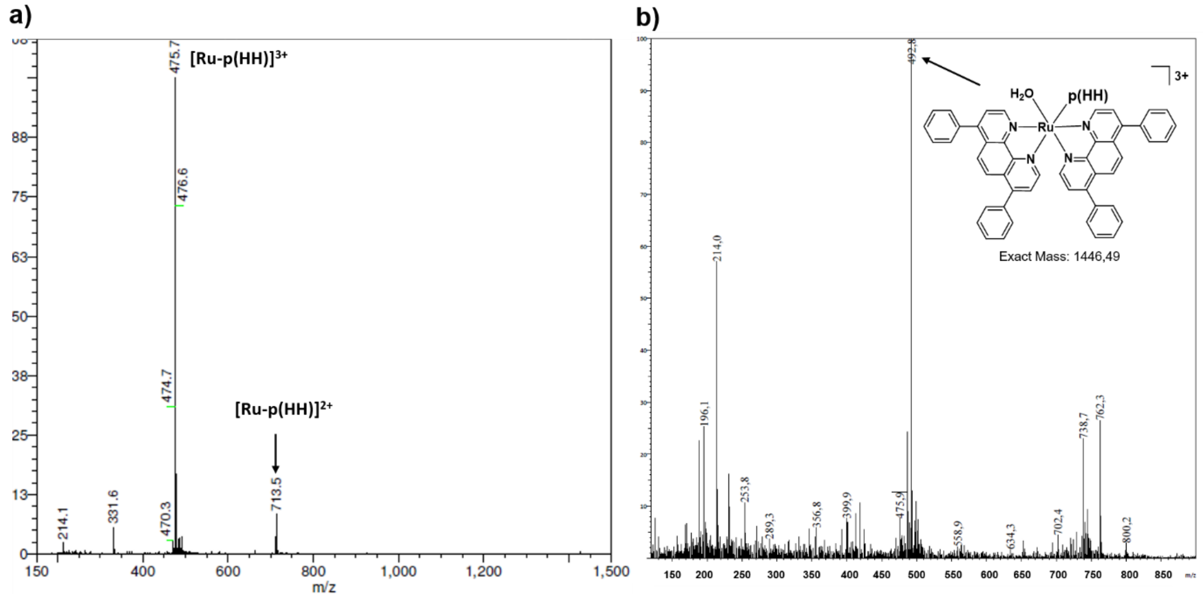

Figure S13. Mass spectra of **Ru-p(HH)** in H_2O under dark (a), or after green light (515 nm) irradiation for 2 h in H_2O (b), Photoactive product [Ru(Ph$_2$phen)$_2$(Ac-HRGDH-NH$_2$)(H_2O) + MeOH]$^{3+}$ (found : 492.8, calc. m/z = 492.8).

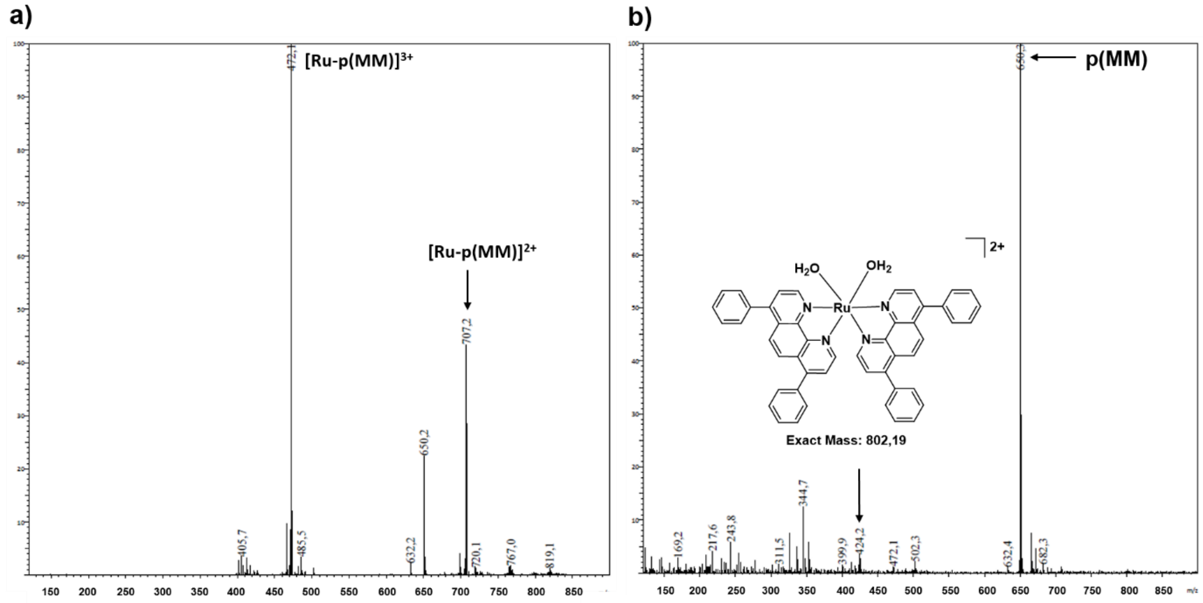

Figure S14. Mass spectra of **Ru-p(MM)** in H_2O in the dark (a), or after green light (515 nm) irradiation for 2 h in H_2O (b), Photoactive product [Ru(Ph$_2$phen)$_2$(H_2O)$_2$]$^{2+}$ + formic acid (found : 424.2, calc. m/z = 424.1), and free peptide Ac-MRGDM-NH$_2$ (found : 650.3, calc. m/z = 650.3).

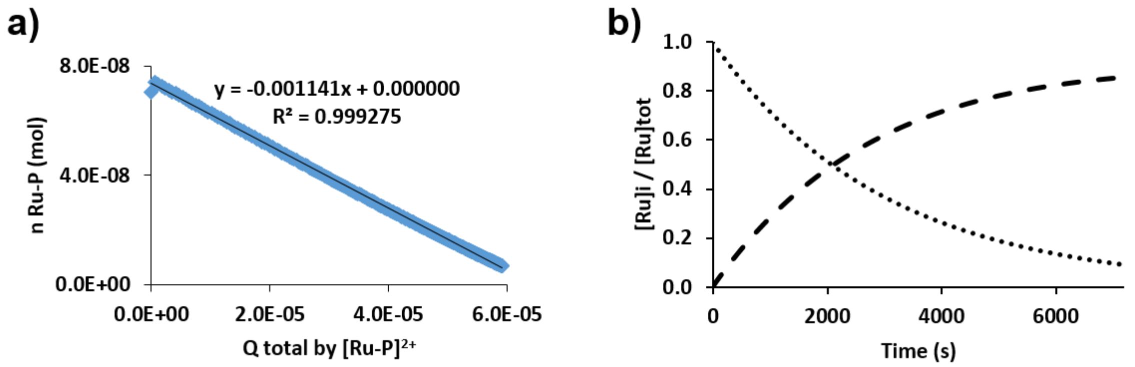

Figure S15. Fitting of the time evolution of the UV-vis spectrum of a solution of **Ru-p(HH)** in H_2O irradiated with green light (515 nm, 4.0 mW/cm^2). (a) Amount of Ru-peptide reagent (Ru-P) plotted *vs*. the number of photons Q absorbed by Ru-P since t=0 (in mol). (b) Evolution of the relative concentrations of Ru-P reactant (dotted line) and photoproduct (solid line) according to global fitting using Glotaran. The absolute value of the slope of the trendline in a) corresponds to the quantum yield for the one-step photosubstitution reaction of **Ru-p(HH)**.

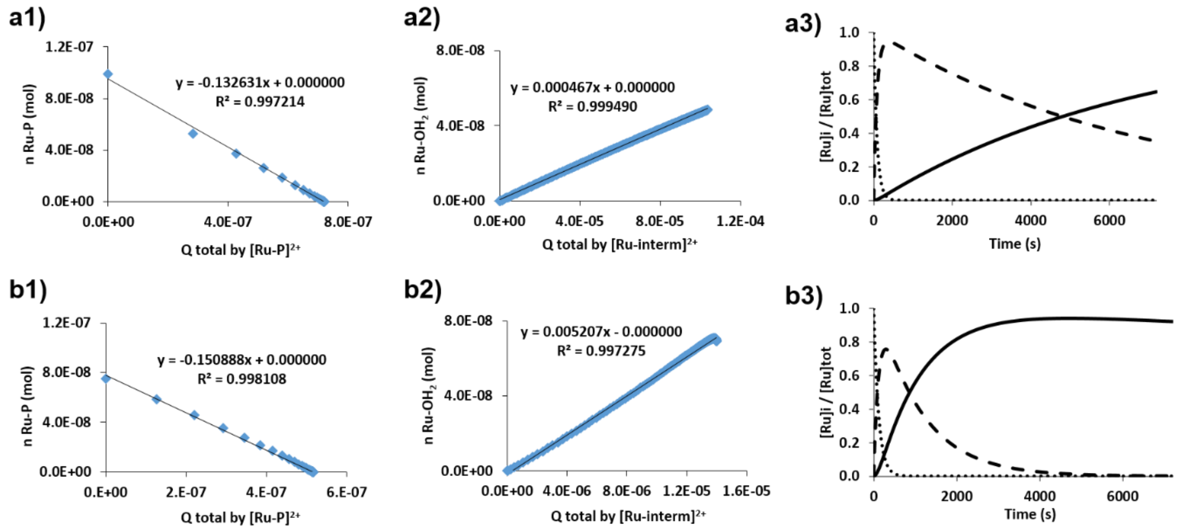

Figure S16. Fitting of the time evolution of the UV-vis spectrum of a solution of **Ru-p(MH)** (a) or **Ru-p(MM)** (b) in H_2O irradiated with green light (515 nm, 4.0 mW/cm^2). (1) Amount of Ru-peptide reagent (Ru-P) plotted *vs*. the number of photons Q absorbed by Ru-P since t=0 (in mol). (2) Amount of photosubstituted product ($Ru\text{-}OH_2$) generated *vs*. the number of photons Q absorbed by the η^1-intermediate. (3) Evolution of the relative concentrations of Ru-P reactant (dotted line), η^1-intermediate (dashed line) and photoproduct (solid line) according to global fitting using Glotaran. The absolute value of the slopes of the trendlines in 1) and 2) correspond to the quantum yields for the two consecutive steps in the two photosubstitution reactions.

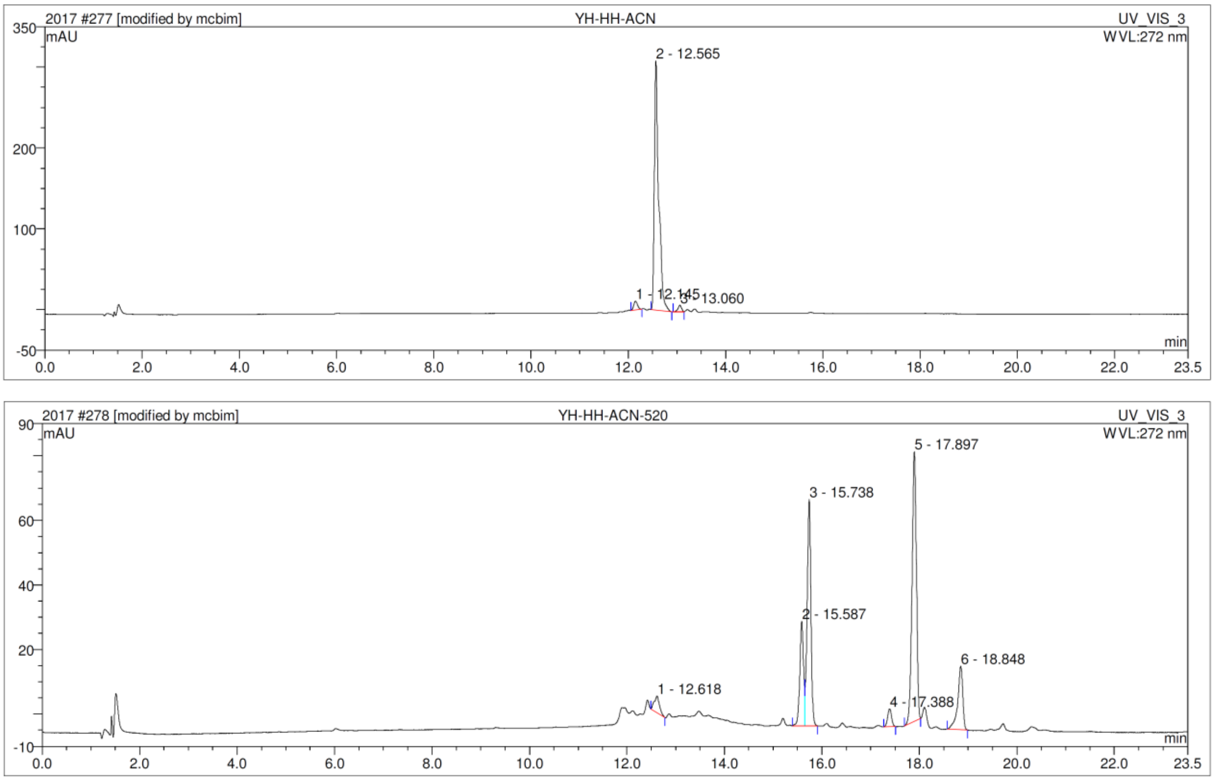

Figure S17. HPLC traces of **Ru-p(HH)** (t_R = 12.565 min) before (top) and after (bottom) irradiation with green 520 nm light for 30 min in ACN (0.1 mM solution). Compounds at 12.565 min, 15.587 min, 17.897 min and 18.848 min show absorbance at 520 nm and thus belong to ruthenium(II) complexes. Gradient elution: 10 to 90% ACN in H_2O (+0.1% v/v formic acid) for 23.5 min; UV detector: 272 nm.

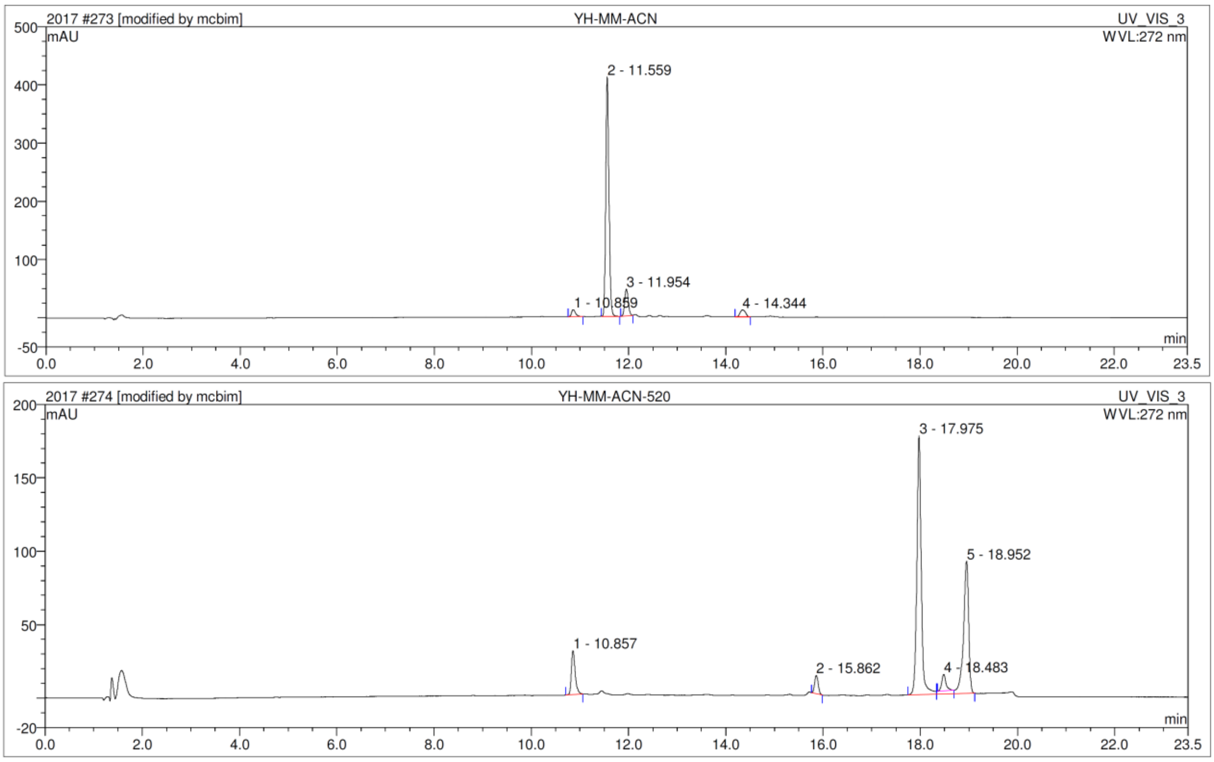

Figure S18. HPLC traces of **Ru-p(MM)** (t_R = 11.559 min) before (top) and after (bottom) irradiation with green 520 nm light for 30 min in ACN (0.1 mM solution). Compounds at 11.559 min, 14.344 min, 17.975 min and 18.952 min show absorbance at 520 nm and thus belong to ruthenium(II) complexes. Gradient elution: 10 to 90% ACN in H_2O (+0.1% v/v formic acid) for 23.5 min; UV detector: 272 nm.

4 Integrin $\alpha_v\beta_3$ and $\alpha_v\beta_5$ expression by FACS analysis

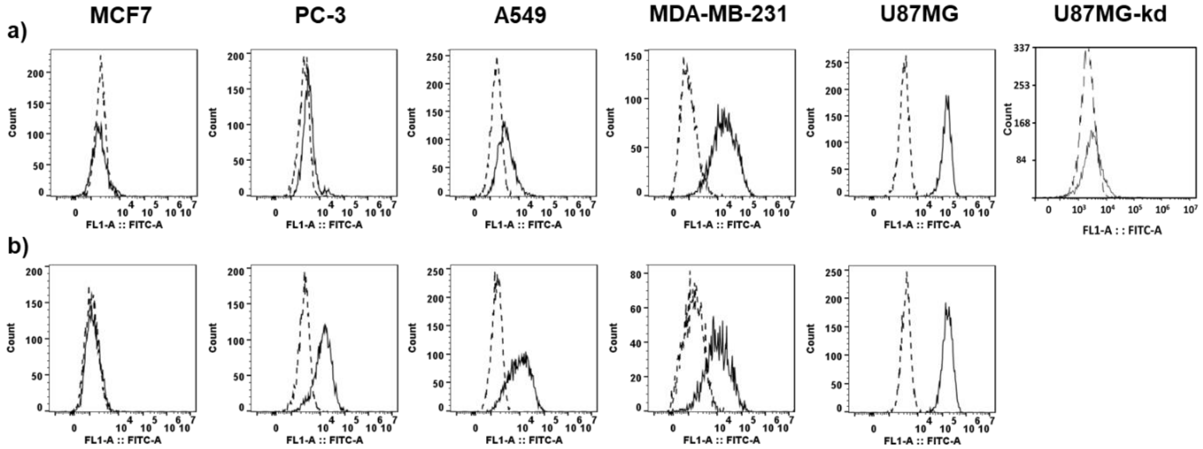

Figure S19. a) Representative flow cytometry histogram of integrin $a_V\beta_3$ expression from MCF7, PC-3, A549, MDA-MB-231 U87MG and U87MG-kd cells cultured under normoxia (21% O_2, a) or hypoxia (1% O_2, b). Solid lines represent the fluorescence intensity of the cells after the incubation with anti-integrin $a_V\beta_3$ first antibody followed by Alexa Fluor™ 488 conjugated goat anti-mouse IgG second antibody. Dotted lines indicate the background staining obtained when only the secondary antibody was used, which are considered as controls.

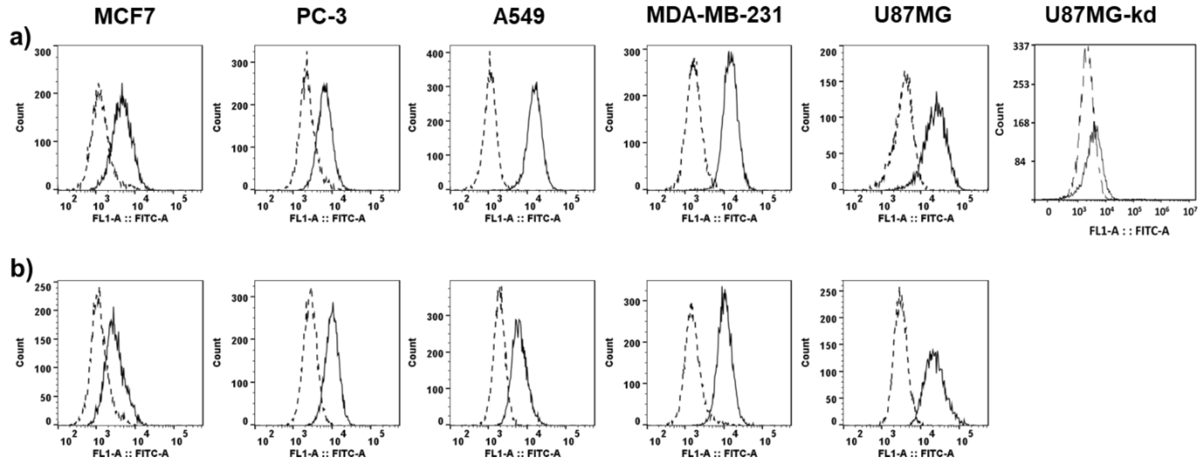

Figure S20. a) Representative flow cytometry histogram of integrin $a_V\beta_5$ expression from MCF7, PC-3, A549, MDA-MB-231, U87MG and U87MG-kd cells cultured under normoxia

(21% O_2, a) or hypoxia (1% O_2, b). Solid lines represent the fluorescence intensity of the cells after the incubation with anti-integrin $a_V\beta_3$ first antibody followed by Alexa Fluor™ 488 conjugated goat anti-mouse IgG second antibody. Dotted lines indicate the background staining obtained when only the secondary antibody was used, which are considered as controls.

5 Aggregation behaviors in cell culture medium

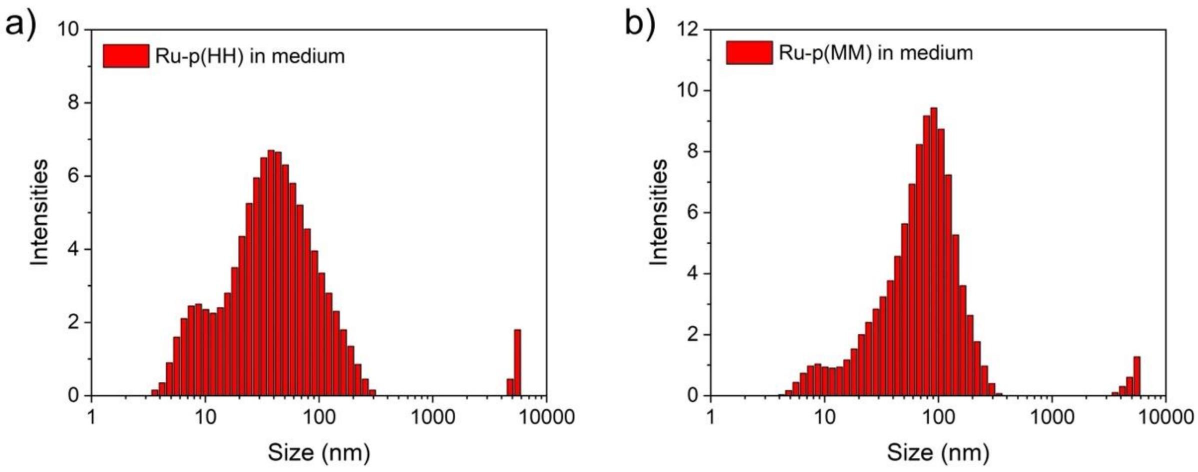

Figure S21. Dynamic Light Scattering (DLS) of **Ru-p(HH)** (a) and **Ru-p(MM)** (b) in Opti-MEM (50 µM) with 2.5% Fetal calf serum (FCS).

6 Cytotoxicity study on 2D monolayer U87MG cells.

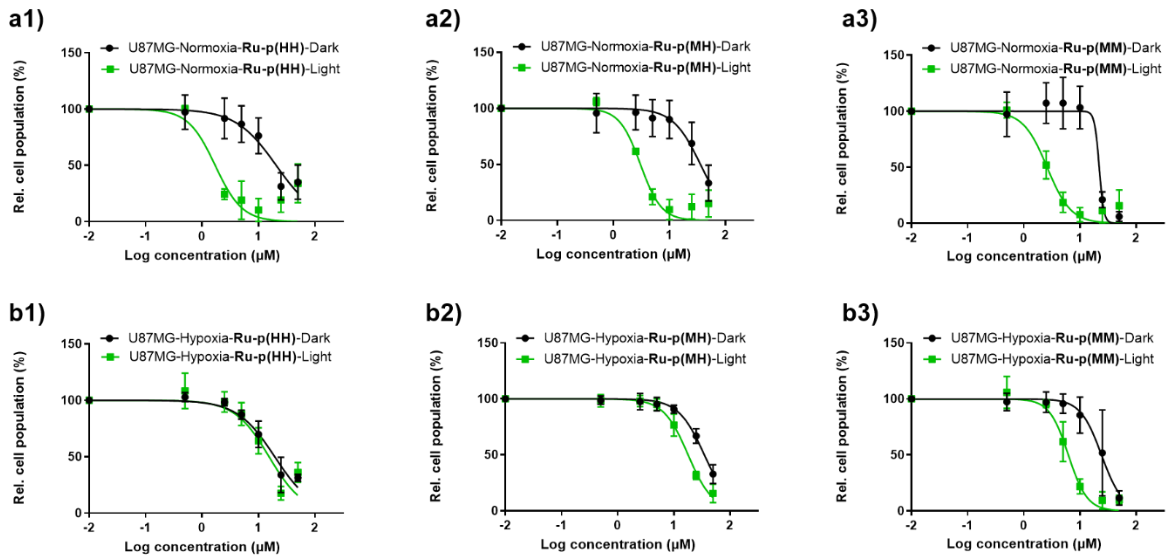

Figure S22. Dose-response curves of 2D monolayer U87MG cells after treatment with 0.5-50 µM of **Ru-p(HH)** (1), **Ru-p(MH)** (2) and **Ru-p(MM)** (3) in normoxia (a) or in hypoxia(b). Normoxia: 37 °C, 21% O_2 and 5.0 % CO_2; Hypoxia: 37 °C, 1% O_2 and 5.0% CO_2; black curve: dark condition; green curve: irradiated with green light (Normoxia: 520 nm, 10.9 mW cm^{-2}, 13.1 J cm^{-2}, 20 min and hypoxia: 520 nm, 7.22 mW cm^{-2}, 13.1 J cm^{-2}, 30 min).

7 Intracellular florescence intensity of the conjugates during light activation

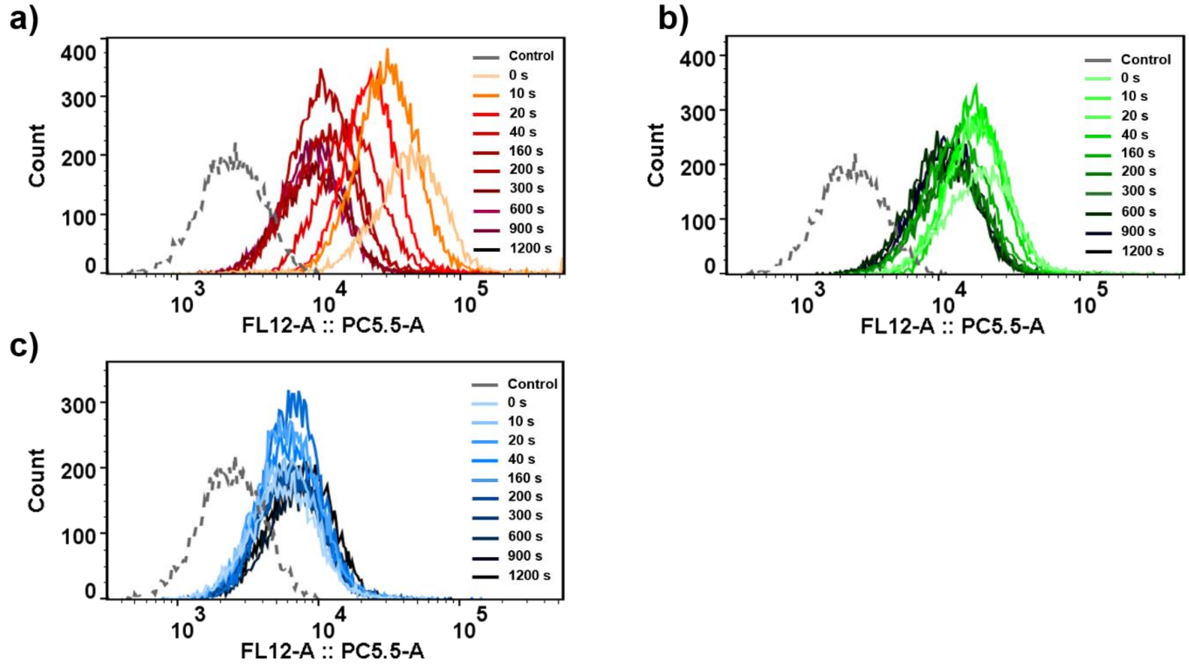

Figure S23. Flow cytometry histograms of U87MG cells after treated with **Ru-p(HH)** (a), **Ru-p(MH)** (b) and **Ru-p(MM)** (c) **(**15 μM, 24 h) and then washed and irradiated with green light (520 nm, intensity = 10.9 mW cm^{-2}) for different time slots, including 0 s, 10 s, 20 s, 40 s, 160 s, 200 s, 300 s, 600 s, 900 s or 1200 s. Untreated cells were used as control, X-axis represents the florescence intensity detected by PC5.5 channel (488 nm excitation, 650 ± 50 nm emission) of FACS.

8 Intracellular ROS generation

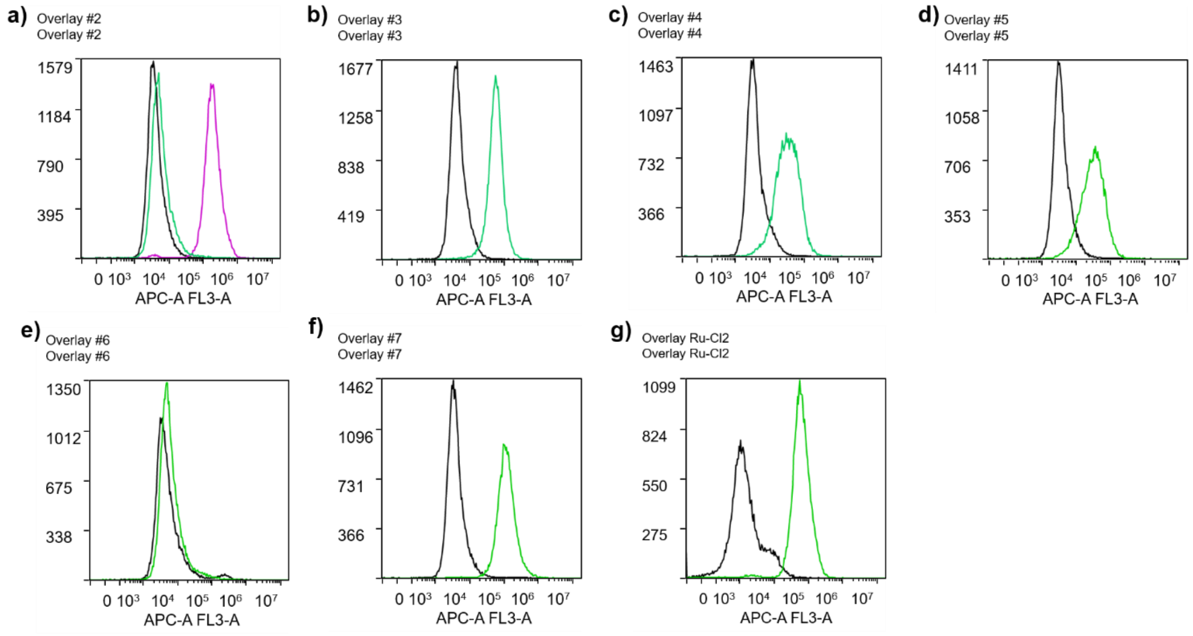

Figure S24. Reactive Oxygen Species generation in U87MG cells under normoxia (21% O_2) according to FACS analysis using CellROX™ Deep Red Reagent as ROS probe, after treated with medium only or tBHP (a, 250 μM, positive control), and complexes (15 μM, 24 h) **Ru-p(HH)** (b), **Ru-p(MH)** (c), **Ru-p(MM) (d),** Cisplatin (e), Rose Bengal (f) or Ru(Ph_2phen)$_2Cl_2$ (g) in the dark or after light irradiation (515 nm, 13.1 J cm^{-2}). Dark group (black curve), light group (green curve) and tBHP (purple curve) samples are shown. X-axis represents the intensity of the ROS probe detected by APC-A channel (638 nm excitation, 660/10 nm emission) of FACS; higher value means higher ROS generation and Y-axis represents cell count.

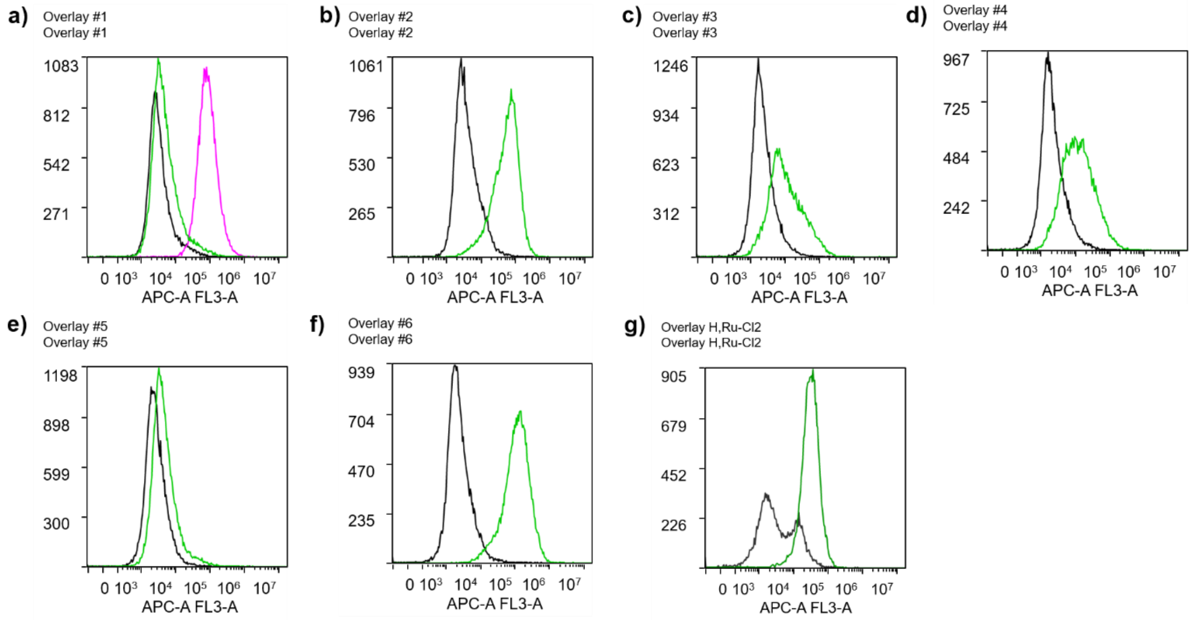

Figure S25. Reactive Oxygen Species generation in U87MG cells under hypoxia (1% O_2) according to FACS analysis using CellROX™ Deep Red Reagent as ROS probe, after treated with medium only or tBHP (a, 250 μM, positive control), and complexes (15 μM, 24 h) **Ru-p(HH)** (b), **Ru-p(MH)** (c), **Ru-p(MM)** (d), Cisplatin (e), Rose Bengal (f) or Ru(Ph_2phen)$_2Cl_2$ (g) in the dark or after light irradiation (515 nm, 13.1 J cm^{-2}). Dark group (black curve), light group (green curve) and tBHP (purple curve) samples are shown. X-axis represents the emission intensity of the ROS probe detected by APC-A channel (638 nm excitation, 660/10 nm emission) of FACS (higher value means higher ROS generation in one given cell) and the Y-axis represents cell count.

9 Distinguish of ROS

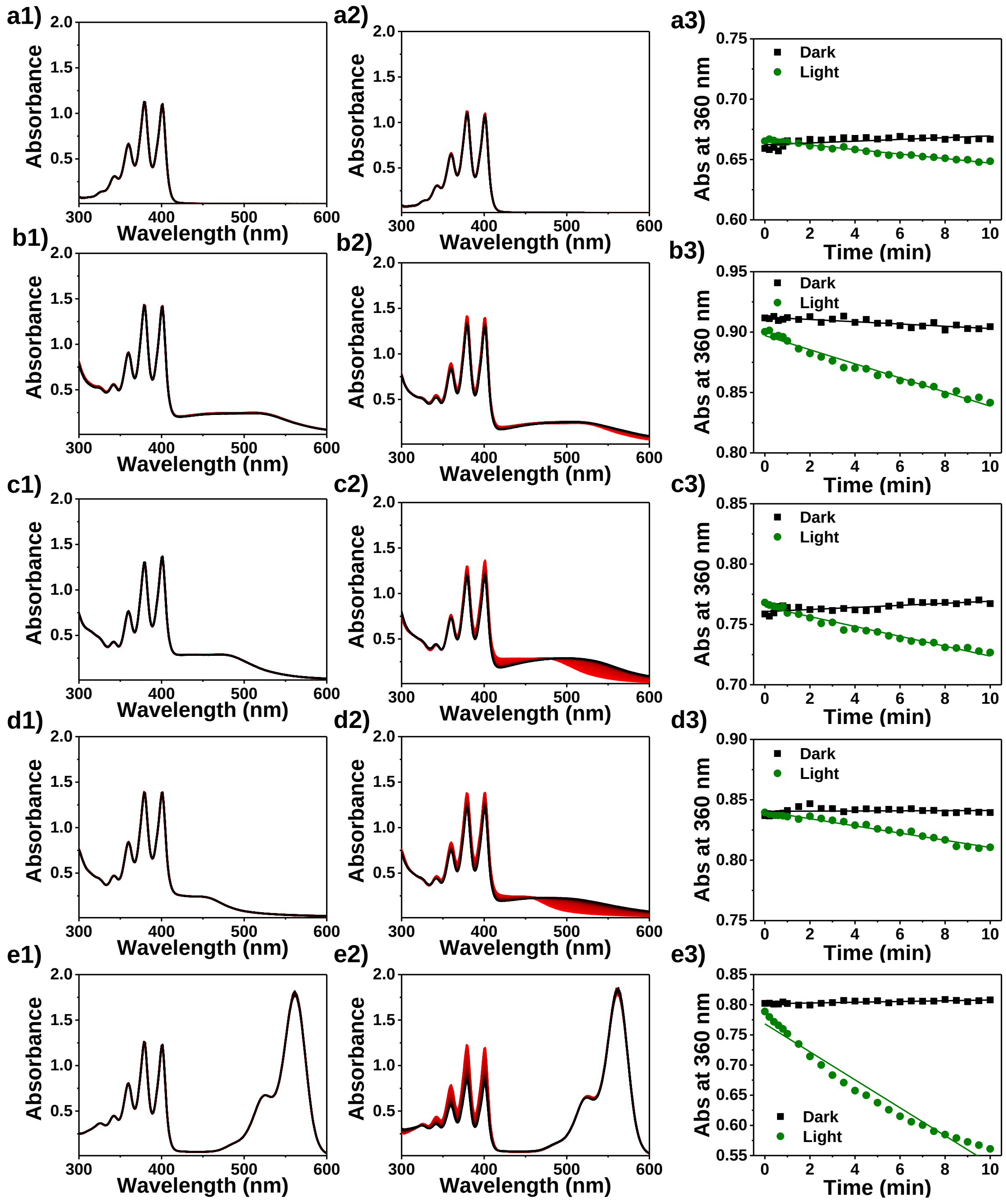

Figure S26. Time evolution of the absorption spectrum of 9,10-anthracenediyl-bis(methylene)-dimalonic acid (ABDA, 100 µM, Opti-MEM complete solution) in the absence (a) or presence of **Ru-p(HH)** (b), **Ru-p(MH)** (c), **Ru-p(MM)** (d), or Rose Bengal (e) (20 µM), in the dark (1) or under green light irradiation (2, 515 nm, 4.0 mW/cm^2) for 10 min. Right graphs show the evolution of the absorbance at 360 nm *vs.* irradiation time (3) in both dark and light groups. The green dots were optimized by eliminating the effect of the absorbance evolution of the complexes during irradiation without ABDA.

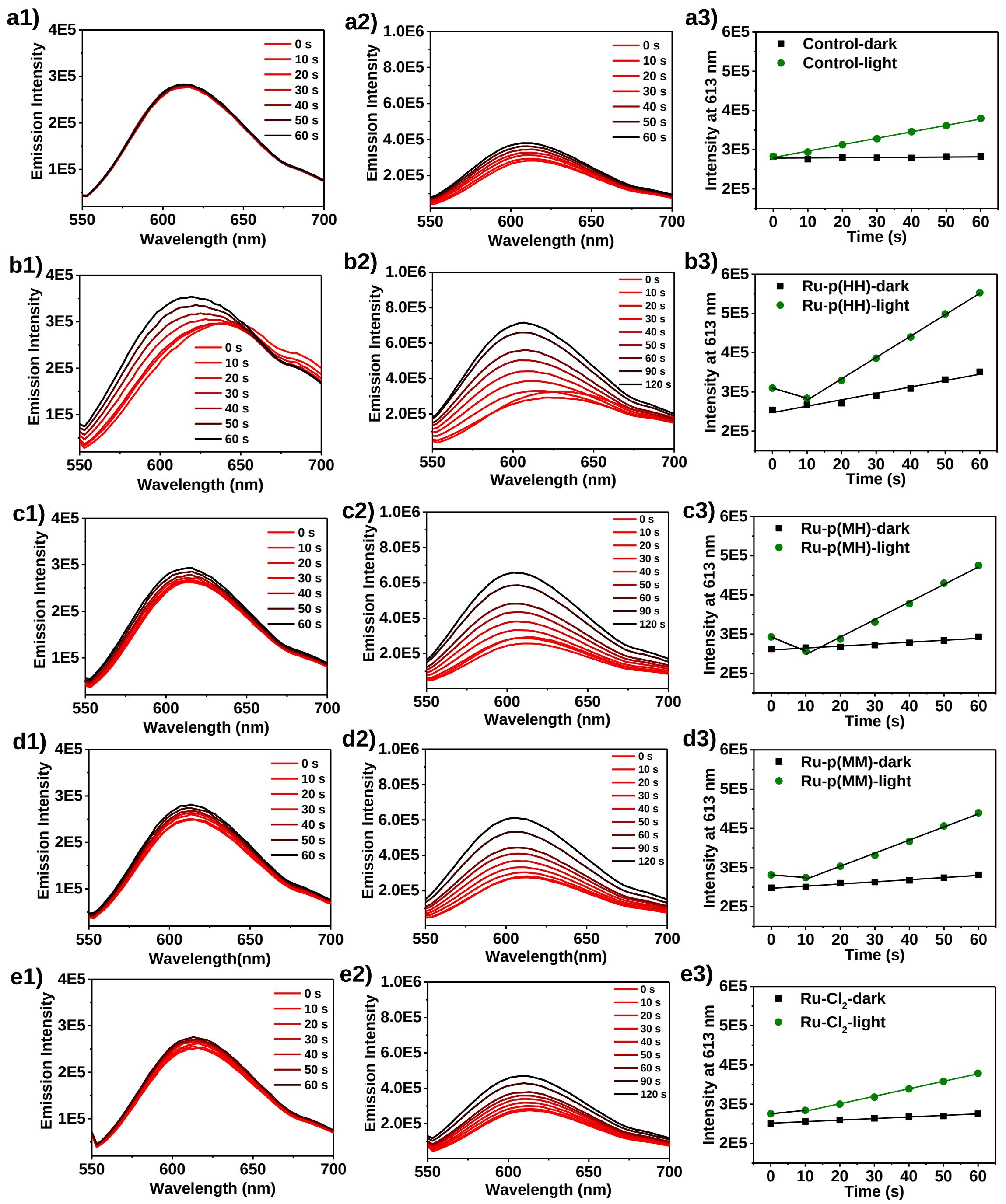

Figure S27. The emission spectra of dihydroethidium (DHE) solution (λ_{exc}=535 nm, 100 μM, Opti-MEM complete solution) in the absence (a) or presence of **Ru-p(HH)** (b), **Ru-p(MH)** (c), **Ru-p(MM)** (d), or $Ru(Ph_2phen)_2Cl_2$ (simplified by $Ru\text{-}Cl_2$, e) (20 μM), in the dark (1) or under green light irradiation (2, 515 nm, 4.0 mW/cm^2) for 60 s. Right graphs show emission intensity at 613 nm *vs.* irradiation time (3) in both dark and light groups.

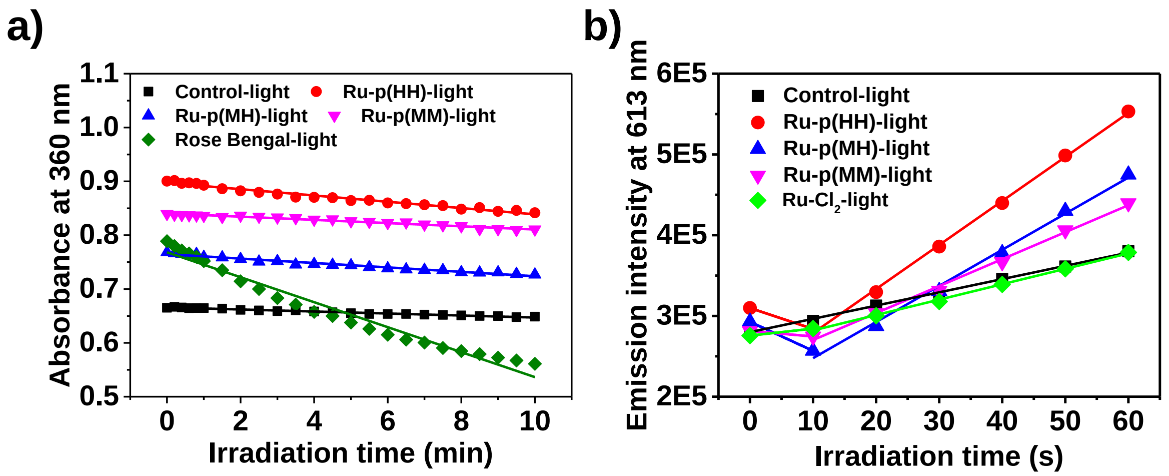

Figure S28. (a) The absorbance at 360 nm of AMDA in Opti-MEM complete solution (100 µM) in the absence (control) or presence of **Ru-p(HH)**, **Ru-p(MH)**, **Ru-p(MM)**, or Rose Bengal (20 µM) *vs.* irradiation time (515 nm, 4.0 mW/cm^2) over 10 min. (b) The emission intensity at 613 nm of DHE in Opti-MEM complete solution (λ_{exc}=535 nm, 100 µM) in the absence (control) or presence of **Ru-p(HH)**, **Ru-p(MH)**, **Ru-p(MM)**, or $Ru(Ph_2phen)_2Cl_2$ (20 µM, the latter being noted as $Ru\text{-}Cl_2$) *vs.* irradiation time (515 nm, 4.0 mW/cm^2) over 60 s. Absolute slope values represent the efficiency of ROS generation, slopes regarding to $O_2^{\bullet-}$ generation were only calculated after 10 s.

10 Cell death mode study

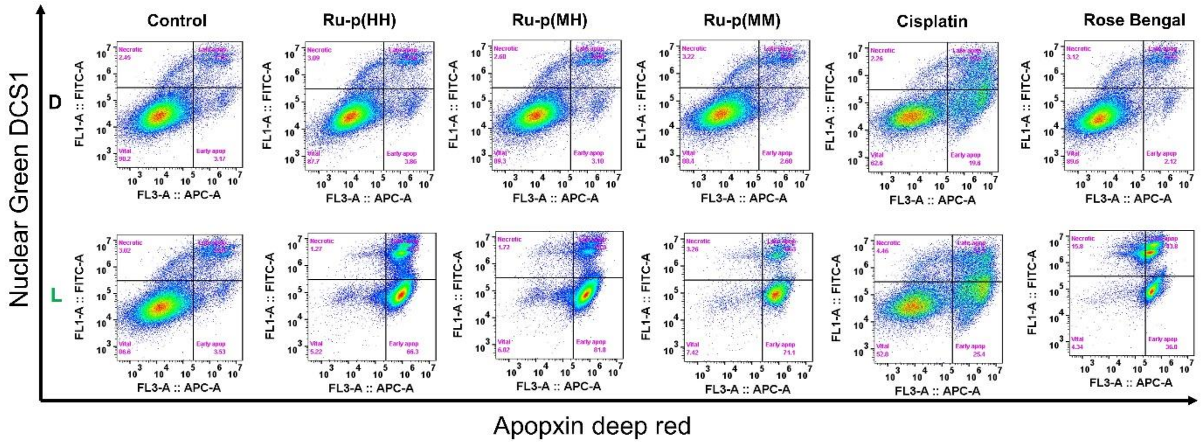

Figure S29. Apopxin/Nuclear Green double staining FACS data from U87MG cells upon treatment with vehicle control (medium), **Ru-p(HH)**, **Ru-p(MH)**, **Ru-p(MM)**, Cisplatin, Rose Bengal (20 µM, 24 h) by flow cytometry using ab176749 Apoptosis/Necrosis Assay Kit. Conditions: normoxia (21% O_2), dark or irradiated with green light (520 nm, 13.1 J cm^{-2}). Axes are logarithmic, showing Nuclear Green DCS1 (probe of necrosis) fluorescence values detected

by FITC channel (488 nm excitation, 525/40 nm emission) of FACS on the Y-axis, and Apopxin Deep Red (probe of apoptosis) fluorescence values detected by APC-A channel (638 nm excitation, 660/10 nm emission) of FACS on the X-axis.

11 Cytotoxicity study on 3D multicellular U87MG spheroids

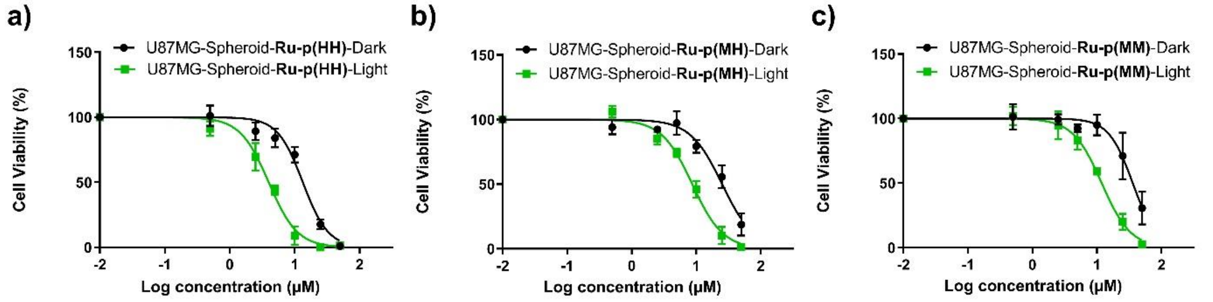

Figure S30. Dose-response curves for U87MG 3D tumor spheroids incubated with complex **Ru-p(HH)**, **Ru-p(MH)** and **Ru-p(MM)**in the dark (in black) or irradiated with green light (in green, 520 nm, 13.1 J cm^{-2}).

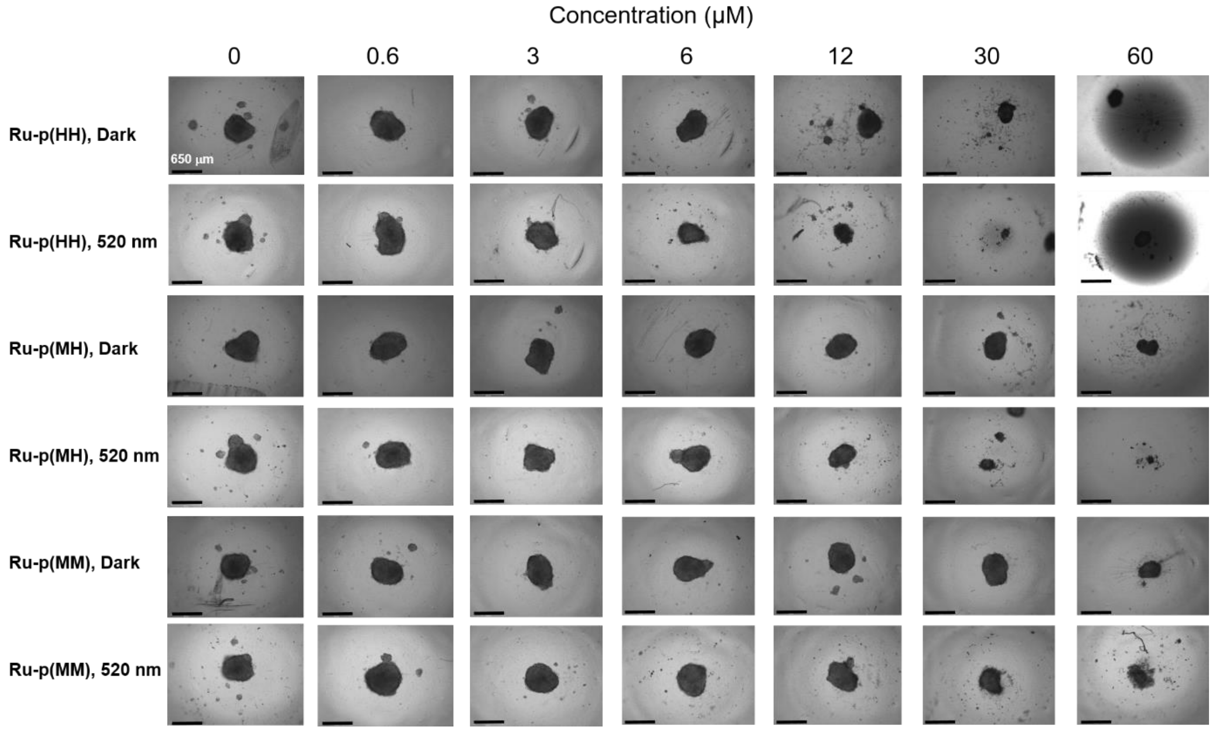

Figure S31. Bright field images of U87MG 3D tumor spheroids treated with different concentrations of **Ru-p(HH)**, **Ru-p(MH)** and **Ru-p(MM),** and left in the dark or irradiated with green light (520 nm, 13.1 J.cm^{-2}). Scale bar = 650 μm.

12 Drug distribution in zebrafish model

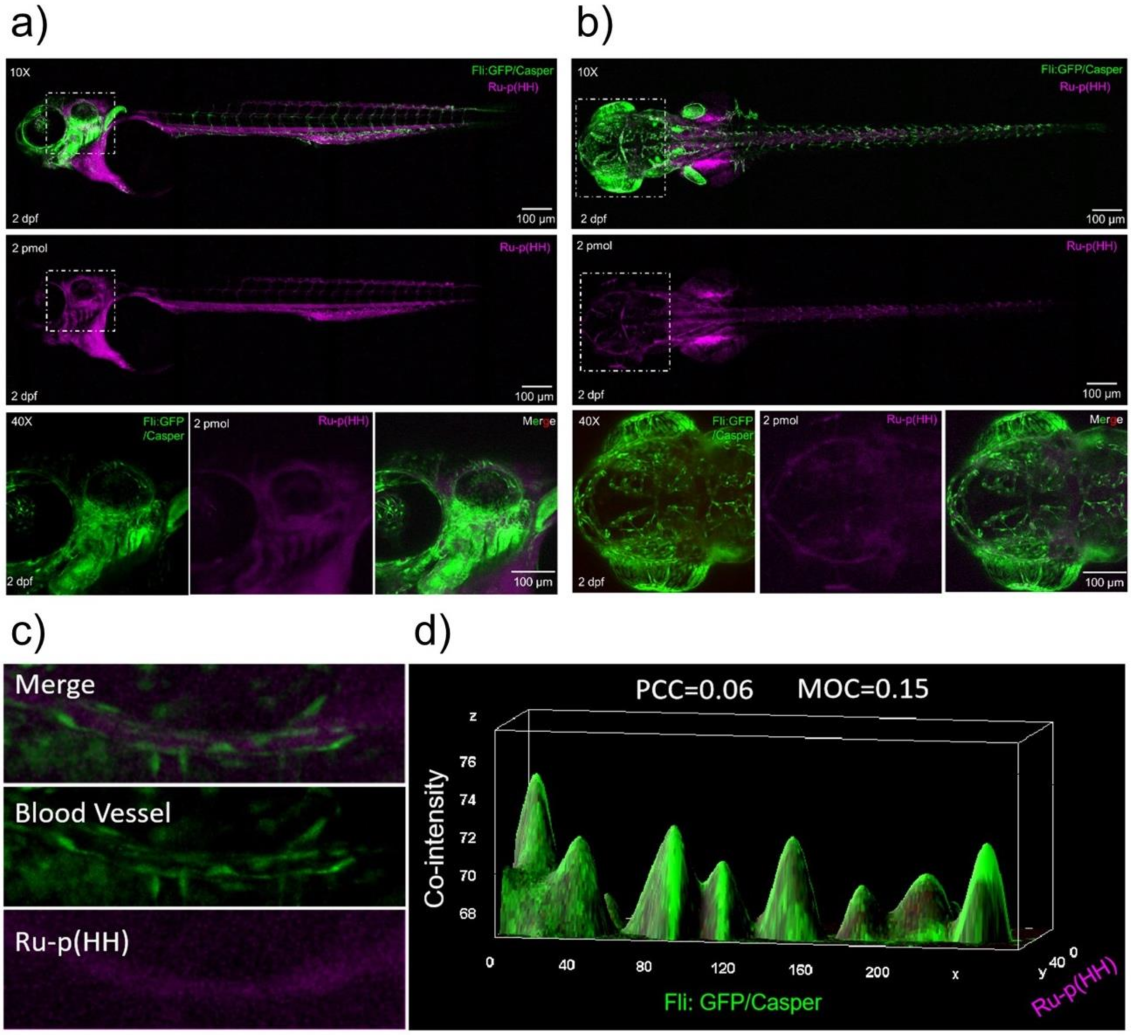

Figure S32. Biodistribution of **Ru-p(HH)** (2 pmol) 4 h after intravenous injection in two-days-post fertilization (2 dpf) embryonic zebrafish. (a) Confocal micrograph (side view) (10x) of distribution of **Ru-p(HH)** (excitation: 488nm; emission: 485-575nm) in whole Tg(*fli1*:eGFP)/Casper zebrafish embryos. Magnification of the drug distribution in hindbrain (40X) (N=5). (b) Distribution image of **Ru-p(HH)** in the whole zebrafish embryo (top view) (10X) and local distribution image of hindbrain (40X), Scale bar = 100 µm. (c) The blood vessels in panel b are partially enlarged to show the vascular channel (green) and the **Ru-p(HH)** channel (magenta). (d) The mountain map represents the co-localization analysis of **Ru-p(HH)** (purple) and Tg(*fli1*:eGFP)/Casper (green) in the top view of the embryo. PCC is 0.06, MOC is 0.15.

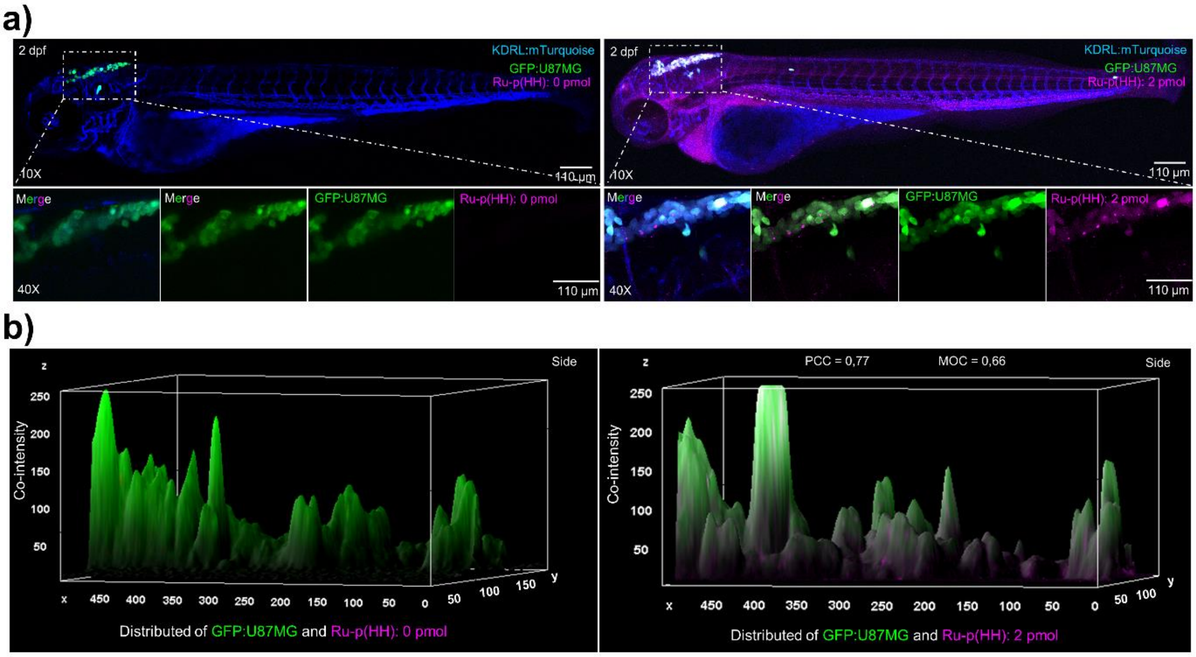

Figure S33. Targeting of **Ru-p(HH).** (a) GFP-U87MG (green) were injected into the hindbrain of Tg(*kdrl*:mTurquoise) (blue) zebrafish embryos, one hour after tumor injection, vehicle control (DMSO) and Ru-p(HH) (deep red) was injected into the zebrafish embryo by intravenous (IV) injection. After another 4 h, images of whole zebrafish embryo (10X) and high-resolution (40X) images of hindbrain were taken by confocal microscopy. Scale bar = 110 µm. (b) The mountain map in the upper figure represents the colocalization analysis of Ru-p(HH) and U87MG in the frontal image of zebrafish embryo, while the following figure represents the colocalization analysis of the lateral image of zebrafish embryo. The distribution of the drug (purple) and the cell signal (red) are shown; the higher the overlapping areas of green and purple, the larger the colocalization. The Z-axis is the cell fluorescence intensity (green), and the drug fluorescence intensity (purple), and the higher the intensity of purple and green in the same mountain, the stronger the intensity and colocalization. Both PCC and MOC are calculated and shown in the graph.

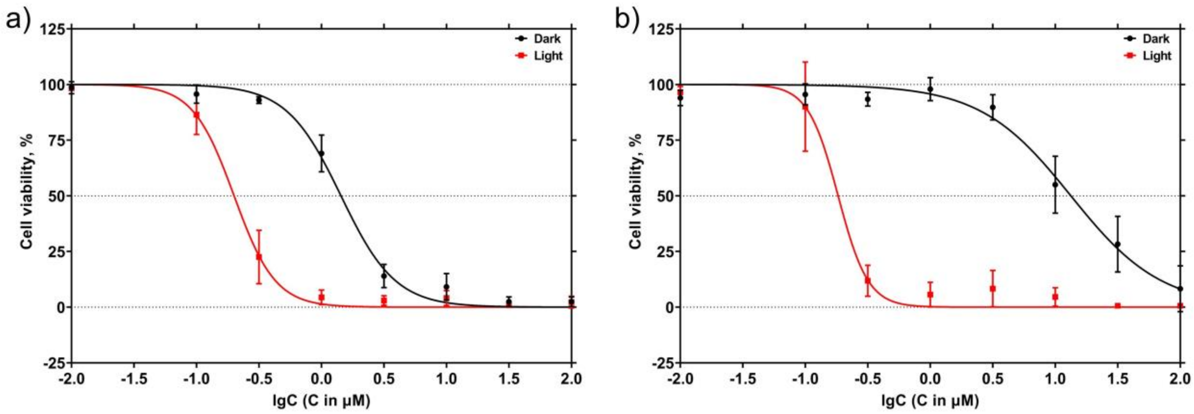

Figure S34. Dose-response curves for **$[Ru(Ph_2phen)_2Cl_2]$** (a) and **$[Ru(Ph_2phen)_2(bpy)]Cl_2$** (b) in the dark and upon 520 nm green-light irradiation in normoxic U87MG cell line (10.9 mW cm^{-2}, 13.1 J cm^{-2}, 20 min). Dose-response curves were obtained by two parametric Hill-slope evaluation. For every experiment it was ensured that neither 0.4% DMSO or light itself causing any significant reduction in cells population.

Table S1. EC_{50} data for **$[Ru(Ph_2phen)_2Cl_2]$** and **$[Ru(Ph_2phen)_2(bpy)]Cl_2$** in U87MG cancer cell line in the dark (D) and after 520 nm green-light irradiation (GL) under normoxia, photoindexes calculated as PI = EC_{50D}/EC_{50GL}.

**Compound**	**Light**	**EC_{50} (±95% CI) in normoxic U87MG, μM**	**PI**
**$[Ru(Ph_2phen)_2Cl_2]$**	D	1.43 (+0.18/–0.16)	**7.2**
	GL	0.20 (+0.02/–0.02)	
**$[Ru(Ph_2phen)_2(bpy)]Cl_2$**	D	13.4 (+3.69/–2.91)	**74.4**
	GL	0.18	

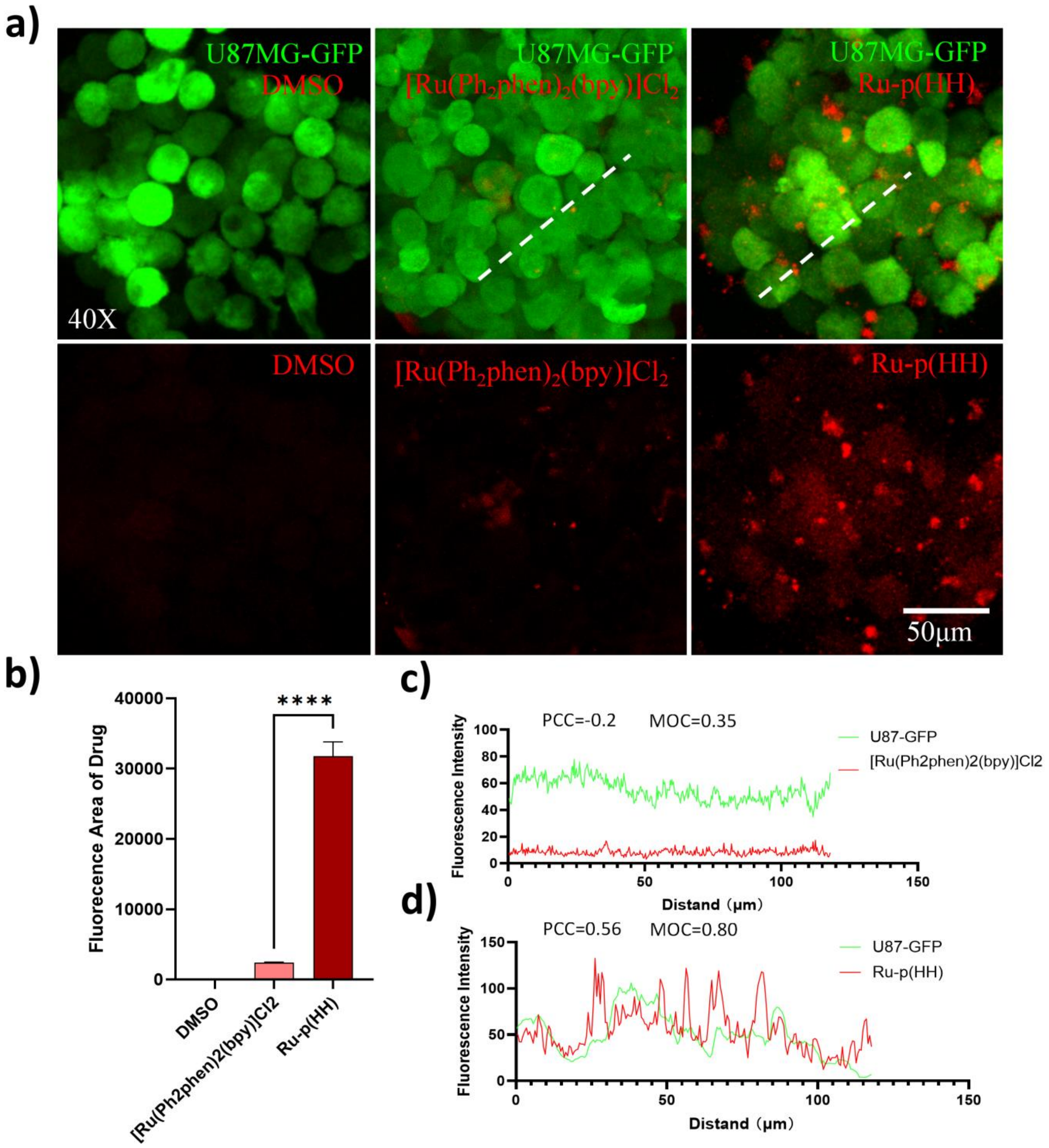

Figure S35. Targeting of **$[Ru(Ph_2phen)_2(bpy)]Cl_2$** and **Ru-p(HH)** to tumor cells in Zebrafish. (a) High-resolution images (40X) of U87MG-GFP of zebrafish injected with DMSO, **$[Ru(Ph_2phen)_2(bpy)]Cl_2$** and **Ru-p(HH)** (dosage: 1 nL×2 mM = 2 pmol), respectively. Scale bar = 50 μm. (b) In cellular regions (green channel in a), the statistical distribution area of drugs (red channel in a). T-test was used to determine the significance of data indicated in a (*P < 0.05; **P < 0.01; ***P < 0.001; ****P < 0.0001). In all panels, error bars represent mean±s.d. (N=3). c) and d) Line-scan profiles of fluorescence intensity from the white line in a. Pearson Correlation Coefficient (PCC) and Mander's Overlap Coefficient (MOC) were calculated

separately to indicate colocalization. PCC was -0.2 and the MOC was 0.35 in c), PCC is 0.56 and MOC is 0.80 in d).

13 Cytotoxicity study on zebrafish

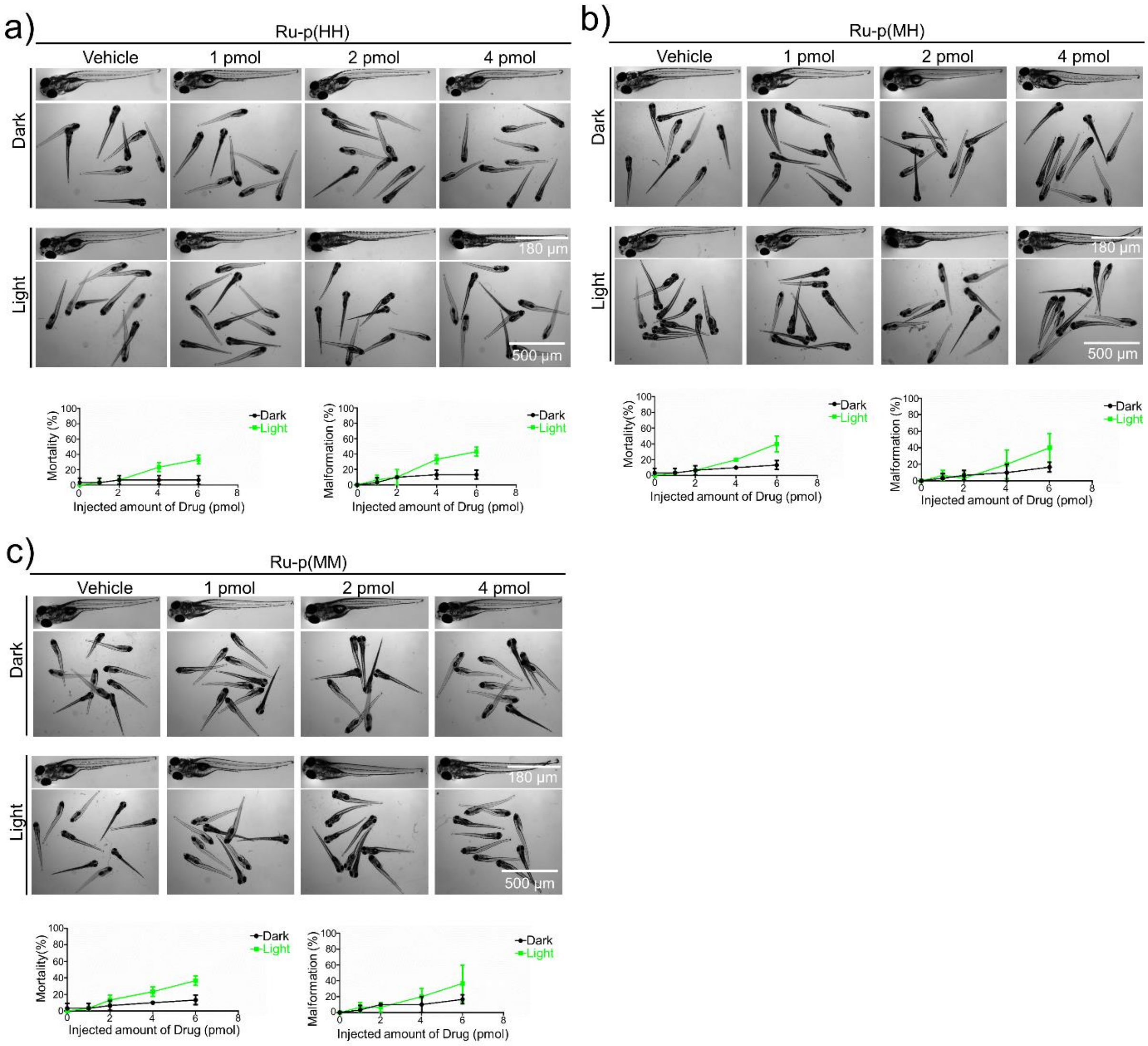

Figure S36. Stereo microscope images of zebrafish treated with different amount of **Ru-p(HH)**, **Ru-p(MH)** and **Ru-p(MM)** in the dark (black) or irradiated with green light (in green, 520 nm, 78.5 J cm^{-2}).

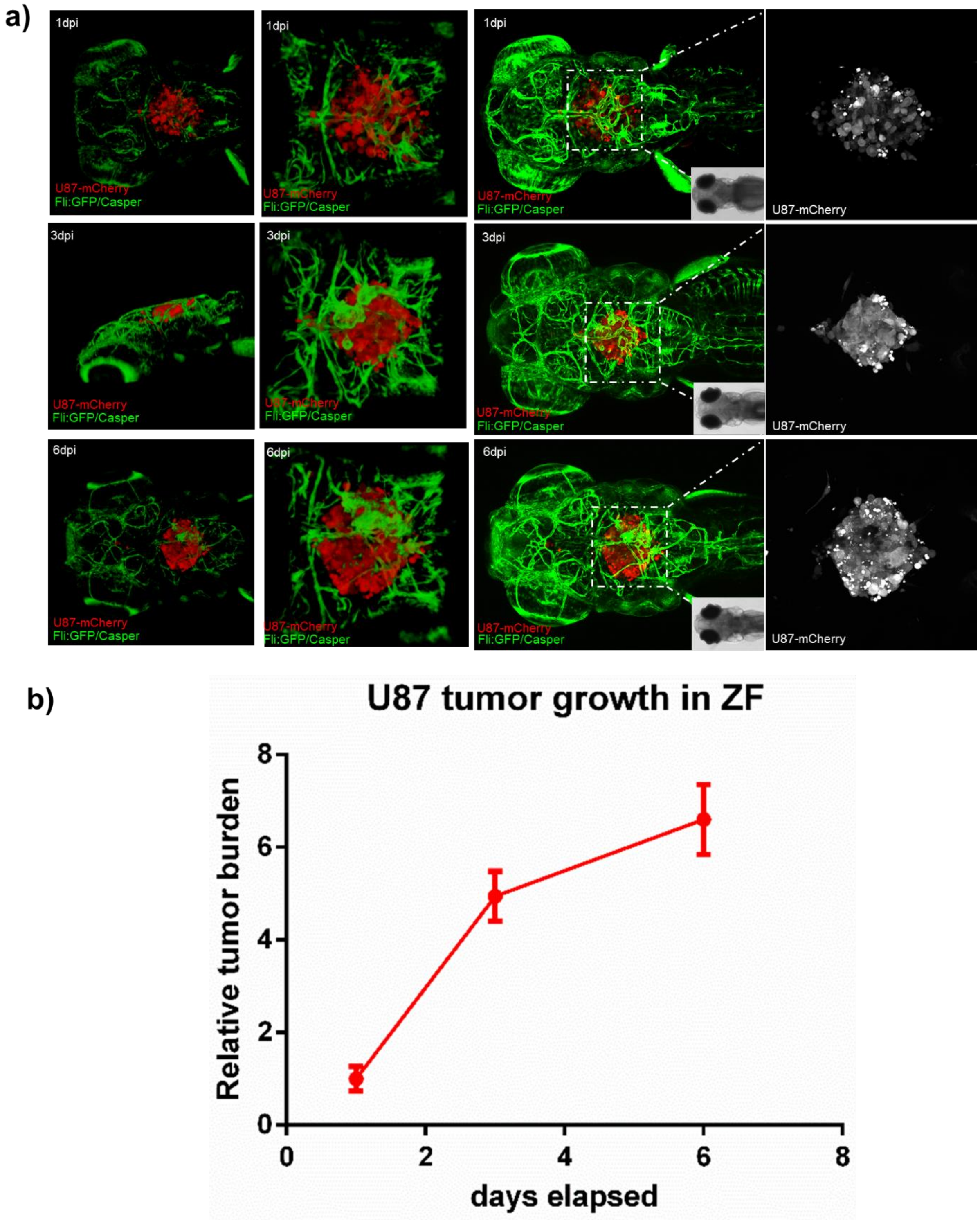

Figure S37. Tumor xenograft growth in zebrafish. (a) SP8 confocal microscope imaged the 3D and 2D of U87MG (in red) growth in the hindbrain (blood vessel in green) at 3 dpf, 5 dpf, and 8 dpf. (b) The means intensity of U87 in the hindbrain of zebrafish at 3 dpf, 5 dpf, and 8 dpf

normalize to the means at 3 dpf (n=5). All data were analyzed by image J. And graphs were generated by GraphPad.

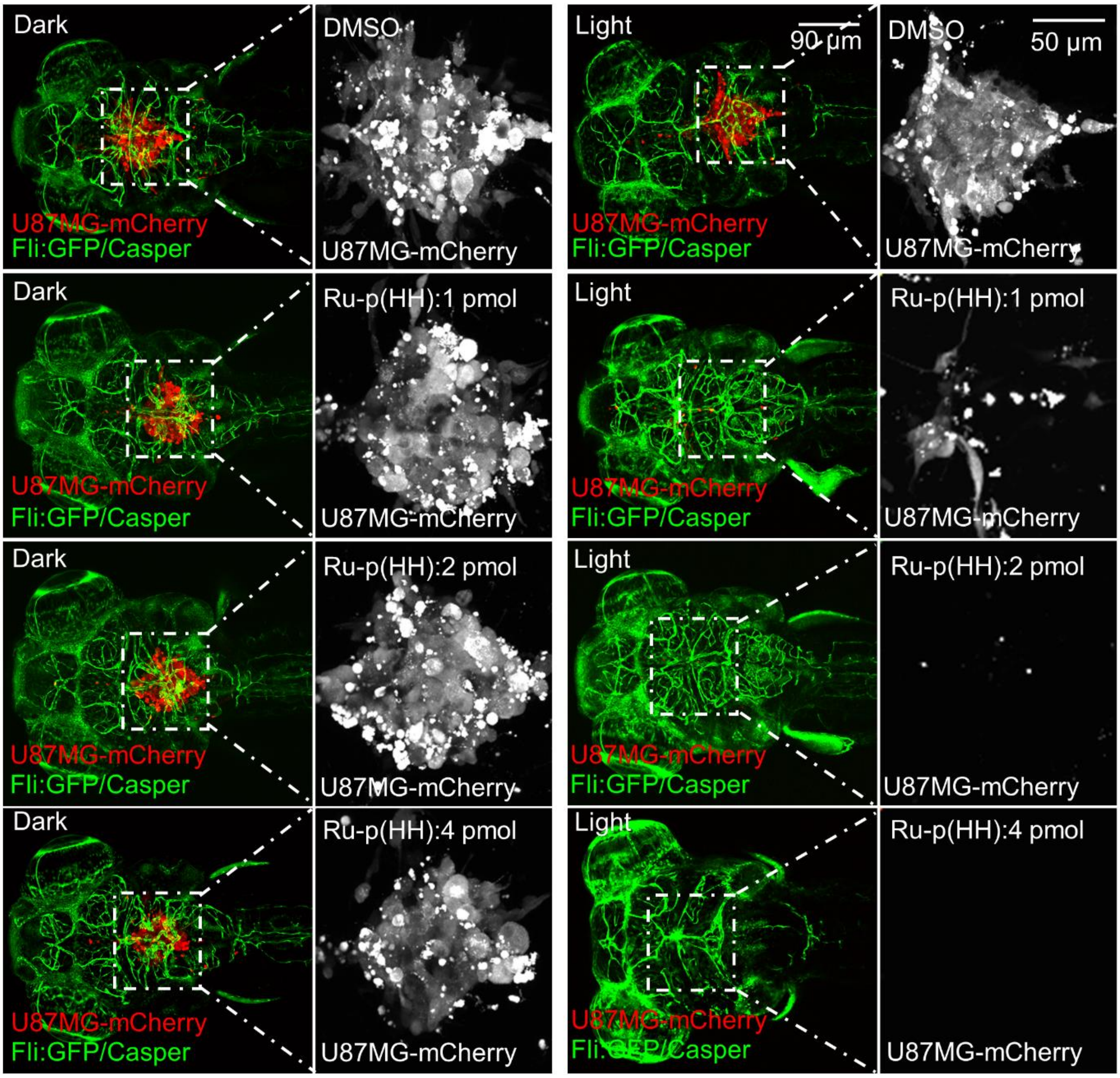

Figure S38. Therapeutic anti-tumor effect of the different photo-activated drug. Dose-response for U87MG tumor burden in zebrafish hindbrain (8 dpf) incubated with **Ru-p(HH)** (1 pmol, 2 pmol or 4 pmol) in the dark (black) or irradiated with green light (green, 520 nm, 78.5 J cm^{-2}).

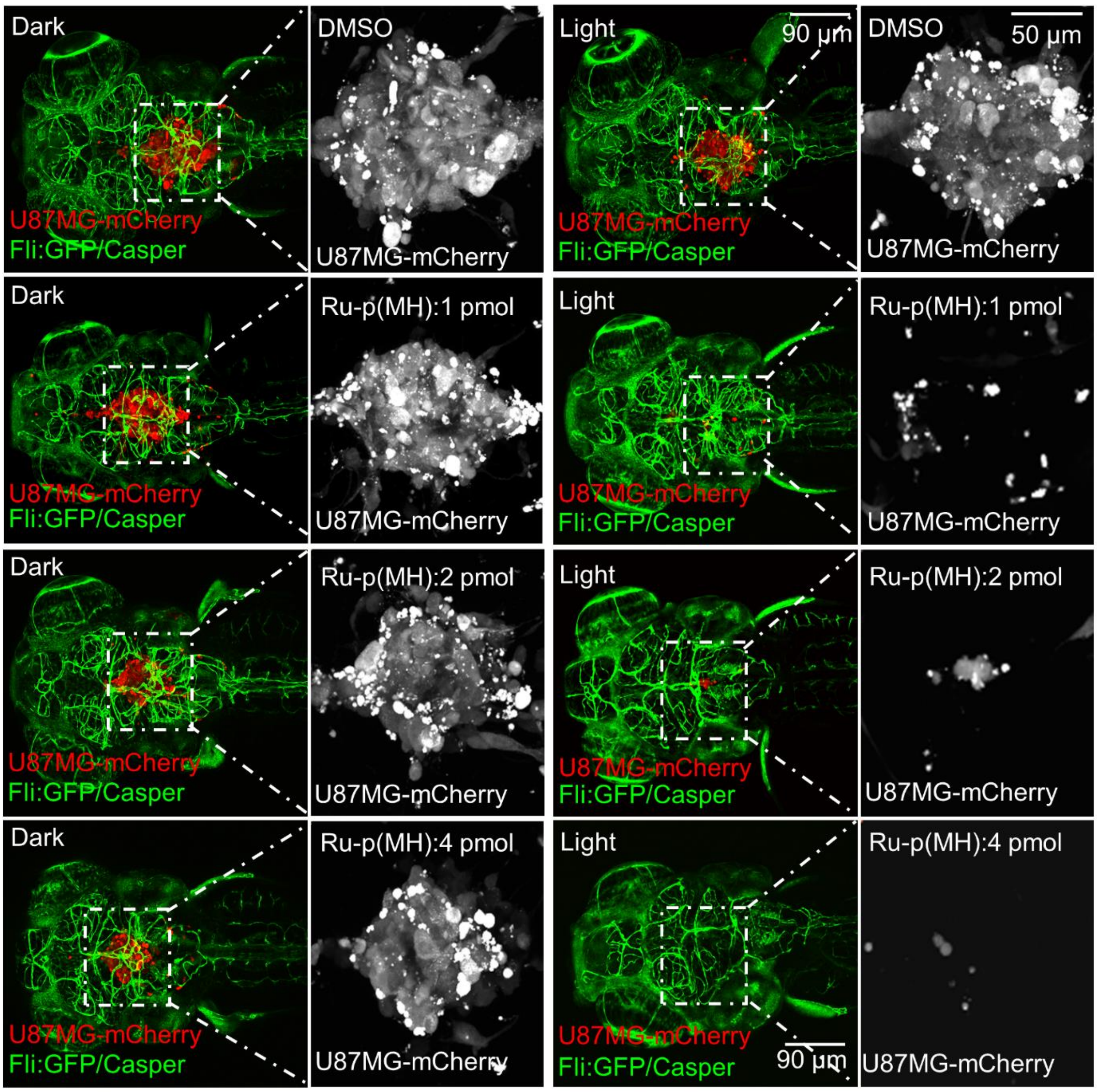

Figure S39. Therapeutic anti-tumor effect of the different photo-activated drug. Dose-response (1 pmol, 2 pmol and 4 pmol) for U87MG tumor burden in zebrafish hindbrain (8dpf) incubated with **Ru-p(MH)** in the dark (black) or irradiated with green light (in green, 520 nm, 78.5 J cm^{-2}).

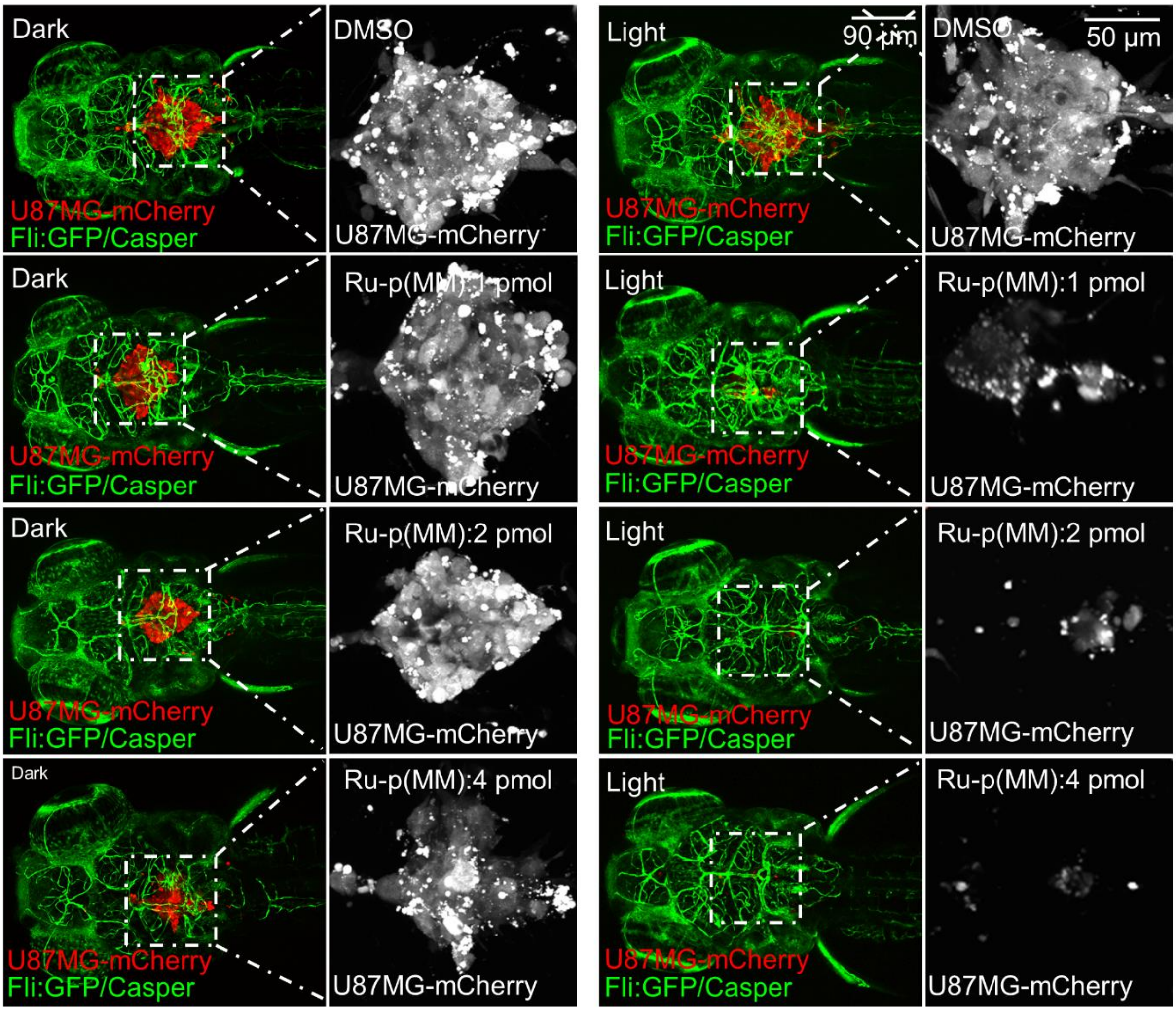

Figure S40. Therapeutic anti-tumor effect of the different photo-activated drug. Dose-response (1 pmol, 2 pmol and 4 pmol) for U87MG tumor burden in zebrafish hindbrain (8dpf) incubated with **Ru-p(MM)** in the dark (black) or irradiated with green light (in green, 520 nm, 78.5 J cm^{-2}).

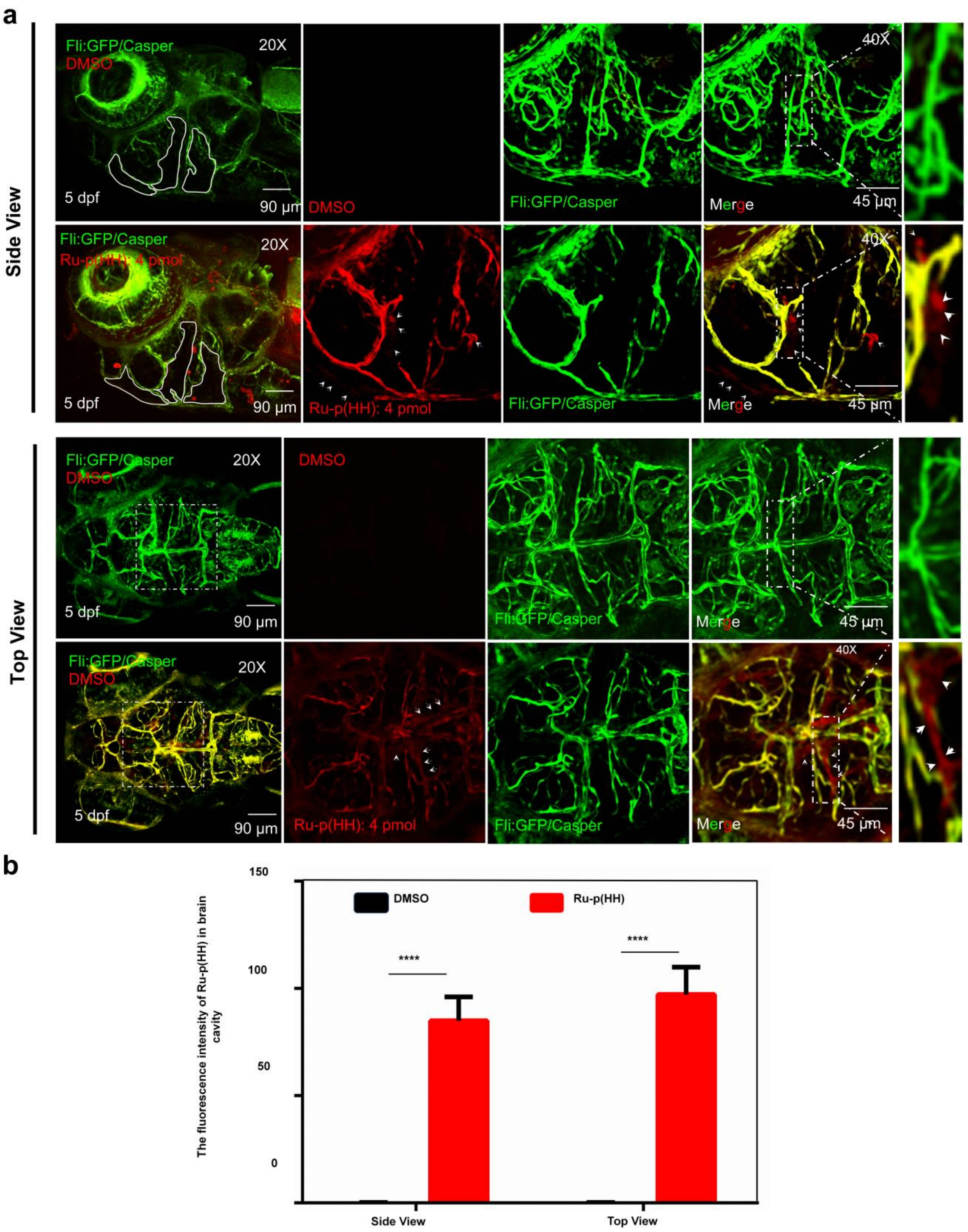

Figure S41. (a) The brain distribution of intravenously administered **Ru-p (HH)** ((dosage: 1 nL×4 mM = 4 pmol, λEx/λEm = 488/620-680 nm) in 5 dpf zebrafish analyzed by high-resolution confocal microscopy. The arrow points to **Ru-p (HH)** which passes through the BBB into the brain cavity. (b). NIR emission intensity of Ru-p(HH) in the brain cavity at 5 dpf. N=3 fishes in each group.

14 Overview figure

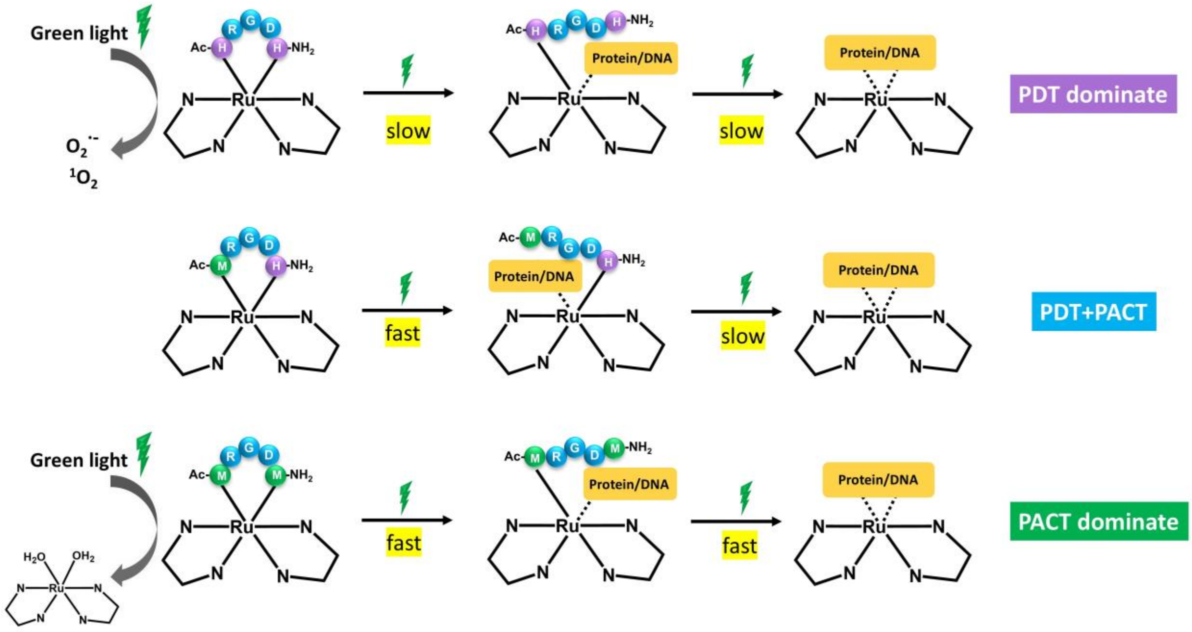

Figure S42. Scheme of Ru-peptide conjugates as photoactive anticancer drugs with different mechanisms.

Reference

1. Zhang, L.; Wang, P.; Zhou, X.-Q.; Bretin, L.; Zeng, X.; Husiev, Y.; Polanco, E. A.; Zhao, G.; Wijaya, L. S.; Biver, T., Cyclic ruthenium-peptide conjugates as integrin-targeting phototherapeutic prodrugs for the treatment of brain tumors. *Journal of the American Chemical Society* **2023,** *145* (27), 14963-14980.
2. Cuello-Garibo, J.-A.; Meijer, M. S.; Bonnet, S., To cage or to be caged? The cytotoxic species in ruthenium-based photoactivated chemotherapy is not always the metal. *Chemical Communications* **2017,** *53* (50), 6768-6771.
3. Howerton, B. S.; Heidary, D. K.; Glazer, E. C., Strained ruthenium complexes are potent light-activated anticancer agents. *Journal of the American Chemical Society* **2012,** *134* (20), 8324-8327.
